# Authentication and validation of key genes in the treatment of atopic dermatitis with Runfuzhiyang powder: combined RNA-seq, bioinformatics analysis, and experimental research

**DOI:** 10.3389/fgene.2024.1335093

**Published:** 2024-08-01

**Authors:** Yan Lin, Guangyi Xiong, Xiansong Xia, Zhiping Yin, Xuhui Zou, Xu Zhang, Chenghao Zhang, Jianzhou Ye

**Affiliations:** ^1^ Department of Dermatology, The No.1 Affiliated Hospital of Yunnan University of CM, Kunming, China; ^2^ Biology and Medical Statistic Unit, Basic Medical Science School, Yunnan University of CM, Kunming, China; ^3^ Teaching Affairs Department, Yunnan University of CM, Kunming, China; ^4^ Department of Laboratory Medicine, The No.1 Affiliated Hospital of Yunnan University of CM, Kunming, China

**Keywords:** atopic dermatitis, Runfuzhiyang powder, diagnosis, biomarkers, immune infiltration, TF–mRNA–lncRNA network

## Abstract

**Background:**

Atopic dermatitis (AD) is inflammatory disease. So far, therapeutic mechanism of Runfuzhiyang powder on AD remains to be studied. This study aimed to mine key biomarkers to explore potential molecular mechanism for AD incidence and Runfuzhiyang powder treatment.

**Methods:**

The control group, AD group, treat group (AD mice treated with Runfuzhiyang powder were utilized for studying. Differentially expressed AD-related genes were acquired by intersecting of key module genes related to control group, AD group and treatment group which were screened by WGCNA and AD-related differentially expressed genes (DEGs). KEGG and GO analyses were further carried out. Next, LASSO regression analysis was utilized to screen feature genes. The ROC curves were applied to validate the diagnostic ability of feature genes to obtain AD-related biomarkers. Then protein-protein interaction (PPI) network, immune infiltration analysis and single-gene gene set enrichment analysis (GSEA) were presented. Finally, TF-mRNA-lncRNA and drug-gene networks of biomarkers were constructed.

**Results:**

4 AD-related biomarkers (Ddit4, Sbf2, Senp8 and Zfp777) were identified in AD groups compared with control group and treat group by LASSO regression analysis. The ROC curves revealed that four biomarkers had good distinguishing ability between AD group and control group, as well as AD group and treatment group. Next, GSEA revealed that pathways of E2F targets, KRAS signaling up and inflammatory response were associated with 4 biomarkers. Then, we found that Ddit4, Sbf2 and Zfp777 were significantly positively correlated with M0 Macrophage, and were significantly negatively relevant to Resting NK. Senp8 was the opposite. Finally, a TF-mRNA-lncRNA network including 200 nodes and 592 edges was generated, and 20 drugs targeting SENP8 were predicted.

**Conclusion:**

4 AD-related and Runfuzhiyang powder treatment-related biomarkers (Ddit4, Sbf2, Senp8 and Zfp777) were identified, which could provide a new idea for targeted treatment and diagnosis of AD.

## 1 Introduction

Atopic dermatitis (AD) is a prevalent inflammatory skin disorder with a rising global incidence, significantly impairing patients’ quality of life. AD manifests as recurrent eczematous lesions and intense pruritus, stemming from a dysfunctional epidermal barrier, an abnormal skin microbiome, and dysregulated type 2 immune mechanisms (Langan et al., 2020). This heterogeneous disease involves multiple genetic risk factors, with over 70 genes implicated, including those related to skin barrier function, T helper (Th)2 immune responses, vitamin D metabolism, and its receptor synthesis. However, genetic predispositions play a minor role in AD onset. Notably, individuals with mutations in the filaggrin (FLG) gene or other relevant genes do not always exhibit classic AD symptoms, and some patients lack identifiable mutations. Moreover, the epigenomes of AD patients differ significantly from those of healthy individuals, particularly in genes regulating immune responses and inflammatory processes (Nedoszytko et al., 2020). The heterogeneity of AD endotypes and phenotypes complicates the establishment of distinct biomarkers. Current diagnostic criteria and measurement tools often lack clinical applicability in specific subpopulations and are susceptible to inter-observer biases. This heterogeneity may also contribute to the limited efficacy of traditional treatment methods. Therefore, identifying reliable biomarkers is crucial for improved diagnosis and monitoring of AD patients. Additionally, a better understanding of the condition could facilitate the development of multifactorial treatments for this complex and heterogeneous disease.

Cutaneous inflammation, particularly type 2 skin inflammation, is central to the pathogenesis of AD. The lesional skin exhibits a complex and diverse inflammatory profile. Beyond Th1 and Th2 cells, AD lesions contain a variety of immune cells, including Th17 and Th22 cells (Brunner et al., 2018), dendritic cells, macrophages, neutrophils, natural killer (NK) cells (Rojahn et al., 2020), innate lymphoid cells, inflammatory epidermal dendritic cells (IDECs), basophils (Mashiko et al., 2017), and eosinophils (Chiricozzi et al., 2023). Managing AD involves alleviating symptoms and establishing long-term control by avoiding individual triggers, restoring the skin barrier with moisturizers, and employing a step-up and step-down approach to reduce inflammation. Anti-inflammatory therapy is chosen based on disease severity. A deeper understanding of the key drivers of inflammation in AD is essential for developing targeted therapeutic approaches. Additionally, these therapies should be individualized, considering each patient’s unique characteristics and disease course.

Runfuzhiyang powder, formulated by the late Prof. Liu Fuxing, an expert in Chinese medicine with decades of clinical experience, is composed of *Pogostemon cablin*, *Elsholtzia*, *Herba artemisiae scoparia*, and *Speranskia* herb. This mixture is decocted into a solution and applied as a wet compress. Runfuzhiyang powder has a long history of clinical use, known for its ability to improve lesions and reduce pruritus (Guo and Ye, 2011). However, the specific effects and the molecular mechanisms through which Runfuzhiyang powder acts have not yet been studied.

This study aims to elucidate the molecular mechanisms through which Runfuzhiyang powder treats AD. RNA sequencing was performed on cutaneous samples from AD mice, *Runfuzhiyang* powder-treated AD mice, and control mice. Key genes were identified, and a molecular regulatory network associated with these genes was constructed. Additionally, the study analyzed immune cell infiltration in AD.

## 2 Materials and methods

### 2.1 Grouping and animal models

**FIGURE 1:**
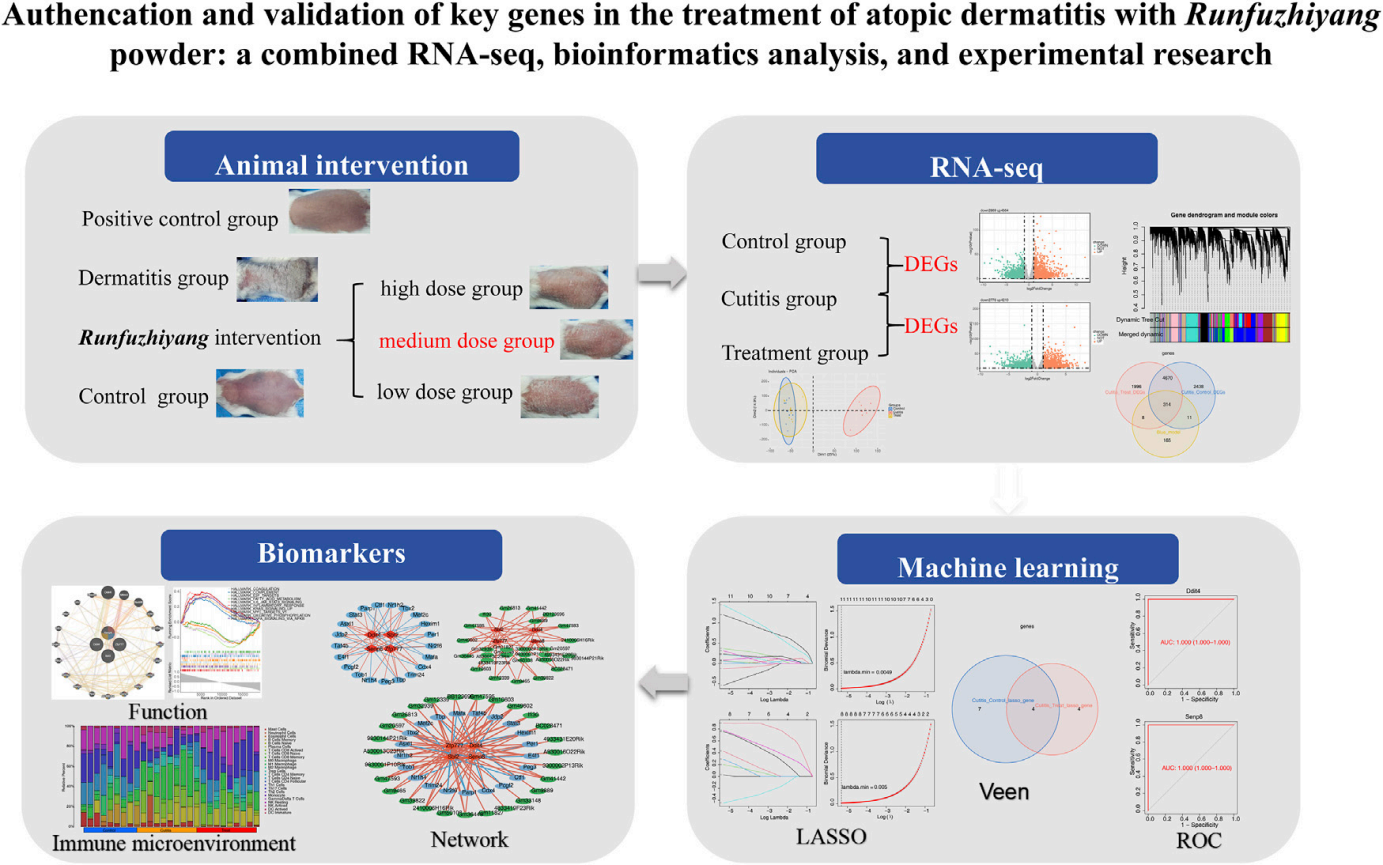
Flowchart.

Fifty-four female Balb/c mice (6 to 8 weeks old) were used in this study to investigate the effects of Runfuzhiyang powder on dermatitis. The mice were divided into four groups: the control group (n = 10), AD group (n = 10, representing the dermatitis group), treatment group (n = 30, AD-like mice treated with Runfuzhiyang powder), and positive control group (n = 4, AD-like mice treated with a known positive therapeutic drug). The treatment group was further divided into three subgroups to determine the optimal concentration of the intervention: low dose (n = 10), medium dose (n = 10), and high dose (n = 10). The control group was maintained normally after shaving their backs. To induce AD-like dermatitis, the back of each mouse was coated with 50 μL of 3 nmol of calcipotriol (MC903) dissolved in 95% ethanol, followed by 25 μL of 20 g/L ovalbumin. This induction process was carried out for 7 days. Starting from day 7, the AD-like mice were treated with different concentrations of Runfuzhiyang powder for 10 min daily. The positive control group received a known therapeutic drug for 10 min daily. The control and dermatitis groups were treated with normal saline for 10 min daily. Dermatitis scores and scratching behavior were recorded at 7, 14, and 21 days across the different groups. The study plan is illustrated in Figure 1.

### 2.2 Histopathological analysis

The back skin tissue from the mice was harvested and fixed in 4% paraformaldehyde for 24–48 h. After embedding in paraffin, the tissue was sectioned into 5-μm-thick slices for hematoxylin–eosin (H&E) and toluidine blue staining. For H&E staining, tissue sections were stained with hematoxylin for 5 min, treated with the 1% hydrochloric acid alcohol differentiation solution for 10–15 s, and then stained with eosin for 5–10 s. Subsequently, the sections underwent two treatments with xylene (10 min each), following dehydration with ethanol. For toluidine blue staining, sections were stained with toluidine blue for 10–15 min and then treated with the 0.1% glacial acetic acid differentiation solution for 5–10 s. After dehydration with ethanol, the sections were treated with xylene for 10 min. Finally, the sections were mounted with balsam to observe the histomorphology of the back skin tissue under a microscope (TS2, Nikon, Japan). Mast cells and eosinophils were counted in at least five fields by flow cytometry.

### 2.3 Measurement of serum immunoglobulins

Serum immunoglobulin E (IgE) levels were measured using enzyme-linked immunosorbent assay (ELISA) kits (Jianglai Biotechnology, Suzhou, China), following the manufacturer’s instructions.

### 2.4 Immunofluorescence co-localization

After fixation, dehydration, transparency, and wax immersion, the skin tissue was embedded in paraffin and cut into 3-µm sections. The sections were baked, dewaxed, and hydrated, followed by antigen retrieval and endogenous peroxidase inactivation. For nuclear staining, a DAPI staining solution was added dropwise to the cells, stained for 5 min at room temperature while protected from light, and then washed three times with PBS for 5 min each. To detect Ddit4, CD56, and CD68, their respective antibodies (Santa, Sc-271158; NLAMI, DF 1832; and Affinity, DF7581) were diluted and incubated with the sections. Following incubation with appropriate secondary antibodies, the slices were sealed with an anti-fluorescence-quenching blocker. Five fields of view were selected for observation under the microscope, and the slices were photographed to calculate the positive rate.

### 2.5 Acquisition and processing of samples

The diseased skin of AD mice (AD group; cutitis group), the skin of healthy mice (control group), and the skin of AD mice treated with Runfuzhiyang powder (treatment group) were collected, with nine cases in each group.

### 2.6 RNA extraction, library construction, and sequencing

TRIzol (Invitrogen, CA, United States) was used to isolate and purify RNA. The quality of RNA was assessed using the NanoDrop ND-1000 spectrophotometer (NanoDrop, Wilmington, DE, United States), and its integrity was confirmed with the Bioanalyzer 2100 instrument (Agilent, CA, United States). For downstream experiments, 1 μg of RNA was deemed sufficient. PolyA-containing mRNA was captured using oligo(dT) magnetic beads (Dynabeads Oligo(dT), 25-61005, Thermo Fisher, United States). The captured mRNA was fragmented using the NEBNext^®^ Magnesium RNA Fragmentation Module (E6150S, United States) at 94°C for 5–7 min. Fragmented RNA was then used to synthesize cDNA with reverse transcriptase (Invitrogen SuperScript™ II Reverse Transcriptase, 1,896,649, CA, United States). RNase H (NEB, m0297, United States) and *E. coli* DNA polymerase I (NEB, m0209, United States) converted RNA–DNA hybrids into DNA duplexes through two-strand synthesis. The double-stranded DNA was mixed in a dUTP solution (Thermo Fisher, R0133, CA, United States), and its ends were blunted. Magnetic beads were used to screen and purify the fragments by size. A library of 300 ± 50-bp fragments was created by digesting the double-stranded DNA with the UDG enzyme (NEB, m0280, MA, United States). Finally, two-end sequencing was performed using the Illumina Novaseq™ 6000 system (LC-Bio Technologies Co., Ltd., Hangzhou, China) in the PE150 mode according to standard protocols.

### 2.7 Sequencing data quality control and preprocessing

First, low-quality sequencing data were filtered using the Cutadapt (v. 1.9) package. The following parameters were removed: 1) adapters, polyA, and polyG; 2) sequences with more than 5% unknown nucleotides (N); and 3) low-quality reads containing more than 20% bases with a Q-value ≤20. Sequence quality was then verified using FastQC (v. 0.11.9), which included assessing Q20, Q30, and GC content of the clean data. The filtered clean data were subsequently aligned to the reference mouse genome using the HISAT2 (v. 2.2.1) package (Kim et al., 2015). The reference mouse genome was sourced from the Ensembl database (https://www.ensembl.org/Mus_musculus/Info/Index?redirect=no). Gene counts were extracted using featureCount software.

### 2.8 Identification of differentially expressed genes (DEGs)

To identify DEGs between the different groups, differential expression analysis was performed. First, the gene expression matrix of each sample was extracted and standardized using DESeq2 software. A box plot of the gene expression matrix was then generated. Principal component analysis (PCA) was applied to illustrate differences between the samples of different groups. DEGs were identified in three groups (the cutitis group, control group, and treatment group) using the “DESeq2” package with criteria of |log_2_ fold change (FC)| > 1.5 and p < 0.05 (Love et al., 2014). A volcano plot was created using the R package “ggpubr” (Kassambara, 2023), and heatmaps were generated with the R package “Heatmap” (Boileau, 2019).

### 2.9 Weighted gene co-expression network analysis (WGCNA)

WGCNA, a bioinformatics algorithm, integrates highly related genes into several modules. Using the R package “WGCNA” (Langfelder and Horvath, 2008), modules highly related to AD were selected. Genes in the modules were related to the cutitis group, control group, and treatment group. First, the goodSamplesGenes function of the “WGCNA” package facilitated sample clustering and filtering. Next, the data’s soft threshold was determined. Each gene module was set to a minimum size of 300, following the hybrid dynamic tree cut algorithm. Genes were divided into modules through hierarchical clustering. The correlation between each module and the experimental groups was analyzed, and the module with the highest absolute correlation coefficient with traits was identified as the key module for further analysis.

### 2.10 Screening and enrichment analysis of candidate genes

To identify the candidate genes involved in Runfuzhiyang powder’s treatment of AD rats, the intersection of DEGs from the cutitis group vs control group, DEGs from the cutitis group vs treatment group, and module genes was obtained using a Venn diagram. The biological functions and pathway analyses of these candidate genes were performed using the Kyoto Encyclopedia of Genes and Genomes (KEGG) and Gene Ontology (GO) with the R package “clusterProfiler” (Wu et al., 2021).

### 2.11 Screening of biomarkers and receiver operating characteristic (ROC) curve analysis

First, the least absolute shrinkage and selection operator (LASSO) regression analysis was used to screen feature genes in the cutitis group vs control group and in the cutitis group vs treatment group. The feature genes identified in both groups were then intersected to obtain biomarkers. Finally, ROC curves were plotted to validate the diagnostic value of these biomarkers.

### 2.12 Construction of the gene–gene interaction (GGI) network and gene set enrichment analysis (GSEA)

Using the GeneMANIA online database, genes related to the identified biomarkers were predicted. The top 20 genes, along with the four biomarkers, were then used to construct a GGI network with the R package “Cytoscape” (Smoot et al., 2011). The functions of these genes were annotated under the GGI network. Additionally, GSEA was conducted to explore the pathways associated with the biomarkers. Spearman’s correlation coefficients between the biomarkers and all other genes were calculated and ranked. GSEA showed significant enrichment in KEGG pathways using the R package “clusterProfiler” (P-adjust <0.05) (Wu et al., 2021). The background gene set “c2.cp.kegg.v7.0.symbols.gmt” was downloaded from the Molecular Signatures Database (MSigDB).

### 2.13 Immune microenvironment and correlation analysis

The immune microenvironment was explored in the three groups. Initially, 25 immune-cell gene sets from mice were selected as the reference gene set. The CIBERSORT algorithm (Newman et al., 2015) was employed to determine the proportion of immune cells. Differential immune cells were then analyzed in the cutitis group vs control group and in the cutitis group vs treatment group. Finally, Spearman’s correlation analysis was performed between the biomarkers and differential immune cells.

### 2.14 Analysis of the transcription factor (TF)–mRNA–long non-coding RNA (lncRNA) network

To identify potential molecules involved in the regulation of biomarkers, TFs and lncRNAs were predicted based on these biomarkers. From the transcriptional regulatory relationships database (TRRUST, http://www.grnpedia.org/trrust), 827 mouse TFs were obtained. The correlation coefficient between the TFs and biomarkers was calculated using Spearman’s method. Biomarker-related TFs were identified with a correlation coefficient |cor| > 0.9 and p < 0.05. Next, intersections of biomarker-related TFs and DEGs in the cutitis group vs control group were used to obtain candidate TFs, resulting in TF–mRNA pairs.

Differential expression (DE)-lncRNAs in the cutitis group were identified using the DESeq2 package and compared with those in the control group. Spearman’s method was employed to calculate the correlation between DE-lncRNAs and biomarkers. Biomarker-related lncRNAs were selected based on a correlation coefficient |cor| > 0.9 and p < 0.05. Intersections of biomarker-related lncRNAs and DE-lncRNAs were then used to identify candidate lncRNAs, resulting in lncRNA–mRNA pairs. Finally, a TF–mRNA–lncRNA network was constructed.

### 2.15 Drug prediction

In addition to Runfuzhiyang powder, other drugs associated with the identified biomarkers were predicted for potential use in subsequent AD treatment. Initially, the biomarkers were transformed from mouse genes into their human homologous genes. A drug–gene network was then constructed using the Drug Gene Interaction database (DGIDB, www.dgidb.org).

### 2.16 Statistical analysis

All bioinformatics analyses were conducted using R software. For the experimental analysis, a one-way ANOVA test was employed to compare differences between two variables using GraphPad Prism (v. 10.0) software. DEGs were identified with criteria of |log_2_FC| > 1.5 and p < 0.05. LASSO regression analysis was utilized to identify feature genes. The diagnostic value was assessed using the ROC curve, with an area under the ROC curve (AUC) > 0.7 indicating good diagnostic performance. Spearman’s correlation analysis was performed to evaluate the relationship between two variables, with |cor| > 0.3 and p < 0.05 considered significant. Additionally, a p-value of <0.05 was regarded as indicative of a statistically significant difference.

## 3 Results

### 3.1 Induction of the AD-like mouse model using 3 nmol of MC903 and ovalbumin for 7 days

The dermatitis group exhibited the highest dermatitis score (Supplementary Figure S1). Among the three groups treated with Runfuzhiyang powder, the medium-concentration group showed the most effective treatment results (Supplementary Figure S1). Observing the number of scratches over 30 min revealed a decrease in the treatment group, with the medium-concentration group displaying the most significant reduction in scratching behavior (Supplementary Figure S2).

### 3.2 Dorsal skin thickness and eosinophil accumulation under the influence of Runfuzhiyang powder on skin lesions of AD mice

H&E staining revealed that the dorsal skin of the dermatitis group was significantly thicker compared to the control group. The groups treated with low, medium, and high doses of Runfuzhiyang powder showed significantly reduced dorsal skin thickness compared to the dermatitis group. The medium-dose group exhibited the most significant reduction in dorsal skin thickness and was most similar to the positive control group. Additionally, the dermatitis group had a significantly higher number of eosinophils than the control group. In contrast, the low-, medium-, and high-dose groups had significantly fewer eosinophils compared to the dermatitis group, with the high-dose group showing eosinophil counts closest to those in the positive control group (Supplementary Figure S3; Supplementary Figure S4).

### 3.3 Effects of Runfuzhiyang powder on mast cell infiltration in AD mice

Toluidine blue staining results showed that the dermatitis group had a significantly higher number of mast cells compared to the control group. In contrast, the high- and medium-dose groups, as well as the positive control group, exhibited significantly fewer mast cells than the dermatitis group (Supplementary Figure S5; Supplementary Figure S6).

### 3.4 Analysis of serum IgE levels

To elucidate the molecular effects of different doses of Runfuzhiyang powder on AD, serum IgE levels were measured in each group. Compared to the control group, the dermatitis group exhibited significantly higher IgE levels. In contrast, the low-, medium-, and high-dose groups had significantly lower IgE levels than the dermatitis group. The medium-dose group showed the lowest IgE levels and the mildest inflammation. Therefore, it is suggested that selecting an appropriate dose of Runfuzhiyang powder based on its therapeutic effect can effectively treat AD (Supplementary Figure S7).

Considering scratching behavior, dorsal skin thickness, eosinophil accumulation, mast cell infiltration, and IgE levels together, the medium-dose *Runfuzhiyang* treatment demonstrated the most effective outcome on AD mice. Consequently, the medium-dose Runfuzhiyang treatment group was selected for follow-up sampling and sequencing analysis.

### 3.5 Differential analysis

**FIGURE 2:**
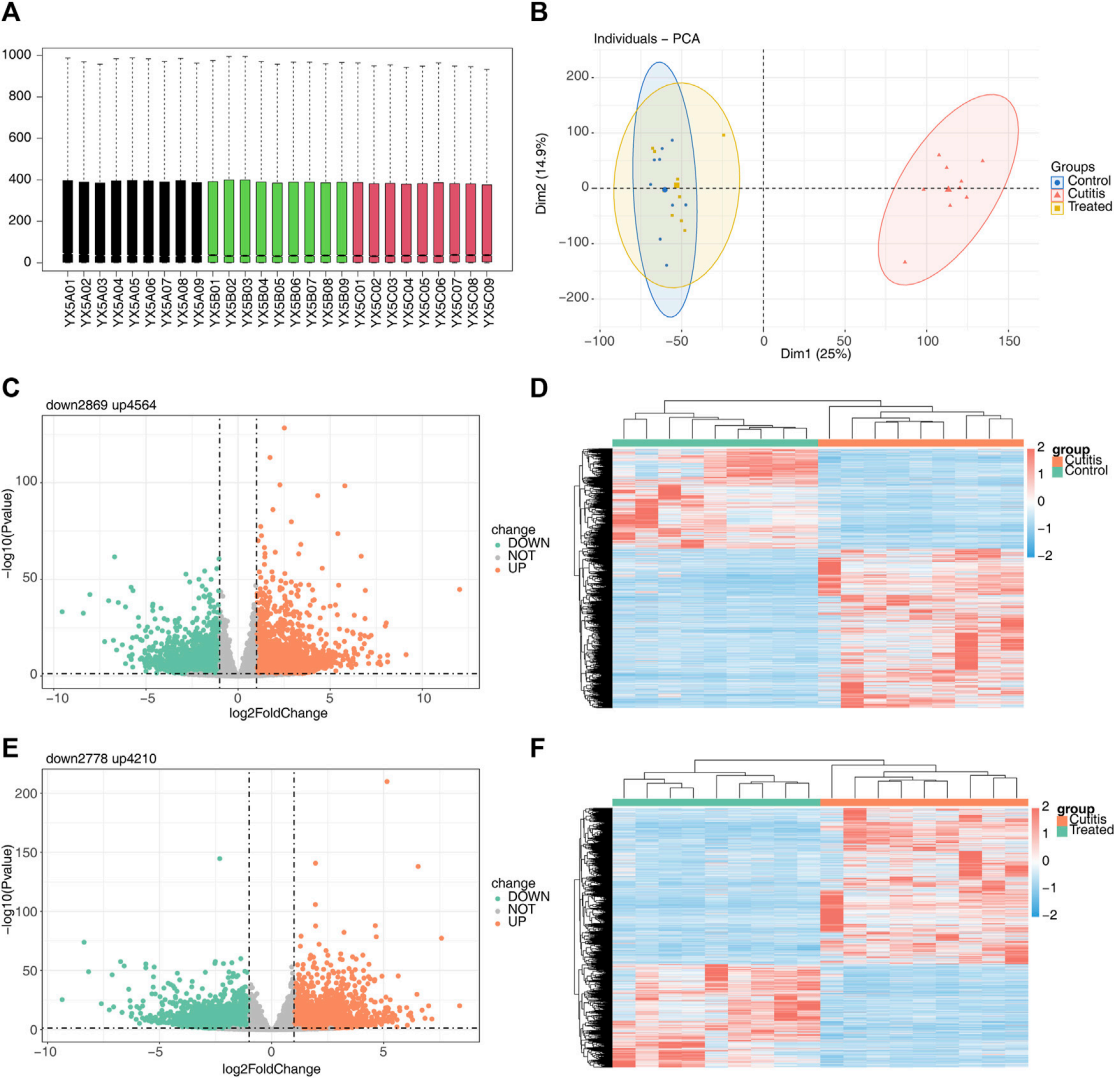
Differential expression analysis among different groups. **(A)** Box plots of the gene expression matrix of 27 samples. **(B)** Principal component analysis (PCA) of control, cutitis, and treatment groups. **(C, D)** Volcano map **(C)** and heatmap **(D)** of differentially expressed genes (DEGs) between cutitis and control groups. **(E, F)** Volcano map **(E)** and heatmap **(F)** of DEGs between cutitis and treatment groups.

The original sequencing data were preprocessed and filtered to obtain clean data. The filtered sequencing data were then aligned with the reference genome of the mouse, showing that the sequence alignment rate in all 27 sequenced samples was higher than 73% (Supplementary Table S1). Consistent expression across all 27 samples (Figure 2A) validated their use for subsequent expression analysis. PCA revealed that the control and treatment groups exhibited similar expression patterns, while the cutitis group demonstrated a significantly different expression pattern compared to the control and treatment groups (Figure 2B).

The cutitis group exhibited a total of 7,433 DEGs, with 4,564 upregulated and 2,869 downregulated DEGs, compared to the control group (Figures 2C,D). Additionally, 6,988 DEGs were identified in the cutitis group compared to the treatment group, with 4,210 upregulated and 2,778 downregulated (Figures 2E,F).

### 3.6 Key module genes related to AD

**FIGURE 3:**
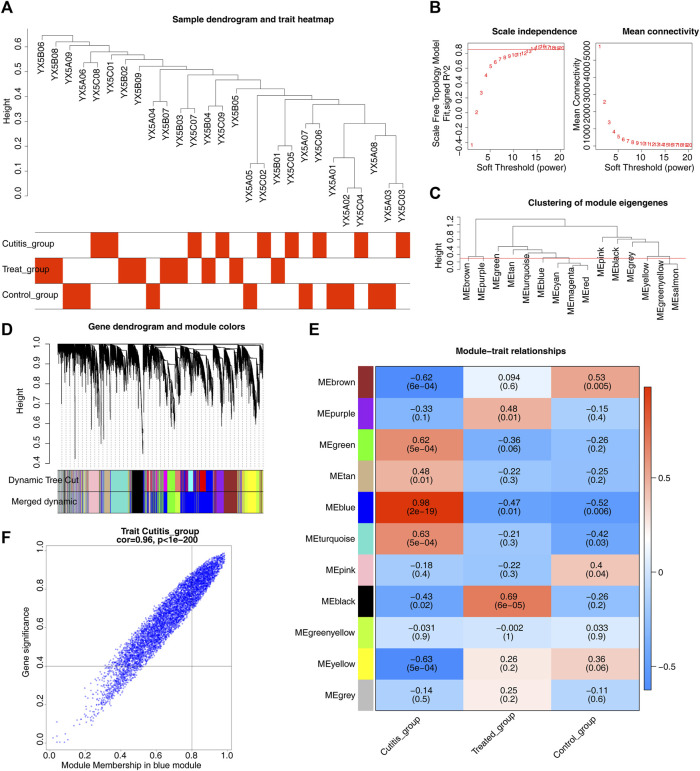
Identification of key module genes related to atopic dermatitis (AD). **(A)** Heatmap of sample clustering and traits. **(B)** Selection of the optimal soft-thresholding (power). **(C)** Cluster dendrogram of samples. **(D)** Hierarchical clustering of genes and module identification. **(E)** Heatmap of the relationships between gene modules and differential immune cells. **(F)** Scatterplot of 498 genes in the blue module.

WGCNA was conducted to identify genes associated with AD. Sample clustering results are shown in Figure 3A. A scale-free network was constructed using a soft threshold (β) of 14 (scale-free R2 = 0.85) (Figure 3B). A cluster dendrogram was then obtained (Figure 3C). A total of 11 modules were identified using the dynamic tree cutting algorithm with MEDissThres set to 0.3 (Figure 3D). Subsequent correlation analysis between the modules and the cutitis, control, and treatment groups highlighted the blue module as the key module (Figure 3E). Ultimately, 498 genes within the blue module were identified as key module genes (Figure 3F).

### 3.7 Screening and enrichment analysis of candidate genes

**FIGURE 4:**
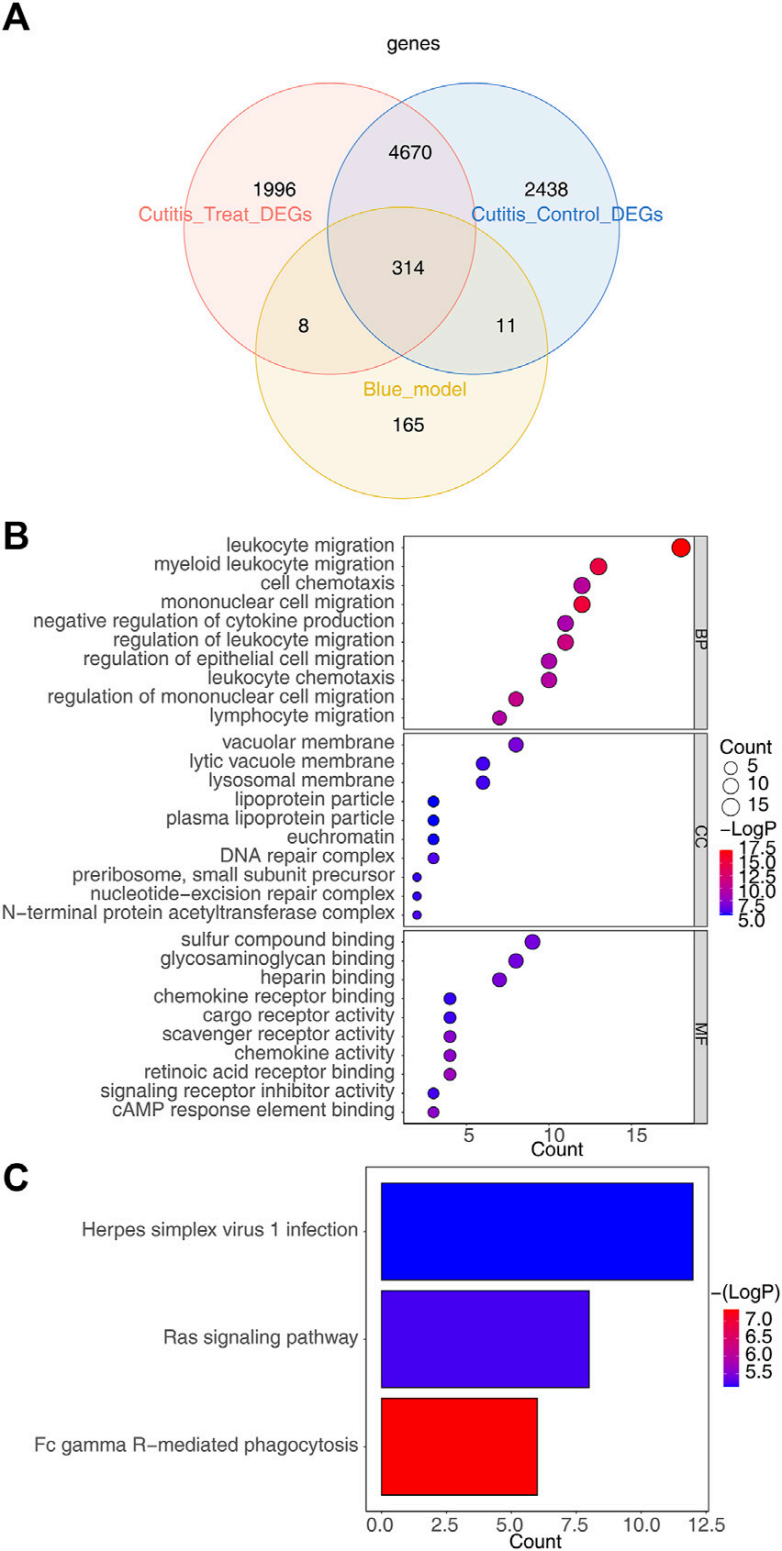
Identification of candidate genes and functional enrichment analysis. **(A)** Venn diagram of 314 candidate genes obtained by overlapping key module genes, DEGs between cutitis and control groups, and DEGs between cutitis and treatment groups. **(B, C)** Gene Ontology (GO) terms **(B)** and Kyoto Encyclopedia of Genes and Genomes (KEGG) pathways **(C)** enriched in candidate genes. BP, biological progress; CC, cellular component; MF, molecular function.

The intersection of 498 key module genes, 7,433 DEGs from the cutitis vs control comparison, and 6,988 DEGs from the cutitis vs treatment comparison identified 314 candidate genes, as depicted in the Venn diagram in Figure 4A. GO and KEGG analyses indicate that these 314 candidate genes are involved in biological processes such as cell chemotaxis, mononuclear cell migration, macrophage migration, positive regulation of inflammatory response, immune effector process regulation, Ras signaling pathway, Fc gamma receptor-mediated phagocytosis, and Herpes simplex virus 1 infection (Figures 4B,C and Supplementary Table S2).

### 3.8 Screening of biomarkers and validation

**FIGURE 5:**
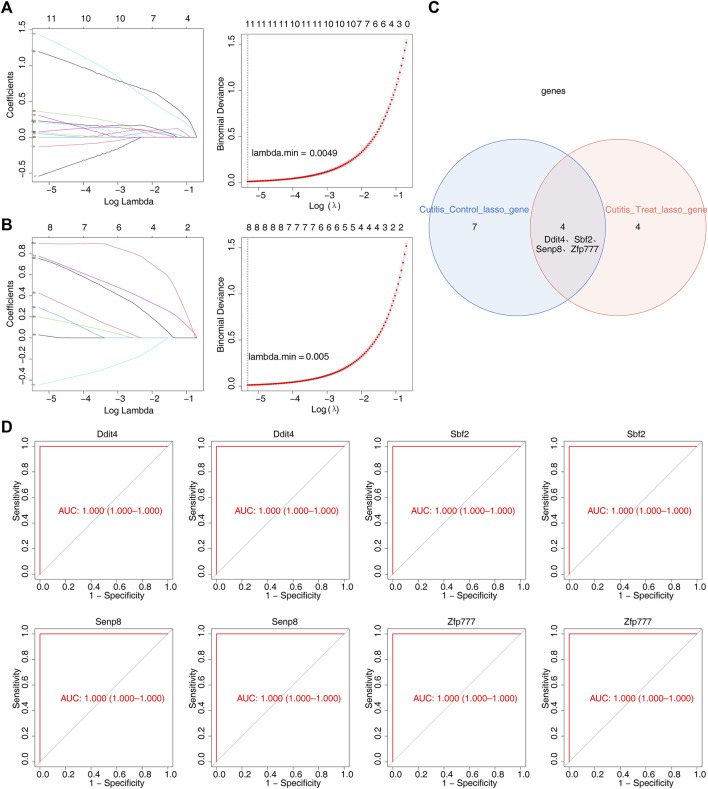
Identification of biomarkers. **(A, B)** Least absolute shrinkage and selection operator (LASSO) regression analysis of feature genes with a minimum lambda value between cutitis and control groups **(A)** and cutitis and treatment groups **(B)**. **(C)** Venn diagram of four biomarkers. **(D)** Receiver operating characteristic (ROC) curves of four biomarkers. AUC, area under the curve.

LASSO regression analysis was employed to further screen the 314 candidate genes. This analysis identified 11 feature genes (4933431E20Rik, *Ddit4*, *Jdp2*, *Mctp1*, *Sbf2*, *Senp8*, *Serpina3m*, *Slc10a6*, *Slc20a1*, *Slc25a28*, and *Zfp777*) that distinguished the cutitis group from the control group (Figure 5A). Additionally, eight feature genes (*Ddit4*, *Fam209*, *Gm9465*, *Sbf2*, *Senp8*, *Serpina3n*, *Xdh*, and *Zfp777*) distinguished the cutitis group from the treatment group (Figure 5B). The intersection of these feature genes identified four biomarkers (Ddit4, Sbf2, Senp8, and Zfp777) (Figure 5C). ROC curves demonstrated that these four biomarkers possess strong diagnostic capabilities across the cutitis, control, and treatment groups (Figure 5D).

### 3.9 GGI and GSEA

**FIGURE 6:**
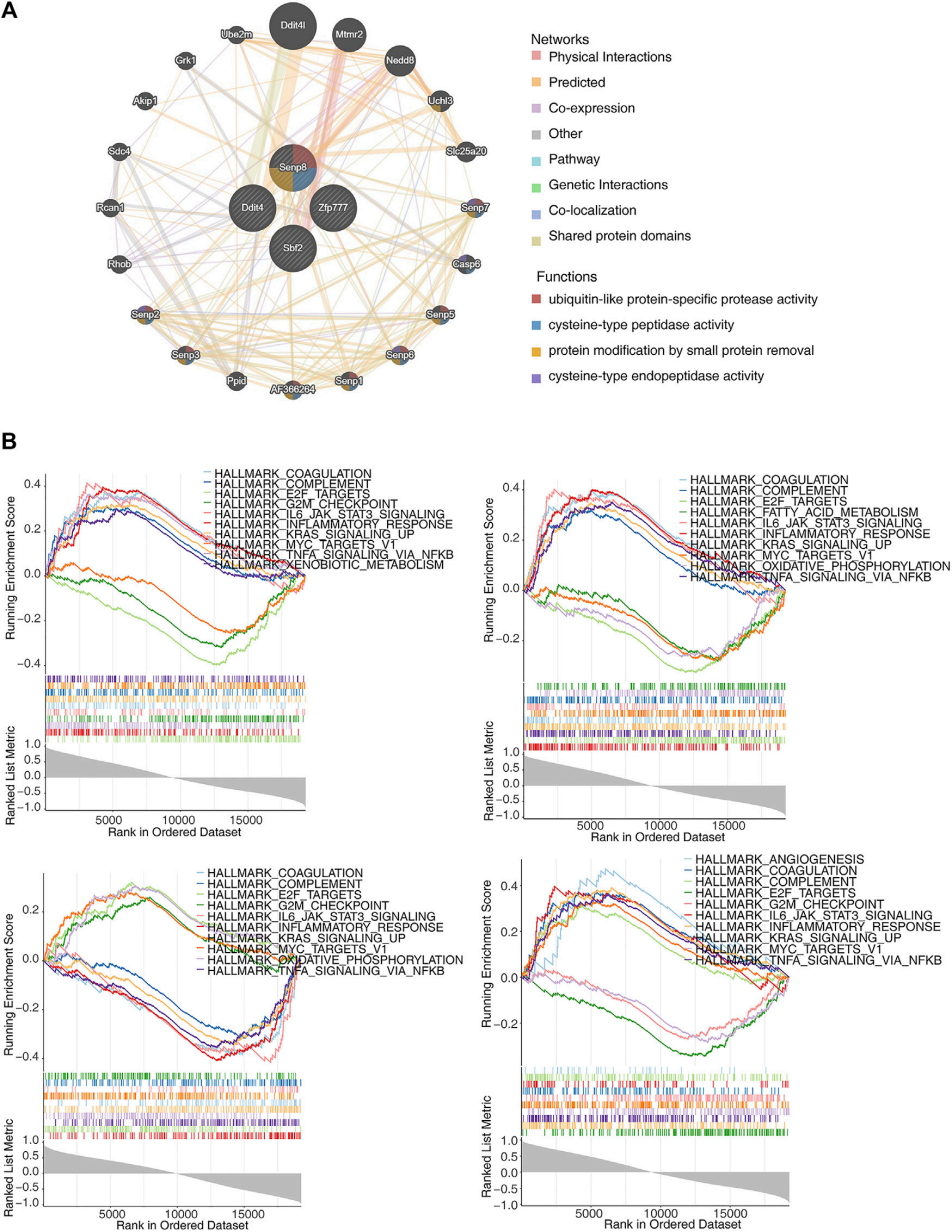
Exploration of the interactions and potential functions of biomarkers. **(A)** Protein–protein interaction (PPI) network of biomarkers. **(B)** Single-gene gene set enrichment analysis (ssGSEA) of biomarkers.

The GeneMANIA database was utilized to construct GGI networks for the four identified biomarkers to investigate their linkages (Figure 6A). The four biomarkers and the top 20 related genes were associated with cysteine-type peptidase activity, ubiquitin-like protein-specific protease activity, and cysteine-type endopeptidase activity. Subsequently, GSEA was performed to explore the signaling pathways. The results indicated that these pathways involved E2F targets, G2M checkpoints, KRAS signaling upregulation, and inflammatory responses, all associated with the four biomarkers (Figure 6B).

### 3.10 Immune infiltration and correlation analysis

**FIGURE 7:**
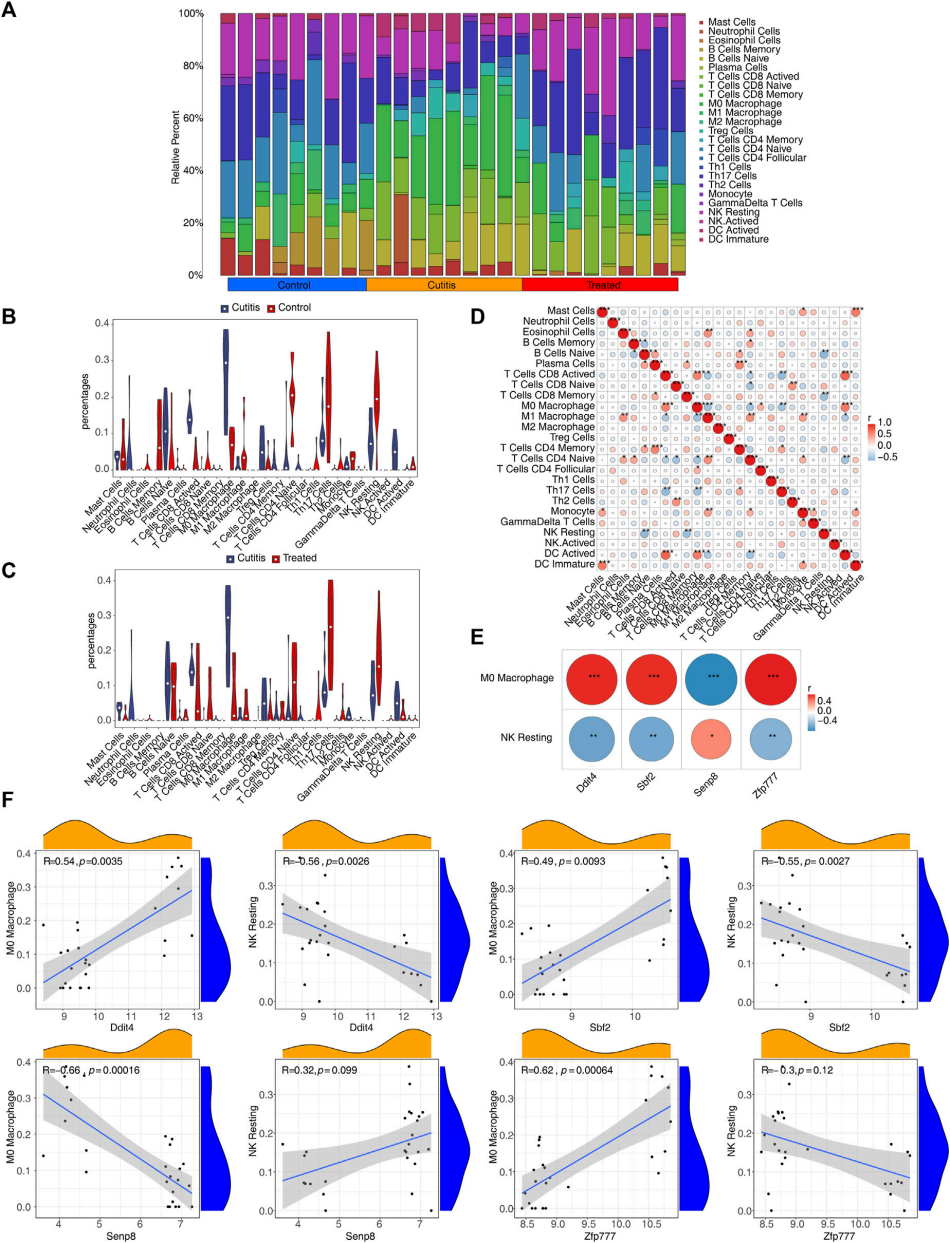
Immune infiltration analysis. **(A)** The proportion of 25 immune cells in samples. **(B,C)** Discrepancies of the percentages of immune cells between cutitis and control groups **(B)**, cutitis and treatment groups **(C)**. ns, not significant; *p < 0.05; **p < 0.01; ***p < 0.001. **(D)** The correlation analysis among immune cells. **(E)** The correlation of biomarkers to differential immune cells. **(F)** Scatterplot of biomarker correlation with differential immune cells.

The CIBERSORT algorithm was employed to calculate the proportions of 25 infiltrating immune cell types, aiming to analyze the differential immune cell types between the groups (Figure 7A). The analysis identified eight differential immune cells between the cutitis and control groups: plasma cells, naive B cells, memory B cells, activated CD8 T cells, M0 macrophages, naive CD4 T cells, resting NK cells, and activated dendritic cells (DCs) (Figure 7B). In contrast, the cutitis and treatment groups showed five differential immune cells: mast cells, M0 macrophages, Th17 cells, resting NK cells, and monocytes (Figure 7C).

The correlation analysis of immune cells indicated that M0 macrophages were positively correlated with activated DCs, follicular CD4 T cells, and activated CD8 T cells, while negatively correlated with Th17 cells, naive CD4 T cells, and M1 macrophages. Resting NK cells were negatively correlated with memory CD8 T cells and naive B cells (Figure 7D). Furthermore, Ddit4, Sbf2, and Zfp777 showed significant positive correlations with M0 macrophages and significant negative correlations with resting NK cells, whereas Senp8 exhibited the opposite correlations (Figure 7E; Supplementary Figure S8).

Immunofluorescence co-localization results demonstrated that Ddit4 co-localization with the marker CD56 of resting NK cells was significantly higher in the disease group compared to the control group and lower in the treatment group compared to the disease group (Supplementary Figure S9). Similarly, Ddit4 co-localization with the marker CD68 in M0 macrophages followed the same pattern (Supplementary Figure S10).

### 3.11 Regulatory network of biomarkers

**FIGURE 8:**
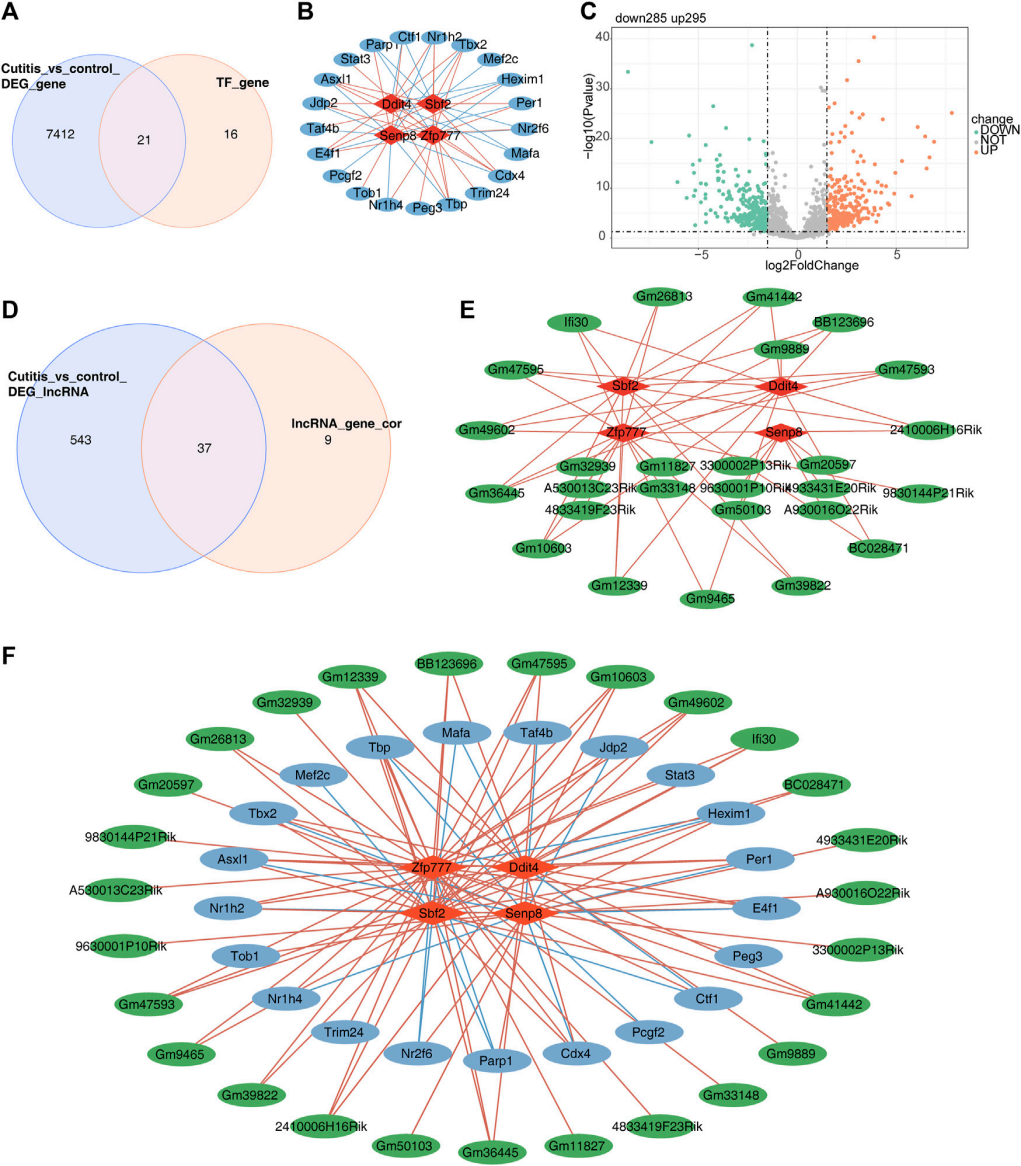
Construction of a regulatory network for biomarkers. **(A)** Venn diagram of 50 candidate transcription factors (TFs). **(B)** Network of TF-mRNA. Red graphics represent mRNAs, and blue graphics represent TFs. **(C)** Volcano map of differentially expressed long non-coding RNAs (DE-lncRNAs). **(D)** Venn diagram of 200 lncRNAs related to biomarkers. **(E)** Network of lncRNA–mRNA. Red graphics represent mRNAs, and green graphics represent lncRNAs. **(F)** Network of TF–mRNA–lncRNA. Red graphics represent mRNAs, green graphics represent lncRNAs, and blue graphics represent TFs.

First, 827 TFs were obtained from the TRRUST database. Analysis of TFs related to the four biomarkers yielded 37 TFs. The intersection of these 37 TFs with the DEGs from the cutitis group *versus* the control group identified 21 candidate TFs (Figure 8A). A TF–mRNA network containing 25 nodes and 56 edges is presented in Figure 8B. Additionally, 580 differentially expressed long non-coding RNAs (DE-lncRNAs) (295 upregulated and 285 downregulated) were identified in the cutitis group *versus* the control group (Figure 8C). Among these, 46 lncRNAs related to the four biomarkers were mined and 37 were selected by overlapping with the 580 DE-lncRNAs (Figure 8D). An lncRNA–mRNA network consisting of 31 nodes and 51 edges was constructed (Figure 8E). Finally, a comprehensive TF–mRNA–lncRNA network based on the four biomarkers, 21 candidate TFs, and 37 candidate lncRNAs was established, comprising 52 nodes and 107 edges (Figure 8F).

### 3.12 Drug–gene network analysis

**FIGURE 9:**
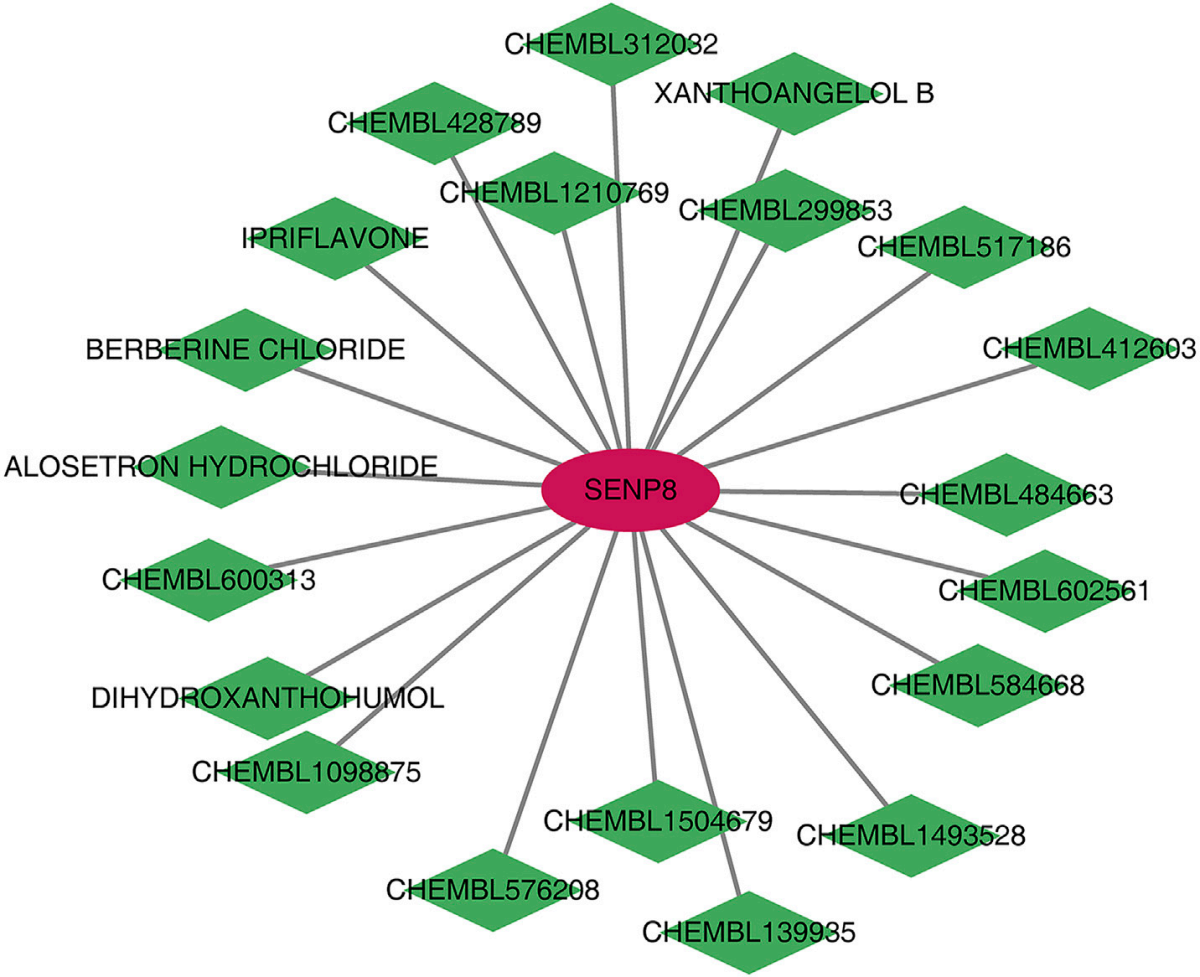
Potential drug prediction for biomarkers. Red graphics represent mRNAs, and green graphics represent drugs/compounds.

Human homologous genes corresponding to the four biomarkers were identified. Using the DGIDB database, 20 drugs/compounds targeting Senp8 were predicted, while no potential drugs were identified for the other biomarkers. A drug–gene network comprising 21 nodes and 21 edges was constructed (Figure 9), highlighting compounds such as CHEMBL1504679, CHEMBL584668, IPRIFLAVONE, and CHEMBL484663 as potential targets for SENP8.

### 3.13 Expression validation of biomarkers

**FIGURE 10:**
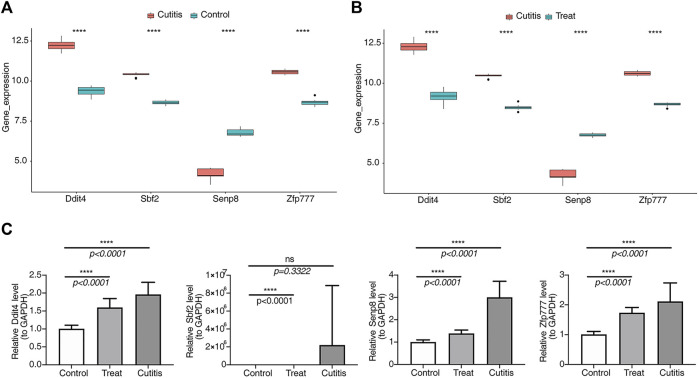
Analysis and validation of biomarker expression. **(A, B)** Expression of biomarkers in control, cutitis, and treatment groups. ****p< 0.0001. **(C)** Validation of biomarkers by reverse transcription–quantitative PCR (RT-qPCR).

As shown in Figures 10A,B, expressions of Ddit4, Sbf2, and Zfp777 were upregulated in the cutitis group compared to the control and treatment groups. Conversely, Senp8 expression was downregulated in the cutitis group relative to the control and treatment groups. Reverse transcription–quantitative PCR (RT-qPCR) further validated these findings, showing significantly higher expressions of Ddit4, Sbf2, and Zfp777 in the cutitis group (Figure 10C). However, Senp8 expression, due to sample heterogeneity, did not align with the transcriptome sequencing data.

## 4 Discussion

Variants of functional genes at most identified loci contributing to AD heritability remain unclear. However, these genomic regions are known to harbor multiple genes involved in various immune responses, including type 2 differentiation, T-cell activation, and innate immunity, as well as the epidermal differentiation complex, which is crucial for skin barrier function (Weidinger et al., 2018). The heritability gap may be due to additional unidentified loci or heritable epigenetic effects. Therefore, identifying new markers is essential for a better understanding of disease occurrence and developing appropriate treatment options.

In this study, expressions of Ddit4, Sbf2, and Zfp777 were upregulated, while Senp8 was downregulated in the cutitis group compared to the control and treatment groups. These four biomarkers are implicated in cysteine-type peptidase activity, ubiquitin-like protein-specific protease activity, and cysteine-type endopeptidase activity. They are also involved in the pathways of E2F targets, G2M checkpoint, KRAS signaling upregulation, and inflammatory response.

Ddit4 (also known as Dig2, RTP801, and regulated in development and DNA damage response 1 (REDD1)) is a highly conserved stress response gene upregulated by various cellular stressors, including hypoxia, DNA damage, energy stress, nutrient depletion, and endoplasmic reticulum stress. Notably, Ddit4 protects dividing cells from hypoxia or H_2_O_2_-induced apoptosis but sensitizes differentiated cells to stress. A small increase in Ddit4 is beneficial; however, chronic and sustained increases are detrimental to neurons (Canal et al., 2014). Ddit4 upregulation occurs during T-cell activation, and its absence reduces the proliferation of PHA-stimulated T cells and decreases the survival of stimulated and unstimulated T cells. However, it does not appear to significantly affect T-cell activation (Reuschel et al., 2015). Additionally, Ddit4 regulates Th17 cell differentiation (Zhang et al., 2018). SET binding factor 2 (SBF2, also known as MTMR13) functions as a membrane-associated complex regulating phosphoinositide (PI) levels and activating Rab GTPase, essential for vesicle transport and membrane trafficking. This regulation influences Schwann cell receptor trafficking through endosomes, and homozygous MTMR13 mutations have well-characterized pathological effects on human nerves (Mammel et al., 2022). Krüppel-associated box-containing zinc-finger proteins (KRAB-ZFPs) play crucial roles in cell proliferation, apoptosis, and differentiation, acting as potent transcriptional repressors. Zinc-finger protein 777 (ZFP777), a KRAB-ZFP family member, localizes to the nucleus and regulates cell proliferation depending on cell density (Yuki et al., 2015). SUMO peptidase family member, Nedd8-specific (SENP8), also known as Nedd8 protease 1 (NEDP1), primarily acts on non-Cullins Neddylation-modifying substrates and regulates various physiological processes, such as cell growth, cell cycle, oxidative stress, and DNA damage repair (Chan et al., 2008). Nedd8 plays a role in forming various ubiquitinated inclusions via the ubiquitin–proteasome system (Mori et al., 2005). SENP8 is essential for inflammatory activation of the NF–κB signaling pathway (Yan et al., 2017). Neddylation modifications affect immune cell functions, such as macrophages, by modulating CRL (Cullin-RING ligase) substrate activity, including TFs like NF–κB (Huang et al., 2015). In a mouse model with UBA3 (ubiquitin-like modifier activating enzyme 3) and Nedd8 specifically knocked out in T cells, Neddylation promoted CD4^+^ T-cell activation, expansion, and Th1 cell protective responses while ensuring CD4^+^ T-cell survival post-activation (Cheng et al., 2018). HIF-1α-induced oxidative stress-related inflammatory responses also partially depend on SENP8’s deneddylation function (Curtis et al., 2015). These findings suggest SENP8 as a potential therapeutic target for inflammatory diseases.

Precise regulation of cellular proliferation is essential for tissue homeostasis and development as its misregulation leads to diseases resulting from either excessive proliferation or cell loss. The E2F family, primarily known for its pivotal role in the cell cycle, also influences various cancer-related processes including angiogenesis, metabolic reprogramming, metastasis, and DNA repair. E2Fs can promote apoptosis or cell survival in response to genotoxic stress, depending on cell type and the source and dose of DNA damage (Castillo et al., 2015; Rennhack and Andrechek, 2020). Aberrant mitosis and mitotic catastrophe result from bypassing the G2-M checkpoint, leading to the accumulation of DNA damage and subsequent apoptosis (Barnum and O’Connell, 2014). G2/M cycle arrest can cause fibrosis in various organs, resulting in poor prognosis; thus, reversing G2/M cycle arrest could be a potential target for anti-fibrotic therapy (Oshima and Fujiu, 2019). RAS proteins act as binary switches, cycling between ON and OFF states during signal transduction. The prevalence of RAS mutations in human cancer has long been recognized, with KRAS being the most frequently mutated gene, accounting for 85% of all RAS-driven cancers (Simanshu et al., 2017). RAS-mutant cancers exhibit increased macropinocytosis and autophagy. In mouse models, ablation of RAS oncogenes results in dramatic tumor regression (Papke and Der, 2017).

AD, a chronic inflammatory disease with a heterogeneous profile, involves the type 2 immune pathway (Th2) in adults and Th17, Th22, and Th2 cells in children (Weidinger and Novak, 2016). Various immune cells, including dendritic cells, macrophages, neutrophils, NK cells, innate lymphoid cells, inflammatory epidermal dendritic cells (IDECs), basophils, and eosinophils, infiltrate AD lesions. Evaluating the infiltration of these immunocytes and identifying their diverse constituents are crucial for understanding the molecular mechanisms underlying AD.

Five types of differential immune cells—mast cells, M0 macrophages, Th17 cells, resting NK cells, and monocytes—were identified in both the cutitis and treatment groups. Ddit4, Sbf2, and Zfp777 showed significant positive correlations with M0 macrophages and significant negative correlations with resting NK cells. The role of mast cells in AD pathogenesis has been extensively studied. In mice, allergen-provoked acute itch is mediated by the mast cell–histamine axis during the steady state; however, AD-associated inflammation renders this pathway dispensable, and histamine-induced itch treatment in AD is largely ineffective (Fan and Mishra, 2022). Compared to AD patients of American or European descent, those of Asian descent exhibit more robust Th17 cell activation (Noda et al., 2015). The roles of macrophages, Th17 cells, and NK cells in AD are not fully understood. Macrophages, as tissue-resident or infiltrated immune cells, are crucial for host defense, tissue repair, and anti-inflammation, with their reprogramming being responsible for disease development or regression (Watanabe et al., 2019). Macrophages exhibit remarkable plasticity, sensing various endogenous or exogenous signals and responding rapidly (Mosser et al., 2021). They play roles in acne vulgaris (Do et al., 2022; Liu et al., 2023), hidradenitis suppurativa, scar formation (Smith et al., 2023), and vital organ damage in autoimmune diseases such as dermatomyositis (Huang et al., 2022), anti-MDA5 antibody-associated interstitial lung disease (Gono et al., 2022), and lupus nephritis (Valiente et al., 2022). Increased Mac2 levels have been observed in AD skin compared to healthy skin, with a decline in Mac2 and EASI after 12 weeks of methotrexate treatment (Reynolds et al., 2021). Significant differences were noted in local macrophage infiltration before and after Runfuzhiyang powder treatment. Immunofluorescence co-localization showed higher co-localization of Ddit4 with the marker CD68 in M0 macrophages in the disease group compared to the control group, which decreased after Runfuzhiyang powder treatment. Further investigation is needed to determine if AD pathogenesis is related to macrophage proliferation and polarization caused by key genes or the pathways through which Runfuzhiyang powder affects macrophage polarization. NK cells produce cytokines and chemokines such as IFN-γ, TNF-α, and IL10, serving an immunoregulatory function that influences both innate and adaptive immune systems (Cooper et al., 2001). Full activation of resting NK cells requires co-engagement of distinct activating receptors (Bryceson et al., 2006; 2009). NK cell-mediated immunomodulation may play a role in immune dysregulation observed in AD. Enrichment of lesional skin with activated NK cells could be a counter-regulatory response to type 2 skin inflammation (Mack et al., 2020). The co-localization of Ddit4 with the marker CD56 of resting NK cells was significantly higher in the disease group compared to the control group and decreased after Runfuzhiyang powder treatment. Notably, the full functions of NK T cells and macrophages in the AD immune system remain to be elucidated.

Rather than being a stable entity with well-defined components, the skin immune system comprises a network of highly flexible cellular players capable of adjusting their functions, according to environmental needs and challenges. This study is the first to explore the diagnostic value of Ddit4, Sbf2, Zfp777, and Senp8 in AD and their relationship with skin immune infiltrating cells. The findings suggest that Runfuzhiyang powder may treat AD by regulating the proliferation, activation, and apoptosis of immune cells such as macrophages, NK cells, and Th cells through modulation of these key genes, thereby altering the inflammatory process. Additionally, pathways related to metabolism, oxidative stress, and ubiquitination might also play a role. These hypotheses warrant further investigation to elucidate the intricate interplay between genes and immunocytes.

Despite the promising findings, this study has several limitations. First, the expression and role of the genes in AD at the protein level require further investigation. Second, the candidate genes identified in our study were based on the MC903-induced AD model. Although topical MC903 induces AD-like inflammatory phenotypes that closely resemble human AD, there remains a gap between the model and the human condition due to AD’s heterogeneity. Therefore, the diagnostic value of the identified signatures is limited. Finally, all mechanistic analyses in our study were descriptive. To better understand and treat AD, further research is necessary, such as examining the differences in protein expression levels of these biomarkers (e.g., via Western blot) to explore the distinctions between the disease and controls. Additionally, constructing transgenic mice through gene editing will help further investigate the specific mechanisms of these biomarkers in AD.

## Data Availability

The datasets analyzed for this study can be found in the (Ensembl repository) (https://www.ensembl.org/Mus_musculus/Info/Index?redirect=no) and (Transcriptional regulatory relationships repository) (http://www.grnpedia.org/trrust).
