# Supplementary material for: Authentication and validation of key genes in the treatment of atopic dermatitis with Runfuzhiyang powder: combined RNA-seq, bioinformatics analysis, and experimental research
Source: Front Genet. 2024 Aug 1;15:1335093. doi: 10.3389/fgene.2024.1335093 (PMC11324508; doi:10.3389/fgene.2024.1335093)
Supplement: Supplementary file 1 [file Table1.DOCX]

Link of raw data

<https://www.jianguoyun.com/p/DVJEZmgQ8veNDBjJ9KUFIAA>
