# Supplementary material for: Authentication and validation of key genes in the treatment of atopic dermatitis with Runfuzhiyang powder: combined RNA-seq, bioinformatics analysis, and experimental research
Source: Front Genet. 2024 Aug 1;15:1335093. doi: 10.3389/fgene.2024.1335093 (PMC11324508; doi:10.3389/fgene.2024.1335093)
Supplement: Supplementary file 2 [file Table2.DOCX]

Supplementary Material

# Supplementary Figures

**
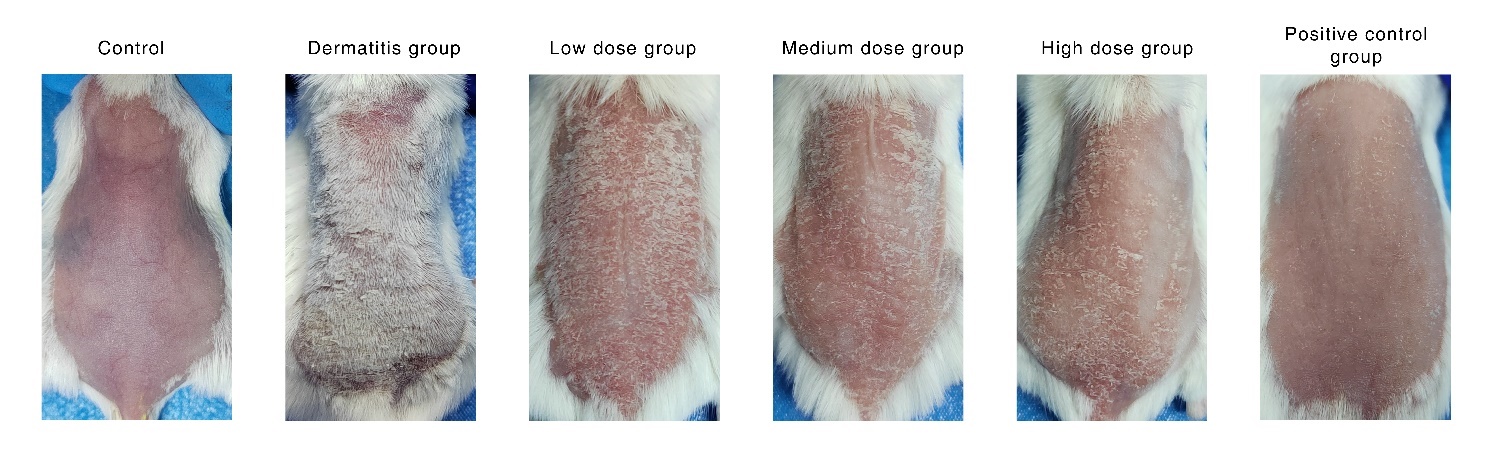
**

**Supplementary Figure 1** Presentation of dorsal skin of 6 groups of mice after different treatments, including control group (n=10), dermatitis group (n=10), low dose subgroup (n=10), medium dose subgroup (n=10), high dose group (n=10), and positive control group (n=4).

**
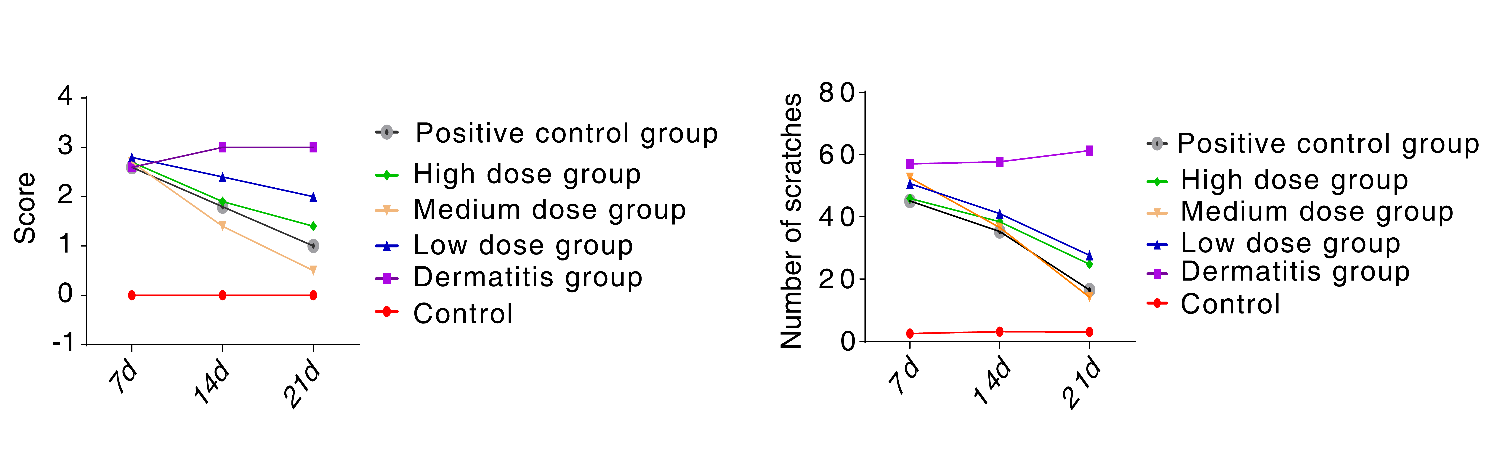
**

**Supplementary Figure 2** The discrepancies of dermatitis score and number of scratches in 30 minutes. Control group (n=10), dermatitis group (n=10), low dose subgroup (n=10), medium dose subgroup (n=10), high dose group (n=10), and positive control group (n=4).

**
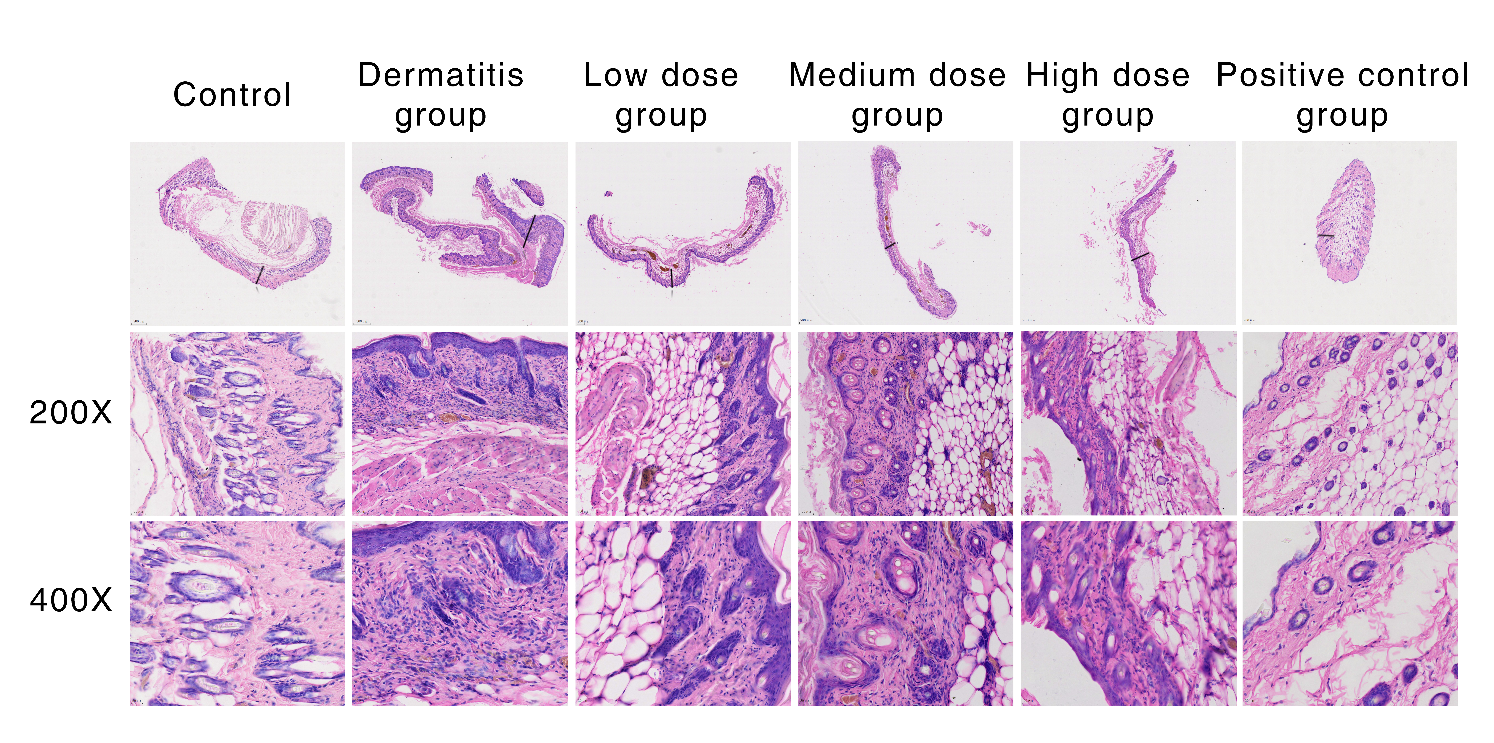
**

**Supplementary Figure 3** The results of hematoxylin-eosin (H&E) staining. Scale bar (1×), 500μm; Scale bar (200×), 50μm; Scale bar (400×), 20μm. Control group (n=10), dermatitis group (n=10), low dose subgroup (n=10), medium dose subgroup (n=10), high dose group (n=10), and positive control group (n=4). Choose no less than 5 fields of view in the middle and around for photo.

**
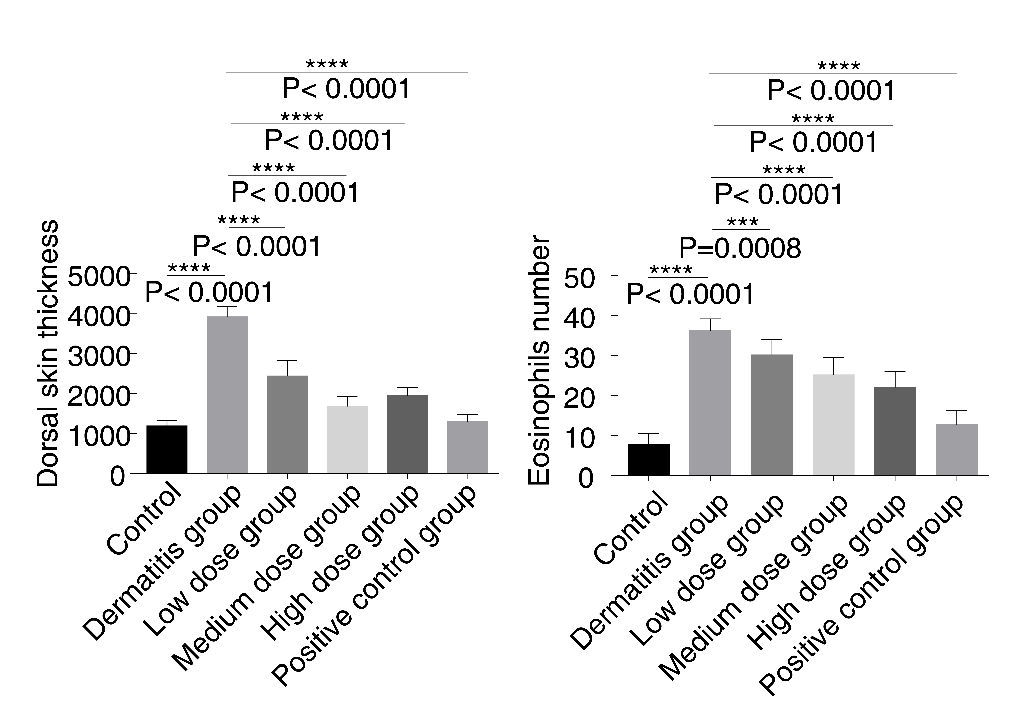
**

**Supplementary Figure 4** Comparison of dorsal skin thickness and the number of eosinophils between different groups. Control group (n=10), dermatitis group (n=10), low dose subgroup (n=10), medium dose subgroup (n=10), high dose group (n=10), and positive control group (n=4). One-way ANOVA test, *** P < 0.001, **** P < 0.0001.

**
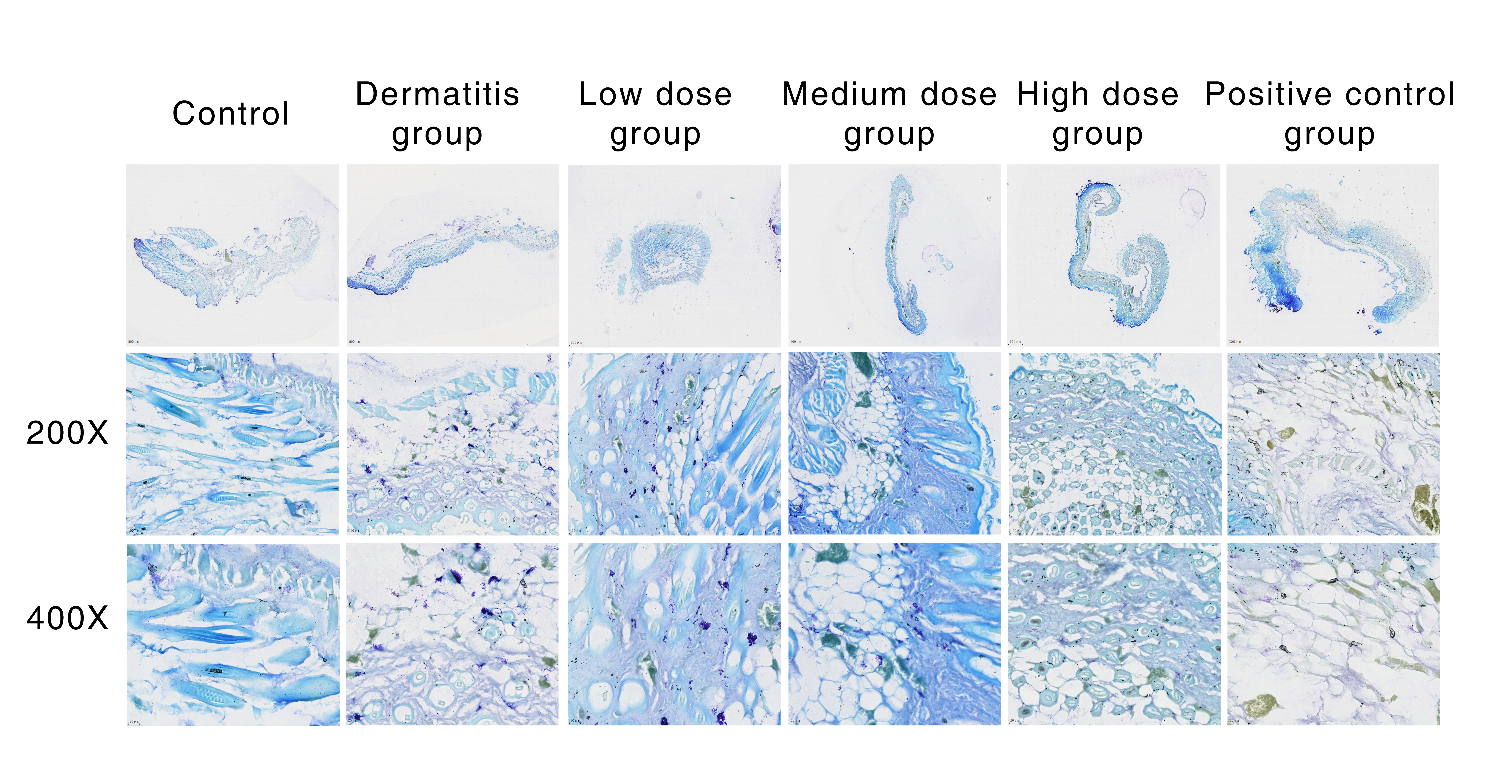
**

**Supplementary Figure 5** The results of toluidine blue staining. Scale bar (1×), 500μm; Scale bar (200×), 50μm; Scale bar (400×), 20μm. Control group (n=10), dermatitis group (n=10), low dose subgroup (n=10), medium dose subgroup (n=10), high dose group (n=10), and positive control group (n=4). Choose no less than 5 fields of view in the middle and around for photo.

**
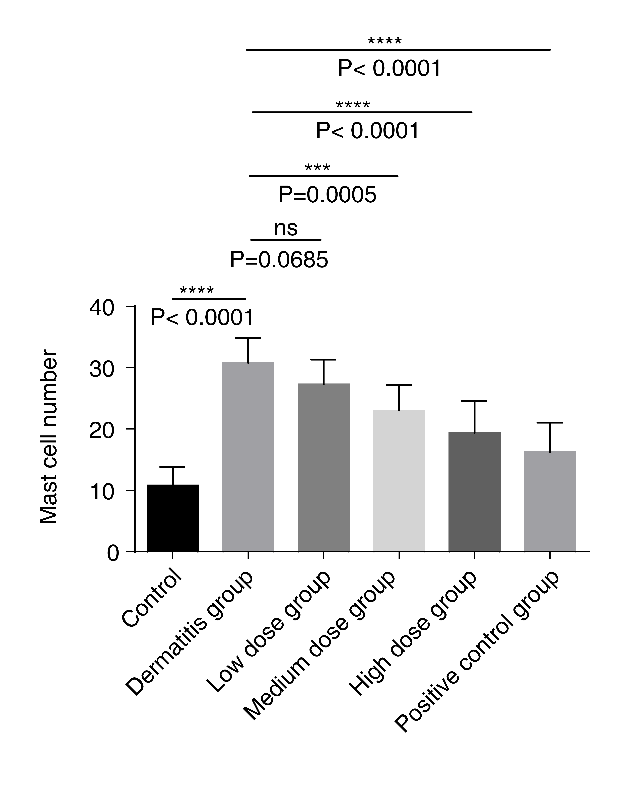
**

**Supplementary Figure 6** Comparison of the number of mast cells between different groups. One-way ANOVA test, *** P < 0.001, **** P < 0.0001.


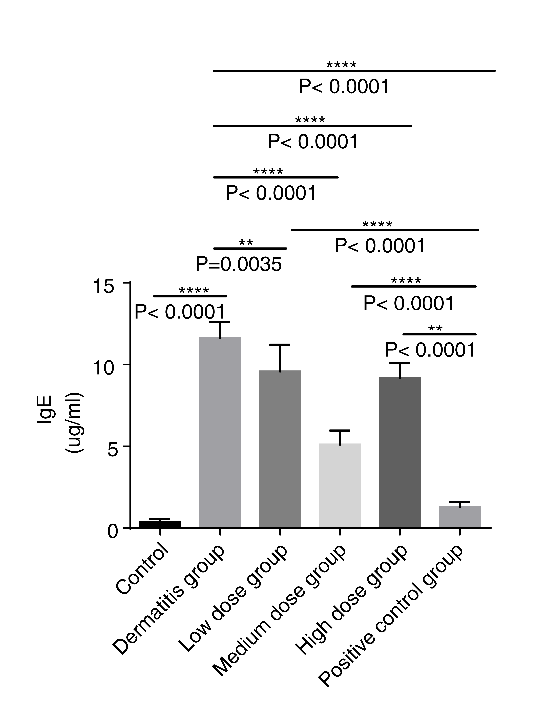


**Supplementary Figure 7** Comparison of the IgE levels between different groups. One-way ANOVA test, ** P < 0.01, *** P < 0.001, **** P < 0.0001.


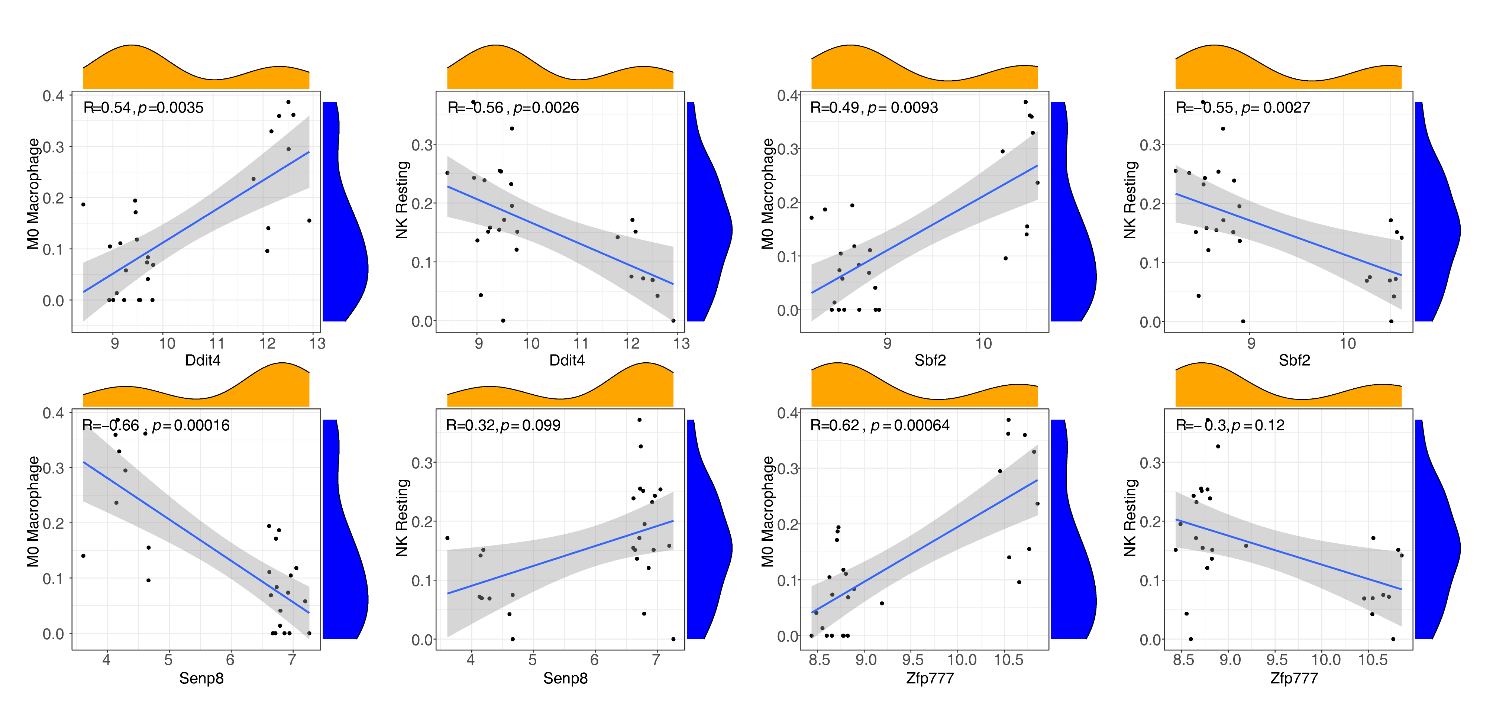


**Supplementary Figure 8** Spearman’s correlation between the biomarkers and immune cells.

**
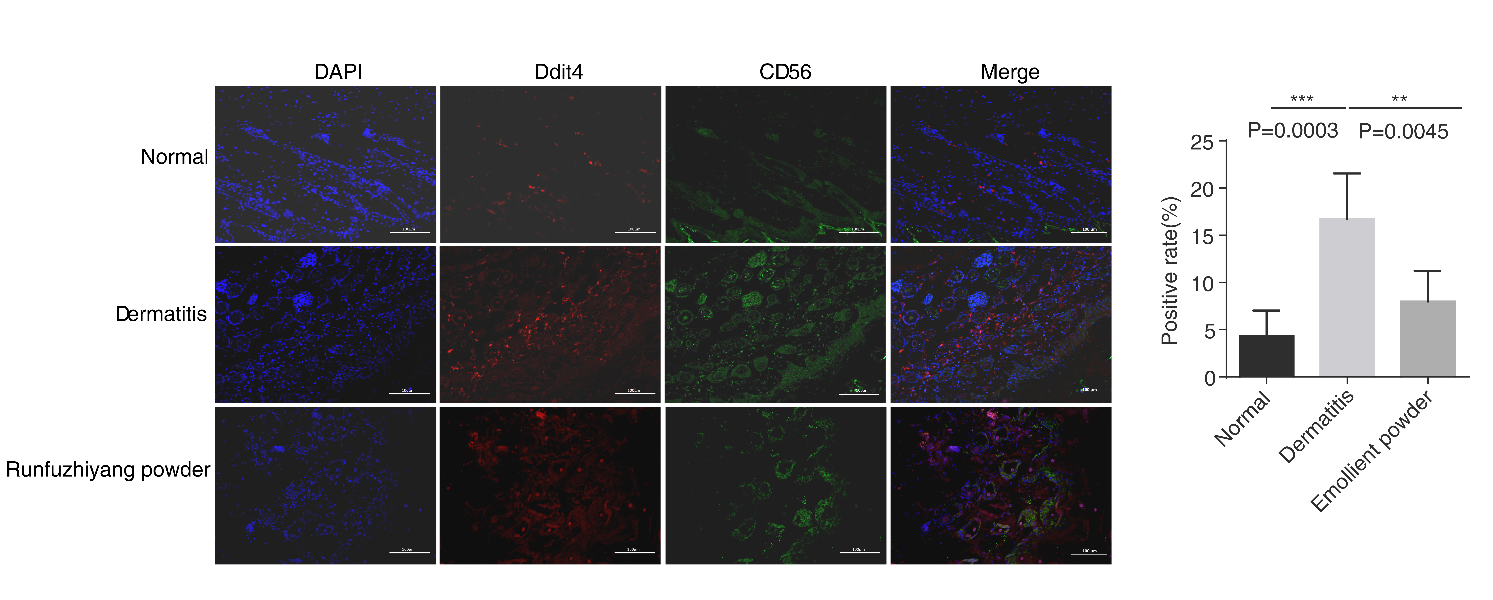
**

**Supplementary Figure 9** Immunofluorescence co-localization analysis of Ddit4 and CD56 in normal, dermatitis, and *Runfuzhiyang* powder groups. normal (n=6), dermatitis (n=6), and *Runfuzhiyang* powder groups (n=6). The positive rate was calculated by selecting 5 visual fields. One-way ANOVA test, ** P < 0.01, *** P < 0.001.

**
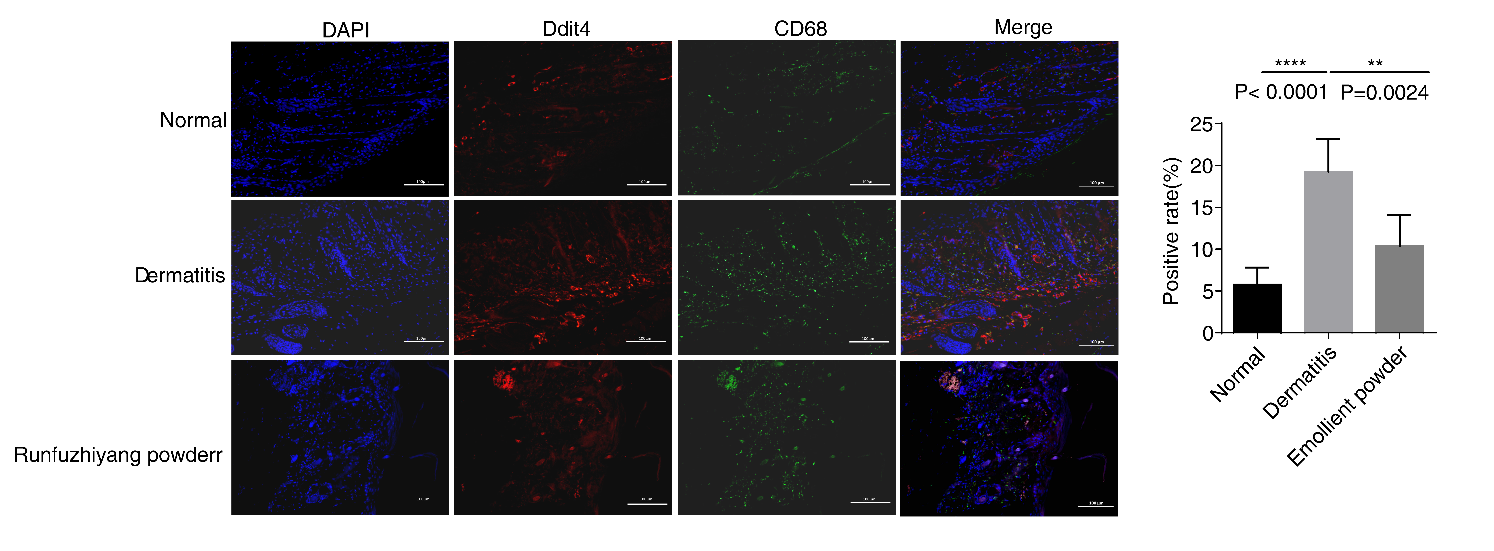
**

**Supplementary Figure 10** Immunofluorescence co-localization analysis of Ddit4 and CD68 in normal, dermatitis, and *Runfuzhiyang* powder groups. normal (n=6), dermatitis (n=6), and *Runfuzhiyang* powder groups (n=6). The positive rate was calculated by selecting 8 visual fields. One-way ANOVA test, ** P < 0.01, **** P < 0.0001.

**Supplementary Table 1** The results of sequence alignment for 27 samples.

| **Item** | **Number of input reads** | **Average input read length** | **Uniquely mapped reads number** | **Uniquely mapped reads %** | **Average mapped length** |
| --- | --- | --- | --- | --- | --- |
| YX5A01 | 20660953 | 296 | 18790436 | 90.95% | 295.21 |
| YX5A02 | 14717572 | 297 | 13363462 | 90.80% | 295.96 |
| YX5A03 | 13660649 | 297 | 12219739 | 89.45% | 295.71 |
| YX5A04 | 16053782 | 297 | 14447511 | 89.99% | 295.92 |
| YX5A05 | 16884943 | 297 | 15230811 | 90.20% | 296.05 |
| YX5A06 | 21222253 | 296 | 18996268 | 89.51% | 295.25 |
| YX5A07 | 20254625 | 296 | 18036185 | 89.05% | 294.71 |
| YX5A08 | 21792375 | 296 | 19638398 | 90.12% | 295.03 |
| YX5A09 | 15663602 | 297 | 13534332 | 86.41% | 296.21 |
| YX5B01 | 20835962 | 296 | 18645397 | 89.49% | 294.83 |
| YX5B02 | 21641913 | 296 | 19248755 | 88.94% | 294.91 |
| YX5B03 | 20865191 | 296 | 18776213 | 89.99% | 294.72 |
| YX5B04 | 20709994 | 296 | 18197498 | 87.87% | 295.38 |
| YX5B05 | 60477084 | 296 | 49738793 | 82.24% | 295.52 |
| YX5B06 | 16244915 | 296 | 13892070 | 85.52% | 295.46 |
| YX5B07 | 17620138 | 296 | 14864188 | 84.36% | 295.48 |
| YX5B08 | 15552383 | 297 | 13351979 | 85.85% | 295.9 |
| YX5B09 | 12861242 | 297 | 10798549 | 83.96% | 295.59 |
| YX5C01 | 17116473 | 296 | 14530647 | 84.89% | 295.49 |
| YX5C02 | 18776198 | 296 | 15142381 | 80.65% | 295.66 |
| YX5C03 | 16929711 | 296 | 13656375 | 80.67% | 295.09 |
| YX5C04 | 16978511 | 296 | 12475971 | 73.48% | 294.41 |
| YX5C05 | 19557523 | 295 | 15350844 | 78.49% | 294.24 |
| YX5C06 | 20634007 | 296 | 17271170 | 83.70% | 295.59 |
| YX5C07 | 18358752 | 295 | 14534042 | 79.17% | 294.21 |
| YX5C08 | 12702562 | 295 | 10247584 | 80.67% | 293.76 |
| YX5C09 | 52319153 | 294 | 41335086 | 79.01% | 292.87 |

**Supplementary Table 2** GO terms enriched in 314 candidate genes.

|  | ONTOLOGY | ID | Description | GeneRatio | BgRatio | pvalue | p.adjust | qvalue | geneID | Count |
| --- | --- | --- | --- | --- | --- | --- | --- | --- | --- | --- |
| GO:0050900 | BP | GO:0050900 | leukocyte migration | 18/276 | 373/29008 | 2.31E-08 | 7.48E-05 | 6.61E-05 | Ager/Akt1/Apod/Ccl6/Ccl9/Cd200r1/Crtam/Cxcl1/Fcgr3/Msn/Myo9b/Ninj1/P2ry12/Pf4/Ptpn22/Rarres2/Serpine1/Vegfd | 18 |
| GO:0097529 | BP | GO:0097529 | myeloid leukocyte migration | 13/276 | 226/29008 | 2.96E-07 | 0.000318956 | 0.000281896 | Ager/Ccl6/Ccl9/Cd200r1/Cxcl1/Fcgr3/Myo9b/Ninj1/P2ry12/Pf4/Rarres2/Serpine1/Vegfd | 13 |
| GO:0001667 | BP | GO:0001667 | ameboidal-type cell migration | 13/276 | 444/29008 | 0.000366222 | 0.046121376 | 0.040762403 | Ager/Akt1/Fgfr1/Gab2/Hmox1/Lcn2/Map4k4/P2ry12/Rhoj/Ric8a/Sema3a/Sema6b/Serpine1 | 13 |
| GO:0071674 | BP | GO:0071674 | mononuclear cell migration | 12/276 | 181/29008 | 1.85E-07 | 0.000299926 | 0.000265076 | Ager/Akt1/Apod/Ccl6/Ccl9/Cd200r1/Crtam/Msn/Myo9b/Ninj1/Rarres2/Serpine1 | 12 |
| GO:0060326 | BP | GO:0060326 | cell chemotaxis | 12/276 | 312/29008 | 4.98E-05 | 0.026867724 | 0.023745887 | Ccl6/Ccl9/Cxcl1/Fcgr3/Fgfr1/Lox/Myo9b/Ninj1/Pf4/Rarres2/Serpine1/Vegfd | 12 |
| GO:0042692 | BP | GO:0042692 | muscle cell differentiation | 12/276 | 428/29008 | 0.000890991 | 0.080090235 | 0.070784324 | Adamts15/Adamts5/Adm/Akt1/Cby1/Fer1l5/Foxf1/Lox/Ninj1/Tbx2/Tnnt2/Wfikkn1 | 12 |
| GO:0002685 | BP | GO:0002685 | regulation of leukocyte migration | 11/276 | 223/29008 | 1.06E-05 | 0.008568784 | 0.007573153 | Ager/Akt1/Apod/Cd200r1/Msn/Ninj1/P2ry12/Ptpn22/Rarres2/Serpine1/Vegfd | 11 |
| GO:0001818 | BP | GO:0001818 | negative regulation of cytokine production | 11/276 | 292/29008 | 0.000122774 | 0.036567108 | 0.032318272 | Ager/Apod/Cd200r1/Chid1/Fgfr1/Hmox1/Inpp5d/N4bp1/Nr1h4/Prg4/Ptpn22 | 11 |
| GO:0090130 | BP | GO:0090130 | tissue migration | 11/276 | 310/29008 | 0.000206409 | 0.043443702 | 0.038395856 | Ager/Akt1/Fgfr1/Foxf1/Gab2/Hmox1/Lcn2/Map4k4/Rhoj/Sema3a/Serpine1 | 11 |
| GO:0032103 | BP | GO:0032103 | positive regulation of response to external stimulus | 11/276 | 427/29008 | 0.002787029 | 0.136990007 | 0.121072751 | Ager/Crtam/Cxcl1/Fcgr3/Fgfr1/Ninj1/P2ry12/Pvr/Rarres2/Serpine1/Vegfd | 11 |
| GO:0048732 | BP | GO:0048732 | gland development | 11/276 | 477/29008 | 0.006327936 | 0.1491879 | 0.131853335 | Asxl1/Fgfr1/Foxf1/Hmga2/Hmox1/Hp/Msn/Plaur/Sema3a/Tbx2/Xdh | 11 |
| GO:0030595 | BP | GO:0030595 | leukocyte chemotaxis | 10/276 | 226/29008 | 6.68E-05 | 0.03068009 | 0.027115283 | Ccl6/Ccl9/Cxcl1/Fcgr3/Myo9b/Ninj1/Pf4/Rarres2/Serpine1/Vegfd | 10 |
| GO:0010632 | BP | GO:0010632 | regulation of epithelial cell migration | 10/276 | 241/29008 | 0.000113645 | 0.036567108 | 0.032318272 | Ager/Akt1/Fgfr1/Gab2/Hmox1/Lcn2/Map4k4/Rhoj/Sema3a/Serpine1 | 10 |
| GO:0010631 | BP | GO:0010631 | epithelial cell migration | 10/276 | 306/29008 | 0.000753554 | 0.073933008 | 0.065342522 | Ager/Akt1/Fgfr1/Gab2/Hmox1/Lcn2/Map4k4/Rhoj/Sema3a/Serpine1 | 10 |
| GO:0090132 | BP | GO:0090132 | epithelium migration | 10/276 | 308/29008 | 0.000791908 | 0.075371049 | 0.066613474 | Ager/Akt1/Fgfr1/Gab2/Hmox1/Lcn2/Map4k4/Rhoj/Sema3a/Serpine1 | 10 |
| GO:0040013 | BP | GO:0040013 | negative regulation of locomotion | 10/276 | 341/29008 | 0.00169457 | 0.114242269 | 0.100968137 | Adarb1/Ager/Akt1/Apod/Cd200r1/Chrd/Mctp1/Sema3a/Sema6b/Serpine1 | 10 |
| GO:0050727 | BP | GO:0050727 | regulation of inflammatory response | 10/276 | 342/29008 | 0.001731327 | 0.11433825 | 0.101052965 | Ager/Cd200r1/Ctla2a/Fcgr3/Fgfr1/Foxf1/Ninj1/Nr1h4/Sbno2/Serpine1 | 10 |
| GO:0045861 | BP | GO:0045861 | negative regulation of proteolysis | 10/276 | 358/29008 | 0.002412644 | 0.132327381 | 0.116951888 | Akt1/Ctla2a/N4bp1/Nr1h2/Plaur/Prkcg/Serpina3m/Serpina3n/Serpine1/Wfikkn1 | 10 |
| GO:0019221 | BP | GO:0019221 | cytokine-mediated signaling pathway | 10/276 | 397/29008 | 0.004991926 | 0.146677122 | 0.129634292 | Akt1/Ccl6/Ccl9/Cxcl1/Ifi27/Ifitm2/Nr1h4/Numbl/Osmr/Pf4 | 10 |
| GO:1901652 | BP | GO:1901652 | response to peptide | 10/276 | 412/29008 | 0.006425622 | 0.1491879 | 0.131853335 | Ager/Akt1/Nr1h4/Pld1/Ptpn22/Rab31/Rarres2/Serpina3m/Serpina3n/Slc39a14 | 10 |
| GO:0070997 | BP | GO:0070997 | neuron death | 10/276 | 428/29008 | 0.008288419 | 0.162786947 | 0.143872271 | Adarb1/Ager/Bcl2l1/Cd200r1/Ddit4/Hmox1/Lcn2/Lig4/Mt1/Prkcg | 10 |
| GO:0034248 | BP | GO:0034248 | regulation of cellular amide metabolic process | 10/276 | 448/29008 | 0.011171638 | 0.176063978 | 0.155606606 | Akt1/Cirbp/Eif4g3/Igf2bp2/Nr1h4/Pan2/Per1/Pld1/Pus7/Zcchc4 | 10 |
| GO:0006397 | BP | GO:0006397 | mRNA processing | 10/276 | 469/29008 | 0.014958628 | 0.190575279 | 0.168431797 | Adarb1/Cirbp/Pabpc1l/Pan2/Ptbp2/Rbfox3/Scaf1/Sfswap/Srek1/Zfp326 | 10 |
| GO:0045785 | BP | GO:0045785 | positive regulation of cell adhesion | 10/276 | 471/29008 | 0.015363767 | 0.191958105 | 0.169653949 | Ager/Chrd/Foxf1/Itga5/Map4k4/Ninj1/P2ry12/Plaur/Ptpn22/Spock2 | 10 |
| GO:0048608 | BP | GO:0048608 | reproductive structure development | 10/276 | 480/29008 | 0.017288776 | 0.202050271 | 0.178573478 | Adm/Akt1/Bcl2l1/Cdx4/Ggnbp2/Hmga2/Kmt2b/Plaur/Sema3a/Serpine1 | 10 |
| GO:0061458 | BP | GO:0061458 | reproductive system development | 10/276 | 484/29008 | 0.018199351 | 0.206528782 | 0.182531618 | Adm/Akt1/Bcl2l1/Cdx4/Ggnbp2/Hmga2/Kmt2b/Plaur/Sema3a/Serpine1 | 10 |
| GO:0006820 | BP | GO:0006820 | anion transport | 10/276 | 495/29008 | 0.020885512 | 0.220019621 | 0.194454918 | Abcc10/Clcnkb/Fgfr1/Lrrc8a/Nr1h4/Ros1/Slc10a6/Slc20a1/Slc25a18/Slc26a8 | 10 |
| GO:0004175 | MF | GO:0004175 | endopeptidase activity | 10/273 | 447/28438 | 0.011652336 | 0.282518209 | 0.258053759 | Acr/Adam11/Adamts15/Adamts5/Adamts6/Ctla2a/Ctsk/Hp/Pamr1/Tpsab1 | 10 |
| GO:0050920 | BP | GO:0050920 | regulation of chemotaxis | 9/276 | 229/29008 | 0.000370567 | 0.046121376 | 0.040762403 | Ager/Fgfr1/Ninj1/P2ry12/Rarres2/Sema3a/Sema6b/Serpine1/Vegfd | 9 |
| GO:0007568 | BP | GO:0007568 | aging | 9/276 | 262/29008 | 0.000969507 | 0.084792536 | 0.074940251 | Ager/Akt1/Apod/Cd68/Hmga2/Inpp5d/Serpine1/Smc5/Tbx2 | 9 |
| GO:0030336 | BP | GO:0030336 | negative regulation of cell migration | 9/276 | 293/29008 | 0.002090315 | 0.122986534 | 0.10869638 | Adarb1/Ager/Akt1/Apod/Cd200r1/Chrd/Mctp1/Sema3a/Serpine1 | 9 |
| GO:0090287 | BP | GO:0090287 | regulation of cellular response to growth factor stimulus | 9/276 | 299/29008 | 0.002395 | 0.132327381 | 0.116951888 | Chrd/Eid2/Fgfr1/Lox/Lrg1/Ulk1/Vegfd/Wfikkn1/Xdh | 9 |
| GO:0045765 | BP | GO:0045765 | regulation of angiogenesis | 9/276 | 302/29008 | 0.002560039 | 0.135615798 | 0.119858215 | Adm/Hmga2/Hmox1/Itga5/Lrg1/Ninj1/Rhoj/Serpine1/Vegfd | 9 |
| GO:1901342 | BP | GO:1901342 | regulation of vasculature development | 9/276 | 306/29008 | 0.002793987 | 0.136990007 | 0.121072751 | Adm/Hmga2/Hmox1/Itga5/Lrg1/Ninj1/Rhoj/Serpine1/Vegfd | 9 |
| GO:2000146 | BP | GO:2000146 | negative regulation of cell motility | 9/276 | 306/29008 | 0.002793987 | 0.136990007 | 0.121072751 | Adarb1/Ager/Akt1/Apod/Cd200r1/Chrd/Mctp1/Sema3a/Serpine1 | 9 |
| GO:0030198 | BP | GO:0030198 | extracellular matrix organization | 9/276 | 311/29008 | 0.003109868 | 0.14033381 | 0.124028028 | Adamts15/Adamts5/Adamts6/Foxf1/Gfod2/Lox/Ric8a/Spock2/Tpsab1 | 9 |
| GO:0043062 | BP | GO:0043062 | extracellular structure organization | 9/276 | 312/29008 | 0.003176297 | 0.14033381 | 0.124028028 | Adamts15/Adamts5/Adamts6/Foxf1/Gfod2/Lox/Ric8a/Spock2/Tpsab1 | 9 |
| GO:0045229 | BP | GO:0045229 | external encapsulating structure organization | 9/276 | 313/29008 | 0.003243839 | 0.14033381 | 0.124028028 | Adamts15/Adamts5/Adamts6/Foxf1/Gfod2/Lox/Ric8a/Spock2/Tpsab1 | 9 |
| GO:0051271 | BP | GO:0051271 | negative regulation of cellular component movement | 9/276 | 315/29008 | 0.003382316 | 0.14033381 | 0.124028028 | Adarb1/Ager/Akt1/Apod/Cd200r1/Chrd/Mctp1/Sema3a/Serpine1 | 9 |
| GO:0071216 | BP | GO:0071216 | cellular response to biotic stimulus | 9/276 | 337/29008 | 0.005233324 | 0.146677122 | 0.129634292 | Akt1/Cd68/Cxcl1/Nr1h4/Pf4/Ptpn22/Sbno2/Serpine1/Slx4 | 9 |
| GO:1901214 | BP | GO:1901214 | regulation of neuron death | 9/276 | 384/29008 | 0.011740158 | 0.180052843 | 0.159131993 | Ager/Bcl2l1/Cd200r1/Ddit4/Hmox1/Lcn2/Lig4/Mt1/Prkcg | 9 |
| GO:0006869 | BP | GO:0006869 | lipid transport | 9/276 | 387/29008 | 0.012299796 | 0.181049245 | 0.160012621 | Acsl6/Akt1/Apod/Msr1/Nr1h2/Nr1h4/Pitpnm1/Slc10a6/Soat2 | 9 |
| GO:0032496 | BP | GO:0032496 | response to lipopolysaccharide | 9/276 | 388/29008 | 0.012490694 | 0.181049245 | 0.160012621 | Adm/Akt1/Cd68/Cxcl1/Nr1h4/Pf4/Ptpn22/Sbno2/Serpine1 | 9 |
| GO:0006417 | BP | GO:0006417 | regulation of translation | 9/276 | 390/29008 | 0.012879106 | 0.182767167 | 0.161530932 | Akt1/Cirbp/Eif4g3/Igf2bp2/Pan2/Per1/Pld1/Pus7/Zcchc4 | 9 |
| GO:0002237 | BP | GO:0002237 | response to molecule of bacterial origin | 9/276 | 409/29008 | 0.017030387 | 0.200401203 | 0.17711602 | Adm/Akt1/Cd68/Cxcl1/Nr1h4/Pf4/Ptpn22/Sbno2/Serpine1 | 9 |
| GO:0002697 | BP | GO:0002697 | regulation of immune effector process | 9/276 | 424/29008 | 0.020941299 | 0.220019621 | 0.194454918 | Ager/Crtam/Cxcl1/Fcgr3/Foxf1/Gab2/Hmox1/Ptpn22/Pvr | 9 |
| GO:0007264 | BP | GO:0007264 | small GTPase mediated signal transduction | 9/276 | 425/29008 | 0.021223208 | 0.220545717 | 0.194919886 | Dennd4a/Hmox1/Kctd13/Map4k4/Myo9b/Rasa3/Rhoj/Ssx2ip/Ulk1 | 9 |
| GO:0010876 | BP | GO:0010876 | lipid localization | 9/276 | 437/29008 | 0.024822378 | 0.231905611 | 0.204959841 | Acsl6/Akt1/Apod/Msr1/Nr1h2/Nr1h4/Pitpnm1/Slc10a6/Soat2 | 9 |
| GO:0010959 | BP | GO:0010959 | regulation of metal ion transport | 9/276 | 443/29008 | 0.026776083 | 0.237235699 | 0.209670612 | Ager/Akt1/Cxcl1/Fgf11/Lcn2/P2ry12/Per1/Ptpn22/Serpine1 | 9 |
| GO:0002683 | BP | GO:0002683 | negative regulation of immune system process | 9/276 | 460/29008 | 0.032897408 | 0.251441119 | 0.222225463 | Akt1/Apod/Cd200r1/Cd68/Crtam/Foxf1/Hmox1/Inpp5d/Ptpn22 | 9 |
| GO:0050673 | BP | GO:0050673 | epithelial cell proliferation | 9/276 | 471/29008 | 0.037339429 | 0.269110006 | 0.237841353 | Ager/Akt1/Fgfr1/Hmga2/Hmox1/Lrg1/Sidt2/Vegfd/Xdh | 9 |
| GO:0042742 | BP | GO:0042742 | defense response to bacterium | 9/276 | 473/29008 | 0.038189049 | 0.273406558 | 0.241638676 | Adamts5/Adm/Hp/Lcn2/Nr1h4/Pld1/Rarres2/Serpine1/Wfdc17 | 9 |
| GO:1901681 | MF | GO:1901681 | sulfur compound binding | 9/273 | 280/28438 | 0.001630184 | 0.124458242 | 0.113680875 | Adamts15/Adamts5/Ager/Chrd/Fgfr1/Pf4/Saa1/Soat2/Zcchc4 | 9 |
| GO:0004674 | MF | GO:0004674 | protein serine/threonine kinase activity | 9/273 | 441/28438 | 0.027416217 | 0.306311797 | 0.27978696 | Akt1/Map3k6/Map4k4/Pim3/Prkcg/Prkd3/Riok1/Riok2/Ulk1 | 9 |
| GO:0071675 | BP | GO:0071675 | regulation of mononuclear cell migration | 8/276 | 118/29008 | 1.80E-05 | 0.011640979 | 0.010288381 | Ager/Akt1/Apod/Cd200r1/Msn/Ninj1/Rarres2/Serpine1 | 8 |
| GO:0010634 | BP | GO:0010634 | positive regulation of epithelial cell migration | 8/276 | 156/29008 | 0.000130708 | 0.036567108 | 0.032318272 | Akt1/Fgfr1/Gab2/Hmox1/Lcn2/Map4k4/Rhoj/Serpine1 | 8 |
| GO:0045766 | BP | GO:0045766 | positive regulation of angiogenesis | 8/276 | 179/29008 | 0.000333572 | 0.046121376 | 0.040762403 | Adm/Hmga2/Hmox1/Itga5/Lrg1/Ninj1/Serpine1/Vegfd | 8 |
| GO:1904018 | BP | GO:1904018 | positive regulation of vasculature development | 8/276 | 179/29008 | 0.000333572 | 0.046121376 | 0.040762403 | Adm/Hmga2/Hmox1/Itga5/Lrg1/Ninj1/Serpine1/Vegfd | 8 |
| GO:0045444 | BP | GO:0045444 | fat cell differentiation | 8/276 | 260/29008 | 0.003607772 | 0.14033381 | 0.124028028 | Akt1/Asxl1/Cby1/Clip3/Hmga2/Jdp2/Lrg1/Rarres2 | 8 |
| GO:0031349 | BP | GO:0031349 | positive regulation of defense response | 8/276 | 273/29008 | 0.004826136 | 0.146677122 | 0.129634292 | Ager/Crtam/Cxcl1/Fcgr3/Fgfr1/Ninj1/Pvr/Serpine1 | 8 |
| GO:0060485 | BP | GO:0060485 | mesenchyme development | 8/276 | 293/29008 | 0.00728782 | 0.160033945 | 0.141439148 | Ager/Fgfr1/Foxf1/Hmga2/Plaur/Sema3a/Sema6b/Tbx2 | 8 |
| GO:0002699 | BP | GO:0002699 | positive regulation of immune effector process | 8/276 | 296/29008 | 0.00772633 | 0.160271819 | 0.141649383 | Crtam/Cxcl1/Fcgr3/Foxf1/Gab2/Hmox1/Ptpn22/Pvr | 8 |
| GO:0002703 | BP | GO:0002703 | regulation of leukocyte mediated immunity | 8/276 | 297/29008 | 0.007876823 | 0.161325319 | 0.142580473 | Ager/Crtam/Cxcl1/Fcgr3/Foxf1/Gab2/Hmox1/Pvr | 8 |
| GO:0071222 | BP | GO:0071222 | cellular response to lipopolysaccharide | 8/276 | 303/29008 | 0.008826535 | 0.162786947 | 0.143872271 | Akt1/Cd68/Cxcl1/Nr1h4/Pf4/Ptpn22/Sbno2/Serpine1 | 8 |
| GO:0051402 | BP | GO:0051402 | neuron apoptotic process | 8/276 | 309/29008 | 0.009859415 | 0.16882604 | 0.149209665 | Adarb1/Ager/Bcl2l1/Hmox1/Lcn2/Lig4/Mt1/Prkcg | 8 |
| GO:0071219 | BP | GO:0071219 | cellular response to molecule of bacterial origin | 8/276 | 313/29008 | 0.010596273 | 0.176063978 | 0.155606606 | Akt1/Cd68/Cxcl1/Nr1h4/Pf4/Ptpn22/Sbno2/Serpine1 | 8 |
| GO:0007265 | BP | GO:0007265 | Ras protein signal transduction | 8/276 | 330/29008 | 0.014189593 | 0.183047176 | 0.161778407 | Dennd4a/Kctd13/Map4k4/Myo9b/Rasa3/Rhoj/Ssx2ip/Ulk1 | 8 |
| GO:0043434 | BP | GO:0043434 | response to peptide hormone | 8/276 | 351/29008 | 0.01976509 | 0.212964464 | 0.18821952 | Akt1/Nr1h4/Pld1/Rab31/Rarres2/Serpina3m/Serpina3n/Slc39a14 | 8 |
| GO:0031667 | BP | GO:0031667 | response to nutrient levels | 8/276 | 358/29008 | 0.021929279 | 0.221759838 | 0.195992934 | Akt1/Cd68/Kptn/Nprl2/Nr1h4/Prkcg/Trim24/Ulk1 | 8 |
| GO:0006644 | BP | GO:0006644 | phospholipid metabolic process | 8/276 | 360/29008 | 0.022577235 | 0.224939375 | 0.198803032 | Acsl6/Bpnt1/Chka/Inpp5d/Nr1h2/Nr1h4/Pld1/Smpd2 | 8 |
| GO:0042060 | BP | GO:0042060 | wound healing | 8/276 | 361/29008 | 0.02290623 | 0.226242151 | 0.199954435 | Ager/Fer1l5/Hmox1/Lox/P2ry12/Pf4/Serpine1/Tpsab1 | 8 |
| GO:0031589 | BP | GO:0031589 | cell-substrate adhesion | 8/276 | 366/29008 | 0.02460203 | 0.231905611 | 0.204959841 | Apod/Foxf1/Itga5/Itgbl1/Map4k4/Ninj1/Serpine1/Spock2 | 8 |
| GO:0051346 | BP | GO:0051346 | negative regulation of hydrolase activity | 8/276 | 374/29008 | 0.027495274 | 0.238538089 | 0.210821673 | Akt1/Plaur/Serpina3m/Serpina3n/Serpine1/Slc39a14/Tnnt2/Wfikkn1 | 8 |
| GO:0009991 | BP | GO:0009991 | response to extracellular stimulus | 8/276 | 391/29008 | 0.034410007 | 0.258548553 | 0.228507065 | Akt1/Cd68/Kptn/Nprl2/Nr1h4/Prkcg/Trim24/Ulk1 | 8 |
| GO:0042326 | BP | GO:0042326 | negative regulation of phosphorylation | 8/276 | 396/29008 | 0.036649607 | 0.268046568 | 0.236901478 | Adarb1/Ager/Akt1/Ddit4/Ggnbp2/Nprl2/Ptpn22/Xdh | 8 |
| GO:0051098 | BP | GO:0051098 | regulation of binding | 8/276 | 397/29008 | 0.037109043 | 0.268046568 | 0.236901478 | Ager/Akt1/Gas8/Hmga2/Hmox1/Lox/Plaur/Wfikkn1 | 8 |
| GO:0032102 | BP | GO:0032102 | negative regulation of response to external stimulus | 8/276 | 398/29008 | 0.037572344 | 0.270186898 | 0.238793117 | Cd200r1/Ctla2a/Foxf1/Nr1h4/Sema3a/Sema6b/Serpine1/Tpsab1 | 8 |
| GO:0030900 | BP | GO:0030900 | forebrain development | 8/276 | 400/29008 | 0.038510587 | 0.273628651 | 0.241834964 | Chrd/Fgfr1/Hmga2/Numbl/P2ry12/Rtn4rl2/Sema3a/Sema6b | 8 |
| GO:0050678 | BP | GO:0050678 | regulation of epithelial cell proliferation | 8/276 | 403/29008 | 0.039947221 | 0.276807721 | 0.244644648 | Ager/Akt1/Fgfr1/Hmga2/Hmox1/Lrg1/Vegfd/Xdh | 8 |
| GO:0048638 | BP | GO:0048638 | regulation of developmental growth | 8/276 | 406/29008 | 0.041419232 | 0.279338487 | 0.246881357 | Akt1/Fgfr1/Hmga2/Sema3a/Sema6b/Serpine1/Tbx2/Ulk1 | 8 |
| GO:0005774 | CC | GO:0005774 | vacuolar membrane | 8/268 | 228/28886 | 0.001371999 | 0.242964068 | 0.231304802 | Cd68/Ifitm2/Lrrc8a/Nprl2/Sbf2/Sidt2/Slc39a14/Ulk1 | 8 |
| GO:0062023 | CC | GO:0062023 | collagen-containing extracellular matrix | 8/268 | 393/28886 | 0.031101677 | 0.431208931 | 0.410516242 | Adamts15/Adamts5/Fcna/Lox/Pf4/Rarres2/Serpine1/Spock2 | 8 |
| GO:0043235 | CC | GO:0043235 | receptor complex | 8/268 | 408/28886 | 0.037487281 | 0.431208931 | 0.410516242 | Cd200r1/Fgfr1/Gabbr2/Itga5/Itgbl1/Nr1h4/Osmr/Ros1 | 8 |
| GO:0005539 | MF | GO:0005539 | glycosaminoglycan binding | 8/273 | 229/28438 | 0.001745907 | 0.124458242 | 0.113680875 | Adamts15/Adamts5/Ager/Chrd/Fgfr1/Pf4/Saa1/Spock2 | 8 |
| GO:0050839 | MF | GO:0050839 | cell adhesion molecule binding | 8/273 | 295/28438 | 0.007967859 | 0.279512649 | 0.255308464 | Cd200r1/Fgfr1/Itga5/Itgbl1/Msn/Ninj1/Pvr/Tspan4 | 8 |
| GO:0001227 | MF | GO:0001227 | DNA-binding transcription repressor activity, RNA polymerase II-specific | 8/273 | 328/28438 | 0.014400406 | 0.282518209 | 0.258053759 | E4f1/Jdp2/Peg3/Pou6f1/Prdm5/Tbx2/Zfp715/Zfp777 | 8 |
| GO:0001217 | MF | GO:0001217 | DNA-binding transcription repressor activity | 8/273 | 330/28438 | 0.01488452 | 0.282518209 | 0.258053759 | E4f1/Jdp2/Peg3/Pou6f1/Prdm5/Tbx2/Zfp715/Zfp777 | 8 |
| GO:0072676 | BP | GO:0072676 | lymphocyte migration | 7/276 | 107/29008 | 7.58E-05 | 0.03068009 | 0.027115283 | Akt1/Apod/Ccl6/Ccl9/Cd200r1/Crtam/Msn | 7 |
| GO:0019079 | BP | GO:0019079 | viral genome replication | 7/276 | 126/29008 | 0.000210752 | 0.043443702 | 0.038395856 | Adarb1/Hmga2/Ifi27/Ifitm2/Morc2a/N4bp1/Resf1 | 7 |
| GO:0001936 | BP | GO:0001936 | regulation of endothelial cell proliferation | 7/276 | 166/29008 | 0.001098012 | 0.088419149 | 0.078145478 | Ager/Akt1/Fgfr1/Hmox1/Lrg1/Vegfd/Xdh | 7 |
| GO:0032675 | BP | GO:0032675 | regulation of interleukin-6 production | 7/276 | 166/29008 | 0.001098012 | 0.088419149 | 0.078145478 | Ager/Cd200r1/Inpp5d/Nr1h4/Prg4/Ptpn22/Vegfd | 7 |
| GO:0032635 | BP | GO:0032635 | interleukin-6 production | 7/276 | 174/29008 | 0.001440505 | 0.103588325 | 0.091552105 | Ager/Cd200r1/Inpp5d/Nr1h4/Prg4/Ptpn22/Vegfd | 7 |
| GO:0001935 | BP | GO:0001935 | endothelial cell proliferation | 7/276 | 181/29008 | 0.001803942 | 0.116751144 | 0.103185498 | Ager/Akt1/Fgfr1/Hmox1/Lrg1/Vegfd/Xdh | 7 |
| GO:0030522 | BP | GO:0030522 | intracellular receptor signaling pathway | 7/276 | 217/29008 | 0.004917939 | 0.146677122 | 0.129634292 | Asxl1/Hmga2/Nr1h2/Nr1h4/Per1/Ptpn22/Trim24 | 7 |
| GO:0010810 | BP | GO:0010810 | regulation of cell-substrate adhesion | 7/276 | 226/29008 | 0.006109743 | 0.1491879 | 0.131853335 | Apod/Foxf1/Itga5/Map4k4/Ninj1/Serpine1/Spock2 | 7 |
| GO:0046777 | BP | GO:0046777 | protein autophosphorylation | 7/276 | 237/29008 | 0.007844611 | 0.161325319 | 0.142580473 | Fgfr1/Pim3/Prkcg/Riok2/Ros1/Trim24/Ulk1 | 7 |
| GO:0030100 | BP | GO:0030100 | regulation of endocytosis | 7/276 | 238/29008 | 0.008018621 | 0.162786947 | 0.143872271 | Ager/Bcl2l1/Clip3/Mctp1/Nr1h2/Rab31/Serpine1 | 7 |
| GO:0006814 | BP | GO:0006814 | sodium ion transport | 7/276 | 245/29008 | 0.009317449 | 0.166581574 | 0.147225989 | Akt1/Asic3/Cxcl1/Fgf11/Per1/Slc10a6/Slc20a1 | 7 |
| GO:0006260 | BP | GO:0006260 | DNA replication | 7/276 | 253/29008 | 0.010983319 | 0.176063978 | 0.155606606 | E4f1/Fgfr1/Kctd13/Lig4/Rev3l/Slx4/Tspyl2 | 7 |
| GO:1901215 | BP | GO:1901215 | negative regulation of neuron death | 7/276 | 255/29008 | 0.011431517 | 0.176154231 | 0.155686372 | Bcl2l1/Cd200r1/Hmox1/Lcn2/Lig4/Mt1/Prkcg | 7 |
| GO:0019058 | BP | GO:0019058 | viral life cycle | 7/276 | 257/29008 | 0.011892821 | 0.181049245 | 0.160012621 | Adarb1/Hmga2/Ifi27/Ifitm2/Morc2a/N4bp1/Resf1 | 7 |
| GO:0002274 | BP | GO:0002274 | myeloid leukocyte activation | 7/276 | 261/29008 | 0.012855564 | 0.182767167 | 0.161530932 | Ager/Fcgr3/Fer1l5/Foxf1/Gab2/Hmox1/Sbno2 | 7 |
| GO:0006898 | BP | GO:0006898 | receptor-mediated endocytosis | 7/276 | 265/29008 | 0.013873166 | 0.182767167 | 0.161530932 | Adm/Lmbr1l/Msr1/Rab31/Reps1/Serpine1/Ulk1 | 7 |
| GO:0043523 | BP | GO:0043523 | regulation of neuron apoptotic process | 7/276 | 276/29008 | 0.016967353 | 0.200401203 | 0.17711602 | Ager/Bcl2l1/Hmox1/Lcn2/Lig4/Mt1/Prkcg | 7 |
| GO:0015850 | BP | GO:0015850 | organic hydroxy compound transport | 7/276 | 282/29008 | 0.01884649 | 0.208147579 | 0.183962323 | Fcgr3/Msr1/Nr1h2/Nr1h4/P2ry12/Slc10a6/Soat2 | 7 |
| GO:0071496 | BP | GO:0071496 | cellular response to external stimulus | 7/276 | 295/29008 | 0.023409531 | 0.226242151 | 0.199954435 | Akt1/Cd68/Kptn/Nprl2/Nr1h4/Trim24/Ulk1 | 7 |
| GO:1901653 | BP | GO:1901653 | cellular response to peptide | 7/276 | 308/29008 | 0.028683066 | 0.240973085 | 0.21297374 | Ager/Akt1/Nr1h4/Ptpn22/Rab31/Rarres2/Slc39a14 | 7 |
| GO:0009895 | BP | GO:0009895 | negative regulation of catabolic process | 7/276 | 311/29008 | 0.03000542 | 0.243990254 | 0.215640336 | Akt1/Cirbp/Ddit4/Hmox1/N4bp1/Prkcg/Ptpn22 | 7 |
| GO:0009615 | BP | GO:0009615 | response to virus | 7/276 | 312/29008 | 0.030455189 | 0.243990254 | 0.215640336 | Bcl2l1/Ddit4/Ifi27/Ifitm2/Lcn2/Ptpn22/Tlr13 | 7 |
| GO:0015849 | BP | GO:0015849 | organic acid transport | 7/276 | 314/29008 | 0.031368303 | 0.244940523 | 0.21648019 | Acsl6/Akt1/Lrrc8a/Nr1h4/Slc10a6/Slc25a18/Slc26a8 | 7 |
| GO:0046651 | BP | GO:0046651 | lymphocyte proliferation | 7/276 | 330/29008 | 0.03933911 | 0.274011178 | 0.242173044 | Ager/Crtam/Impdh1/Inpp5d/Lmbr1l/Msn/Ptpn22 | 7 |
| GO:0032943 | BP | GO:0032943 | mononuclear cell proliferation | 7/276 | 334/29008 | 0.041520956 | 0.279338487 | 0.246881357 | Ager/Crtam/Impdh1/Inpp5d/Lmbr1l/Msn/Ptpn22 | 7 |
| GO:0044282 | BP | GO:0044282 | small molecule catabolic process | 7/276 | 335/29008 | 0.042078463 | 0.282501882 | 0.249677189 | Akt1/Bpnt1/Gda/Hao1/Hdc/Hgd/Xdh | 7 |
| GO:0008202 | BP | GO:0008202 | steroid metabolic process | 7/276 | 337/29008 | 0.043208015 | 0.287323622 | 0.253938677 | Adm/Dkk3/Fgfr1/Nr1h4/Saa1/Soat2/Ugt1a7c | 7 |
| GO:0006066 | BP | GO:0006066 | alcohol metabolic process | 7/276 | 344/29008 | 0.04731518 | 0.301995904 | 0.266906145 | Bpnt1/Chka/Dkk3/Fgfr1/Hao1/Saa1/Soat2 | 7 |
| GO:0016032 | BP | GO:0016032 | viral process | 7/276 | 346/29008 | 0.048532904 | 0.303776555 | 0.268479897 | Adarb1/Hmga2/Ifi27/Ifitm2/Morc2a/N4bp1/Resf1 | 7 |
| GO:0098791 | CC | GO:0098791 | Golgi apparatus subcompartment | 7/268 | 307/28886 | 0.025092235 | 0.431208931 | 0.410516242 | Ap4b1/Cby1/Chid1/Clip3/Golga5/Pld1/Rab31 | 7 |
| GO:0008201 | MF | GO:0008201 | heparin binding | 7/273 | 169/28438 | 0.001280768 | 0.124458242 | 0.113680875 | Adamts15/Adamts5/Ager/Chrd/Fgfr1/Pf4/Saa1 | 7 |
| GO:0008509 | MF | GO:0008509 | anion transmembrane transporter activity | 7/273 | 312/28438 | 0.031720727 | 0.306311797 | 0.27978696 | Asic3/Clcnkb/Lrrc8a/Slc10a6/Slc25a18/Slc26a8/Slc39a14 | 7 |
| GO:0005126 | MF | GO:0005126 | cytokine receptor binding | 7/273 | 315/28438 | 0.03314797 | 0.306311797 | 0.27978696 | Ccl6/Ccl9/Cxcl1/Lrg1/Osmr/Pf4/Vegfd | 7 |
| GO:0001664 | MF | GO:0001664 | G protein-coupled receptor binding | 7/273 | 318/28438 | 0.034617479 | 0.314074941 | 0.28687786 | Adm/Ccl6/Ccl9/Cxcl1/Fcna/Gnal/Pf4 | 7 |
| GO:0061629 | MF | GO:0061629 | RNA polymerase II-specific DNA-binding transcription factor binding | 7/273 | 329/28438 | 0.040373787 | 0.341466438 | 0.311897411 | Asxl1/Ifi27/Lrif1/Nr1h2/Nr1h4/Tbp/Trim24 | 7 |
| GO:0045069 | BP | GO:0045069 | regulation of viral genome replication | 6/276 | 90/29008 | 0.000224142 | 0.043443702 | 0.038395856 | Adarb1/Hmga2/Ifitm2/Morc2a/N4bp1/Resf1 | 6 |
| GO:0002526 | BP | GO:0002526 | acute inflammatory response | 6/276 | 110/29008 | 0.0006584 | 0.0704957 | 0.062304605 | Cd163/Cxcl1/Fcgr3/Hp/Saa1/Serpina3n | 6 |
| GO:0071621 | BP | GO:0071621 | granulocyte chemotaxis | 6/276 | 127/29008 | 0.001389984 | 0.102227029 | 0.090348981 | Ccl6/Ccl9/Cxcl1/Fcgr3/Pf4/Rarres2 | 6 |
| GO:0015698 | BP | GO:0015698 | inorganic anion transport | 6/276 | 151/29008 | 0.00331635 | 0.14033381 | 0.124028028 | Clcnkb/Fgfr1/Lrrc8a/Ros1/Slc20a1/Slc26a8 | 6 |
| GO:0050921 | BP | GO:0050921 | positive regulation of chemotaxis | 6/276 | 151/29008 | 0.00331635 | 0.14033381 | 0.124028028 | Ager/Fgfr1/P2ry12/Rarres2/Serpine1/Vegfd | 6 |
| GO:0031099 | BP | GO:0031099 | regeneration | 6/276 | 153/29008 | 0.003537755 | 0.14033381 | 0.124028028 | Adm/Hmox1/Ninj1/Rtn4rl2/Serpine1/Ulk1 | 6 |
| GO:1903900 | BP | GO:1903900 | regulation of viral life cycle | 6/276 | 153/29008 | 0.003537755 | 0.14033381 | 0.124028028 | Adarb1/Hmga2/Ifitm2/Morc2a/N4bp1/Resf1 | 6 |
| GO:0097530 | BP | GO:0097530 | granulocyte migration | 6/276 | 157/29008 | 0.004013294 | 0.142521759 | 0.125961753 | Ccl6/Ccl9/Cxcl1/Fcgr3/Pf4/Rarres2 | 6 |
| GO:0010594 | BP | GO:0010594 | regulation of endothelial cell migration | 6/276 | 174/29008 | 0.006575278 | 0.1491879 | 0.131853335 | Ager/Akt1/Fgfr1/Hmox1/Lcn2/Rhoj | 6 |
| GO:0050792 | BP | GO:0050792 | regulation of viral process | 6/276 | 174/29008 | 0.006575278 | 0.1491879 | 0.131853335 | Adarb1/Hmga2/Ifitm2/Morc2a/N4bp1/Resf1 | 6 |
| GO:0007369 | BP | GO:0007369 | gastrulation | 6/276 | 178/29008 | 0.007319229 | 0.160033945 | 0.141439148 | Chrd/Fgfr1/Foxf1/Hmga2/Itga5/Ric8a | 6 |
| GO:0043524 | BP | GO:0043524 | negative regulation of neuron apoptotic process | 6/276 | 184/29008 | 0.008546564 | 0.162786947 | 0.143872271 | Bcl2l1/Hmox1/Lcn2/Lig4/Mt1/Prkcg | 6 |
| GO:0033044 | BP | GO:0033044 | regulation of chromosome organization | 6/276 | 185/29008 | 0.008764614 | 0.162786947 | 0.143872271 | Lig4/Morc2a/Resf1/Riok2/Slx4/Smc5 | 6 |
| GO:0032872 | BP | GO:0032872 | regulation of stress-activated MAPK cascade | 6/276 | 198/29008 | 0.011973077 | 0.181049245 | 0.160012621 | Ager/Akt1/Map4k4/Per1/Ptpn22/Xdh | 6 |
| GO:0031669 | BP | GO:0031669 | cellular response to nutrient levels | 6/276 | 199/29008 | 0.012249931 | 0.181049245 | 0.160012621 | Cd68/Kptn/Nprl2/Nr1h4/Trim24/Ulk1 | 6 |
| GO:0046578 | BP | GO:0046578 | regulation of Ras protein signal transduction | 6/276 | 200/29008 | 0.012531259 | 0.181049245 | 0.160012621 | Dennd4a/Kctd13/Map4k4/Myo9b/Rasa3/Ssx2ip | 6 |
| GO:0070302 | BP | GO:0070302 | regulation of stress-activated protein kinase signaling cascade | 6/276 | 201/29008 | 0.012817097 | 0.182767167 | 0.161530932 | Ager/Akt1/Map4k4/Per1/Ptpn22/Xdh | 6 |
| GO:0045017 | BP | GO:0045017 | glycerolipid biosynthetic process | 6/276 | 208/29008 | 0.014947137 | 0.190575279 | 0.168431797 | Acsl6/Bpnt1/Chka/Nr1h2/Nr1h4/Pld1 | 6 |
| GO:0006839 | BP | GO:0006839 | mitochondrial transport | 6/276 | 212/29008 | 0.01626879 | 0.199792568 | 0.176578104 | Akt1/Bcl2l1/Slc25a18/Slc25a28/Timm22/Tomm34 | 6 |
| GO:0050679 | BP | GO:0050679 | positive regulation of epithelial cell proliferation | 6/276 | 215/29008 | 0.017311513 | 0.202050271 | 0.178573478 | Akt1/Fgfr1/Hmga2/Hmox1/Lrg1/Vegfd | 6 |
| GO:0043542 | BP | GO:0043542 | endothelial cell migration | 6/276 | 221/29008 | 0.019533034 | 0.212964464 | 0.18821952 | Ager/Akt1/Fgfr1/Hmox1/Lcn2/Rhoj | 6 |
| GO:0031668 | BP | GO:0031668 | cellular response to extracellular stimulus | 6/276 | 231/29008 | 0.023653825 | 0.226242151 | 0.199954435 | Cd68/Kptn/Nprl2/Nr1h4/Trim24/Ulk1 | 6 |
| GO:0051056 | BP | GO:0051056 | regulation of small GTPase mediated signal transduction | 6/276 | 233/29008 | 0.024542738 | 0.231905611 | 0.204959841 | Dennd4a/Kctd13/Map4k4/Myo9b/Rasa3/Ssx2ip | 6 |
| GO:0048762 | BP | GO:0048762 | mesenchymal cell differentiation | 6/276 | 237/29008 | 0.026386987 | 0.237235699 | 0.209670612 | Ager/Fgfr1/Hmga2/Plaur/Sema3a/Sema6b | 6 |
| GO:0051403 | BP | GO:0051403 | stress-activated MAPK cascade | 6/276 | 238/29008 | 0.026862031 | 0.237235699 | 0.209670612 | Ager/Akt1/Map4k4/Per1/Ptpn22/Xdh | 6 |
| GO:0097191 | BP | GO:0097191 | extrinsic apoptotic signaling pathway | 6/276 | 238/29008 | 0.026862031 | 0.237235699 | 0.209670612 | Bcl2l1/Fgfr1/Hmox1/Ifi27/Lcn2/Serpine1 | 6 |
| GO:0072593 | BP | GO:0072593 | reactive oxygen species metabolic process | 6/276 | 244/29008 | 0.029831536 | 0.243990254 | 0.215640336 | Ager/Akt1/Cxcl1/Ddit4/Lcn2/Xdh | 6 |
| GO:0031330 | BP | GO:0031330 | negative regulation of cellular catabolic process | 6/276 | 245/29008 | 0.030346534 | 0.243990254 | 0.215640336 | Akt1/Cirbp/Hmox1/N4bp1/Prkcg/Ptpn22 | 6 |
| GO:0031098 | BP | GO:0031098 | stress-activated protein kinase signaling cascade | 6/276 | 248/29008 | 0.031926374 | 0.248350353 | 0.219493822 | Ager/Akt1/Map4k4/Per1/Ptpn22/Xdh | 6 |
| GO:0033002 | BP | GO:0033002 | muscle cell proliferation | 6/276 | 255/29008 | 0.035818426 | 0.262830901 | 0.232291835 | Ager/Akt1/Apod/Fgfr1/Hmox1/Tbx2 | 6 |
| GO:0021537 | BP | GO:0021537 | telencephalon development | 6/276 | 257/29008 | 0.036984002 | 0.268046568 | 0.236901478 | Fgfr1/Numbl/P2ry12/Rtn4rl2/Sema3a/Sema6b | 6 |
| GO:0010506 | BP | GO:0010506 | regulation of autophagy | 6/276 | 259/29008 | 0.038173628 | 0.273406558 | 0.241638676 | Ager/Akt1/Hmox1/Nprl2/Ptpn22/Ulk1 | 6 |
| GO:0010466 | BP | GO:0010466 | negative regulation of peptidase activity | 6/276 | 261/29008 | 0.039387426 | 0.274011178 | 0.242173044 | Akt1/Plaur/Serpina3m/Serpina3n/Serpine1/Wfikkn1 | 6 |
| GO:0006650 | BP | GO:0006650 | glycerophospholipid metabolic process | 6/276 | 273/29008 | 0.047183855 | 0.301752874 | 0.266691353 | Bpnt1/Chka/Inpp5d/Nr1h2/Nr1h4/Pld1 | 6 |
| GO:0005765 | CC | GO:0005765 | lysosomal membrane | 6/268 | 171/28886 | 0.00537021 | 0.242964068 | 0.231304802 | Cd68/Ifitm2/Lrrc8a/Nprl2/Sidt2/Slc39a14 | 6 |
| GO:0098852 | CC | GO:0098852 | lytic vacuole membrane | 6/268 | 171/28886 | 0.00537021 | 0.242964068 | 0.231304802 | Cd68/Ifitm2/Lrrc8a/Nprl2/Sidt2/Slc39a14 | 6 |
| GO:0004866 | MF | GO:0004866 | endopeptidase inhibitor activity | 6/273 | 215/28438 | 0.017994934 | 0.282518209 | 0.258053759 | Bcl2l1/Serpina3m/Serpina3n/Serpine1/Wfdc17/Wfikkn1 | 6 |
| GO:0030414 | MF | GO:0030414 | peptidase inhibitor activity | 6/273 | 223/28438 | 0.021105763 | 0.289586114 | 0.264509625 | Bcl2l1/Serpina3m/Serpina3n/Serpine1/Wfdc17/Wfikkn1 | 6 |
| GO:0019902 | MF | GO:0019902 | phosphatase binding | 6/273 | 230/28438 | 0.024110701 | 0.306311797 | 0.27978696 | Akt1/Ell/Gab2/Ppp1r18/Ros1/Sbf2 | 6 |
| GO:0061135 | MF | GO:0061135 | endopeptidase regulator activity | 6/273 | 231/28438 | 0.024562138 | 0.306311797 | 0.27978696 | Bcl2l1/Serpina3m/Serpina3n/Serpine1/Wfdc17/Wfikkn1 | 6 |
| GO:0106310 | MF | GO:0106310 | protein serine kinase activity | 6/273 | 246/28438 | 0.032021739 | 0.306311797 | 0.27978696 | Akt1/Map4k4/Pim3/Riok1/Riok2/Ulk1 | 6 |
| GO:0061134 | MF | GO:0061134 | peptidase regulator activity | 6/273 | 265/28438 | 0.043402009 | 0.360960039 | 0.329702979 | Bcl2l1/Serpina3m/Serpina3n/Serpine1/Wfdc17/Wfikkn1 | 6 |
| GO:0035091 | MF | GO:0035091 | phosphatidylinositol binding | 6/273 | 268/28438 | 0.045403188 | 0.37141296 | 0.339250737 | Akt1/Exoc1/Gab2/Pitpnm1/Pld1/Sbf2 | 6 |
| GO:0032715 | BP | GO:0032715 | negative regulation of interleukin-6 production | 5/276 | 52/29008 | 0.000135601 | 0.036567108 | 0.032318272 | Cd200r1/Inpp5d/Nr1h4/Prg4/Ptpn22 | 5 |
| GO:0045071 | BP | GO:0045071 | negative regulation of viral genome replication | 5/276 | 58/29008 | 0.000228227 | 0.043443702 | 0.038395856 | Hmga2/Ifitm2/Morc2a/N4bp1/Resf1 | 5 |
| GO:0002548 | BP | GO:0002548 | monocyte chemotaxis | 5/276 | 59/29008 | 0.000247439 | 0.044483979 | 0.03931526 | Ccl6/Ccl9/Myo9b/Ninj1/Serpine1 | 5 |
| GO:1905517 | BP | GO:1905517 | macrophage migration | 5/276 | 62/29008 | 0.000312497 | 0.046121376 | 0.040762403 | Cd200r1/Myo9b/Ninj1/P2ry12/Rarres2 | 5 |
| GO:1990868 | BP | GO:1990868 | response to chemokine | 5/276 | 71/29008 | 0.000586563 | 0.067789909 | 0.059913207 | Ccl6/Ccl9/Cxcl1/Lox/Pf4 | 5 |
| GO:1990869 | BP | GO:1990869 | cellular response to chemokine | 5/276 | 71/29008 | 0.000586563 | 0.067789909 | 0.059913207 | Ccl6/Ccl9/Cxcl1/Lox/Pf4 | 5 |
| GO:0002886 | BP | GO:0002886 | regulation of myeloid leukocyte mediated immunity | 5/276 | 75/29008 | 0.000753952 | 0.073933008 | 0.065342522 | Cxcl1/Fcgr3/Foxf1/Gab2/Hmox1 | 5 |
| GO:2001252 | BP | GO:2001252 | positive regulation of chromosome organization | 5/276 | 82/29008 | 0.001129399 | 0.088419149 | 0.078145478 | Lig4/Morc2a/Resf1/Slx4/Smc5 | 5 |
| GO:0001938 | BP | GO:0001938 | positive regulation of endothelial cell proliferation | 5/276 | 99/29008 | 0.002598325 | 0.135615798 | 0.119858215 | Akt1/Fgfr1/Hmox1/Lrg1/Vegfd | 5 |
| GO:0001704 | BP | GO:0001704 | formation of primary germ layer | 5/276 | 102/29008 | 0.002957135 | 0.138685366 | 0.122571121 | Chrd/Fgfr1/Foxf1/Hmga2/Itga5 | 5 |
| GO:0030593 | BP | GO:0030593 | neutrophil chemotaxis | 5/276 | 102/29008 | 0.002957135 | 0.138685366 | 0.122571121 | Ccl6/Ccl9/Cxcl1/Fcgr3/Pf4 | 5 |
| GO:0048525 | BP | GO:0048525 | negative regulation of viral process | 5/276 | 102/29008 | 0.002957135 | 0.138685366 | 0.122571121 | Hmga2/Ifitm2/Morc2a/N4bp1/Resf1 | 5 |
| GO:0010595 | BP | GO:0010595 | positive regulation of endothelial cell migration | 5/276 | 109/29008 | 0.003931565 | 0.141361587 | 0.124936384 | Akt1/Fgfr1/Hmox1/Lcn2/Rhoj | 5 |
| GO:0050764 | BP | GO:0050764 | regulation of phagocytosis | 5/276 | 115/29008 | 0.004933549 | 0.146677122 | 0.129634292 | Ager/Fcgr3/Fer1l5/Prkcg/Rab31 | 5 |
| GO:0002275 | BP | GO:0002275 | myeloid cell activation involved in immune response | 5/276 | 118/29008 | 0.005497021 | 0.146677122 | 0.129634292 | Fer1l5/Foxf1/Gab2/Hmox1/Sbno2 | 5 |
| GO:0007498 | BP | GO:0007498 | mesoderm development | 5/276 | 119/29008 | 0.005694535 | 0.147420133 | 0.13029097 | Chrd/Fgfr1/Foxf1/Hmga2/Pus7 | 5 |
| GO:0002040 | BP | GO:0002040 | sprouting angiogenesis | 5/276 | 123/29008 | 0.006534761 | 0.1491879 | 0.131853335 | Akt1/Hmox1/Itga5/Rhoj/Vegfd | 5 |
| GO:0040029 | BP | GO:0040029 | regulation of gene expression, epigenetic | 5/276 | 123/29008 | 0.006534761 | 0.1491879 | 0.131853335 | Hmga2/Lrif1/Morc2a/Pabpc1l/Resf1 | 5 |
| GO:0051101 | BP | GO:0051101 | regulation of DNA binding | 5/276 | 127/29008 | 0.007458287 | 0.160271819 | 0.141649383 | Ager/Hmga2/Hmox1/Plaur/Wfikkn1 | 5 |
| GO:0045807 | BP | GO:0045807 | positive regulation of endocytosis | 5/276 | 128/29008 | 0.007702651 | 0.160271819 | 0.141649383 | Ager/Bcl2l1/Clip3/Rab31/Serpine1 | 5 |
| GO:1990266 | BP | GO:1990266 | neutrophil migration | 5/276 | 128/29008 | 0.007702651 | 0.160271819 | 0.141649383 | Ccl6/Ccl9/Cxcl1/Fcgr3/Pf4 | 5 |
| GO:0002444 | BP | GO:0002444 | myeloid leukocyte mediated immunity | 5/276 | 130/29008 | 0.008207987 | 0.162786947 | 0.143872271 | Cxcl1/Fcgr3/Foxf1/Gab2/Hmox1 | 5 |
| GO:0050729 | BP | GO:0050729 | positive regulation of inflammatory response | 5/276 | 134/29008 | 0.009286769 | 0.166581574 | 0.147225989 | Ager/Fcgr3/Fgfr1/Ninj1/Serpine1 | 5 |
| GO:0055076 | BP | GO:0055076 | transition metal ion homeostasis | 5/276 | 138/29008 | 0.010459492 | 0.176063978 | 0.155606606 | Hmox1/Lcn2/Mt1/Slc25a28/Slc39a14 | 5 |
| GO:0010811 | BP | GO:0010811 | positive regulation of cell-substrate adhesion | 5/276 | 139/29008 | 0.01076778 | 0.176063978 | 0.155606606 | Foxf1/Itga5/Map4k4/Ninj1/Spock2 | 5 |
| GO:0046328 | BP | GO:0046328 | regulation of JNK cascade | 5/276 | 143/29008 | 0.012063025 | 0.181049245 | 0.160012621 | Ager/Akt1/Map4k4/Per1/Ptpn22 | 5 |
| GO:0071333 | BP | GO:0071333 | cellular response to glucose stimulus | 5/276 | 146/29008 | 0.013101162 | 0.182767167 | 0.161530932 | Ager/Nr1h4/Pim3/Sidt2/Slc39a14 | 5 |
| GO:0071331 | BP | GO:0071331 | cellular response to hexose stimulus | 5/276 | 148/29008 | 0.0138258 | 0.182767167 | 0.161530932 | Ager/Nr1h4/Pim3/Sidt2/Slc39a14 | 5 |
| GO:0071326 | BP | GO:0071326 | cellular response to monosaccharide stimulus | 5/276 | 149/29008 | 0.014198035 | 0.183047176 | 0.161778407 | Ager/Nr1h4/Pim3/Sidt2/Slc39a14 | 5 |
| GO:0002687 | BP | GO:0002687 | positive regulation of leukocyte migration | 5/276 | 153/29008 | 0.015754225 | 0.195328241 | 0.172632499 | Ager/P2ry12/Rarres2/Serpine1/Vegfd | 5 |
| GO:0046434 | BP | GO:0046434 | organophosphate catabolic process | 5/276 | 154/29008 | 0.016160323 | 0.199598494 | 0.17640658 | Bpnt1/Gda/Pld1/Smpd2/Xdh | 5 |
| GO:0043484 | BP | GO:0043484 | regulation of RNA splicing | 5/276 | 157/29008 | 0.01742028 | 0.202050271 | 0.178573478 | Cirbp/Ptbp2/Rbfox3/Sfswap/Zfp326 | 5 |
| GO:0071322 | BP | GO:0071322 | cellular response to carbohydrate stimulus | 5/276 | 157/29008 | 0.01742028 | 0.202050271 | 0.178573478 | Ager/Nr1h4/Pim3/Sidt2/Slc39a14 | 5 |
| GO:2000377 | BP | GO:2000377 | regulation of reactive oxygen species metabolic process | 5/276 | 160/29008 | 0.018743658 | 0.207720809 | 0.18358514 | Ager/Akt1/Cxcl1/Lcn2/Xdh | 5 |
| GO:0001678 | BP | GO:0001678 | cellular glucose homeostasis | 5/276 | 166/29008 | 0.021585203 | 0.220545717 | 0.194919886 | Ager/Nr1h4/Pim3/Sidt2/Slc39a14 | 5 |
| GO:0009266 | BP | GO:0009266 | response to temperature stimulus | 5/276 | 167/29008 | 0.022084505 | 0.222633828 | 0.196765373 | Akt1/Asic3/Cirbp/Hmox1/Rasa3 | 5 |
| GO:0001909 | BP | GO:0001909 | leukocyte mediated cytotoxicity | 5/276 | 168/29008 | 0.022591254 | 0.224939375 | 0.198803032 | Ager/Crtam/Cxcl1/Fcgr3/Pvr | 5 |
| GO:0007254 | BP | GO:0007254 | JNK cascade | 5/276 | 170/29008 | 0.023627231 | 0.226242151 | 0.199954435 | Ager/Akt1/Map4k4/Per1/Ptpn22 | 5 |
| GO:0019730 | BP | GO:0019730 | antimicrobial humoral response | 5/276 | 170/29008 | 0.023627231 | 0.226242151 | 0.199954435 | Adm/Cxcl1/Pf4/Rarres2/Wfdc17 | 5 |
| GO:0019827 | BP | GO:0019827 | stem cell population maintenance | 5/276 | 172/29008 | 0.024693412 | 0.231905611 | 0.204959841 | Fgfr1/Hmga2/Lig4/Med15/Smc5 | 5 |
| GO:0001890 | BP | GO:0001890 | placenta development | 5/276 | 174/29008 | 0.025790061 | 0.236421067 | 0.208950634 | Adm/Akt1/Cdx4/Ggnbp2/Serpine1 | 5 |
| GO:0098727 | BP | GO:0098727 | maintenance of cell number | 5/276 | 176/29008 | 0.02691743 | 0.237235699 | 0.209670612 | Fgfr1/Hmga2/Lig4/Med15/Smc5 | 5 |
| GO:0071466 | BP | GO:0071466 | cellular response to xenobiotic stimulus | 5/276 | 177/29008 | 0.027492709 | 0.238538089 | 0.210821673 | Fmo2/Hmga2/Nr1h4/Serpine1/Ugt1a7c | 5 |
| GO:0098739 | BP | GO:0098739 | import across plasma membrane | 5/276 | 180/29008 | 0.029265273 | 0.240973085 | 0.21297374 | Acsl6/Akt1/Lcn2/Lrrc8a/Slc39a14 | 5 |
| GO:0009749 | BP | GO:0009749 | response to glucose | 5/276 | 186/29008 | 0.033023015 | 0.251441119 | 0.222225463 | Ager/Nr1h4/Pim3/Sidt2/Slc39a14 | 5 |
| GO:1903305 | BP | GO:1903305 | regulation of regulated secretory pathway | 5/276 | 186/29008 | 0.033023015 | 0.251441119 | 0.222225463 | Bcl2l1/Foxf1/Gab2/Hmox1/Prkcg | 5 |
| GO:0010951 | BP | GO:0010951 | negative regulation of endopeptidase activity | 5/276 | 187/29008 | 0.033677146 | 0.255220715 | 0.225565898 | Akt1/Plaur/Serpina3m/Serpina3n/Serpine1 | 5 |
| GO:0009746 | BP | GO:0009746 | response to hexose | 5/276 | 189/29008 | 0.035009468 | 0.258548553 | 0.228507065 | Ager/Nr1h4/Pim3/Sidt2/Slc39a14 | 5 |
| GO:0034284 | BP | GO:0034284 | response to monosaccharide | 5/276 | 190/29008 | 0.035687697 | 0.262466791 | 0.231970032 | Ager/Nr1h4/Pim3/Sidt2/Slc39a14 | 5 |
| GO:0015931 | BP | GO:0015931 | nucleobase-containing compound transport | 5/276 | 195/29008 | 0.039200241 | 0.274011178 | 0.242173044 | Cetn2/Igf2bp2/Lrrc8a/Nupl2/Sidt2 | 5 |
| GO:0061351 | BP | GO:0061351 | neural precursor cell proliferation | 5/276 | 197/29008 | 0.040662206 | 0.279338487 | 0.246881357 | Acsl6/Fgfr1/Hmga2/Numbl/Ptbp2 | 5 |
| GO:0002695 | BP | GO:0002695 | negative regulation of leukocyte activation | 5/276 | 200/29008 | 0.042916509 | 0.287323622 | 0.253938677 | Crtam/Foxf1/Hmox1/Inpp5d/Ptpn22 | 5 |
| GO:0030308 | BP | GO:0030308 | negative regulation of cell growth | 5/276 | 202/29008 | 0.044460414 | 0.291242714 | 0.257402399 | Cirbp/Sema3a/Sema6b/Tspyl2/Ulk1 | 5 |
| GO:0009743 | BP | GO:0009743 | response to carbohydrate | 5/276 | 204/29008 | 0.046037237 | 0.296765932 | 0.262283858 | Ager/Nr1h4/Pim3/Sidt2/Slc39a14 | 5 |
| GO:0043491 | BP | GO:0043491 | protein kinase B signaling | 5/276 | 205/29008 | 0.046838012 | 0.300134268 | 0.265260818 | Akt1/Fgfr1/Lox/P2ry12/Xdh | 5 |
| GO:0021953 | BP | GO:0021953 | central nervous system neuron differentiation | 5/276 | 207/29008 | 0.048464322 | 0.303776555 | 0.268479897 | Adarb1/Fgfr1/Scyl3/Sema3a/Ulk1 | 5 |
| GO:0032869 | BP | GO:0032869 | cellular response to insulin stimulus | 5/276 | 208/29008 | 0.049289869 | 0.307325655 | 0.271616617 | Akt1/Nr1h4/Rab31/Rarres2/Slc39a14 | 5 |
| GO:0009898 | CC | GO:0009898 | cytoplasmic side of plasma membrane | 5/268 | 177/28886 | 0.025020863 | 0.431208931 | 0.410516242 | Exoc1/Gnal/Msn/Ptpn22/Rasa3 | 5 |
| GO:0036064 | CC | GO:0036064 | ciliary basal body | 5/268 | 179/28886 | 0.026098681 | 0.431208931 | 0.410516242 | Akt1/Cby1/Cetn2/Gas8/Ssx2ip | 5 |
| GO:0098562 | CC | GO:0098562 | cytoplasmic side of membrane | 5/268 | 194/28886 | 0.035129254 | 0.431208931 | 0.410516242 | Exoc1/Gnal/Msn/Ptpn22/Rasa3 | 5 |
| GO:0004867 | MF | GO:0004867 | serine-type endopeptidase inhibitor activity | 5/273 | 129/28438 | 0.008243047 | 0.279512649 | 0.255308464 | Serpina3m/Serpina3n/Serpine1/Wfdc17/Wfikkn1 | 5 |
| GO:0016922 | MF | GO:0016922 | nuclear receptor binding | 5/273 | 155/28438 | 0.017149983 | 0.282518209 | 0.258053759 | Asxl1/Lrif1/Nr1h2/Nr1h4/Trim24 | 5 |
| GO:0008514 | MF | GO:0008514 | organic anion transmembrane transporter activity | 5/273 | 184/28438 | 0.032782623 | 0.306311797 | 0.27978696 | Asic3/Lrrc8a/Slc10a6/Slc25a18/Slc26a8 | 5 |
| GO:0006953 | BP | GO:0006953 | acute-phase response | 4/276 | 41/29008 | 0.00061558 | 0.068690262 | 0.060708946 | Cd163/Hp/Saa1/Serpina3n | 4 |
| GO:1900744 | BP | GO:1900744 | regulation of p38MAPK cascade | 4/276 | 42/29008 | 0.00067533 | 0.0704957 | 0.062304605 | Ager/Per1/Ptpn22/Xdh | 4 |
| GO:0038066 | BP | GO:0038066 | p38MAPK cascade | 4/276 | 53/29008 | 0.001628038 | 0.114242269 | 0.100968137 | Ager/Per1/Ptpn22/Xdh | 4 |
| GO:0034113 | BP | GO:0034113 | heterotypic cell-cell adhesion | 4/276 | 56/29008 | 0.001997108 | 0.119678551 | 0.105772762 | Ager/Cd200r1/Itga5/Ninj1 | 4 |
| GO:0043536 | BP | GO:0043536 | positive regulation of blood vessel endothelial cell migration | 4/276 | 56/29008 | 0.001997108 | 0.119678551 | 0.105772762 | Akt1/Fgfr1/Hmox1/Rhoj | 4 |
| GO:0060711 | BP | GO:0060711 | labyrinthine layer development | 4/276 | 60/29008 | 0.002574048 | 0.135615798 | 0.119858215 | Adm/Akt1/Cdx4/Ggnbp2 | 4 |
| GO:0070098 | BP | GO:0070098 | chemokine-mediated signaling pathway | 4/276 | 61/29008 | 0.002734366 | 0.136990007 | 0.121072751 | Ccl6/Ccl9/Cxcl1/Pf4 | 4 |
| GO:2000401 | BP | GO:2000401 | regulation of lymphocyte migration | 4/276 | 63/29008 | 0.00307526 | 0.14033381 | 0.124028028 | Akt1/Apod/Cd200r1/Msn | 4 |
| GO:0001707 | BP | GO:0001707 | mesoderm formation | 4/276 | 66/29008 | 0.003639266 | 0.14033381 | 0.124028028 | Chrd/Fgfr1/Foxf1/Hmga2 | 4 |
| GO:0000768 | BP | GO:0000768 | syncytium formation by plasma membrane fusion | 4/276 | 67/29008 | 0.00384184 | 0.14033381 | 0.124028028 | Adamts15/Adamts5/Fer1l5/Sbno2 | 4 |
| GO:0140253 | BP | GO:0140253 | cell-cell fusion | 4/276 | 67/29008 | 0.00384184 | 0.14033381 | 0.124028028 | Adamts15/Adamts5/Fer1l5/Sbno2 | 4 |
| GO:0097006 | BP | GO:0097006 | regulation of plasma lipoprotein particle levels | 4/276 | 68/29008 | 0.004051917 | 0.142521759 | 0.125961753 | Msr1/Nr1h2/Nr1h4/Soat2 | 4 |
| GO:0006949 | BP | GO:0006949 | syncytium formation | 4/276 | 69/29008 | 0.004269623 | 0.146677122 | 0.129634292 | Adamts15/Adamts5/Fer1l5/Sbno2 | 4 |
| GO:0048332 | BP | GO:0048332 | mesoderm morphogenesis | 4/276 | 70/29008 | 0.004495087 | 0.146677122 | 0.129634292 | Chrd/Fgfr1/Foxf1/Hmga2 | 4 |
| GO:0033627 | BP | GO:0033627 | cell adhesion mediated by integrin | 4/276 | 74/29008 | 0.005476978 | 0.146677122 | 0.129634292 | Itga5/Itgbl1/P2ry12/Serpine1 | 4 |
| GO:2000379 | BP | GO:2000379 | positive regulation of reactive oxygen species metabolic process | 4/276 | 78/29008 | 0.006592667 | 0.1491879 | 0.131853335 | Ager/Cxcl1/Lcn2/Xdh | 4 |
| GO:0072089 | BP | GO:0072089 | stem cell proliferation | 4/276 | 83/29008 | 0.008186512 | 0.162786947 | 0.143872271 | Cxcl1/Fgfr1/Hmga2/Prg4 | 4 |
| GO:0043535 | BP | GO:0043535 | regulation of blood vessel endothelial cell migration | 4/276 | 93/29008 | 0.012087332 | 0.181049245 | 0.160012621 | Akt1/Fgfr1/Hmox1/Rhoj | 4 |
| GO:0002532 | BP | GO:0002532 | production of molecular mediator involved in inflammatory response | 4/276 | 94/29008 | 0.012532457 | 0.181049245 | 0.160012621 | Apod/Chid1/Per1/Serpine1 | 4 |
| GO:0055072 | BP | GO:0055072 | iron ion homeostasis | 4/276 | 94/29008 | 0.012532457 | 0.181049245 | 0.160012621 | Hmox1/Lcn2/Slc25a28/Slc39a14 | 4 |
| GO:0045576 | BP | GO:0045576 | mast cell activation | 4/276 | 95/29008 | 0.012987931 | 0.182767167 | 0.161530932 | Fcgr3/Foxf1/Gab2/Hmox1 | 4 |
| GO:0001570 | BP | GO:0001570 | vasculogenesis | 4/276 | 96/29008 | 0.013453833 | 0.182767167 | 0.161530932 | Adm/Fgfr1/Foxf1/Xdh | 4 |
| GO:0009408 | BP | GO:0009408 | response to heat | 4/276 | 96/29008 | 0.013453833 | 0.182767167 | 0.161530932 | Akt1/Asic3/Hmox1/Rasa3 | 4 |
| GO:0002028 | BP | GO:0002028 | regulation of sodium ion transport | 4/276 | 97/29008 | 0.01393024 | 0.182767167 | 0.161530932 | Akt1/Cxcl1/Fgf11/Per1 | 4 |
| GO:0021510 | BP | GO:0021510 | spinal cord development | 4/276 | 103/29008 | 0.017013319 | 0.200401203 | 0.17711602 | Adarb1/Akt1/Chrd/Scyl3 | 4 |
| GO:0032006 | BP | GO:0032006 | regulation of TOR signaling | 4/276 | 105/29008 | 0.018128199 | 0.206528782 | 0.182531618 | Ddit4/Kptn/Nprl2/Ros1 | 4 |
| GO:0045833 | BP | GO:0045833 | negative regulation of lipid metabolic process | 4/276 | 106/29008 | 0.018702266 | 0.207720809 | 0.18358514 | Akt1/Apod/Dkk3/Nr1h4 | 4 |
| GO:0048145 | BP | GO:0048145 | regulation of fibroblast proliferation | 4/276 | 108/29008 | 0.019883935 | 0.212964464 | 0.18821952 | Ager/Ifi30/Lig4/Serpine1 | 4 |
| GO:0046916 | BP | GO:0046916 | cellular transition metal ion homeostasis | 4/276 | 111/29008 | 0.021741064 | 0.220545717 | 0.194919886 | Hmox1/Lcn2/Mt1/Slc39a14 | 4 |
| GO:0048144 | BP | GO:0048144 | fibroblast proliferation | 4/276 | 111/29008 | 0.021741064 | 0.220545717 | 0.194919886 | Ager/Ifi30/Lig4/Serpine1 | 4 |
| GO:0060840 | BP | GO:0060840 | artery development | 4/276 | 112/29008 | 0.02238287 | 0.224244476 | 0.198188876 | Adamts6/Foxf1/Lox/Tbx2 | 4 |
| GO:0001892 | BP | GO:0001892 | embryonic placenta development | 4/276 | 114/29008 | 0.023700893 | 0.226242151 | 0.199954435 | Adm/Akt1/Cdx4/Ggnbp2 | 4 |
| GO:0030316 | BP | GO:0030316 | osteoclast differentiation | 4/276 | 116/29008 | 0.025065072 | 0.231905611 | 0.204959841 | Gab2/Inpp5d/Ninj1/Sbno2 | 4 |
| GO:0043534 | BP | GO:0043534 | blood vessel endothelial cell migration | 4/276 | 116/29008 | 0.025065072 | 0.231905611 | 0.204959841 | Akt1/Fgfr1/Hmox1/Rhoj | 4 |
| GO:0032963 | BP | GO:0032963 | collagen metabolic process | 4/276 | 119/29008 | 0.02719853 | 0.237235699 | 0.209670612 | Ager/Ctsk/Nr1h4/Serpine1 | 4 |
| GO:0001910 | BP | GO:0001910 | regulation of leukocyte mediated cytotoxicity | 4/276 | 120/29008 | 0.027933066 | 0.240973085 | 0.21297374 | Ager/Crtam/Cxcl1/Pvr | 4 |
| GO:1904019 | BP | GO:1904019 | epithelial cell apoptotic process | 4/276 | 120/29008 | 0.027933066 | 0.240973085 | 0.21297374 | Ager/Bcl2l1/Hmox1/Serpine1 | 4 |
| GO:0017015 | BP | GO:0017015 | regulation of transforming growth factor beta receptor signaling pathway | 4/276 | 121/29008 | 0.028679342 | 0.240973085 | 0.21297374 | Eid2/Lox/Lrg1/Wfikkn1 | 4 |
| GO:0002224 | BP | GO:0002224 | toll-like receptor signaling pathway | 4/276 | 124/29008 | 0.030988873 | 0.243990254 | 0.215640336 | Ninj1/Nr1h4/Ptpn22/Tlr13 | 4 |
| GO:0002688 | BP | GO:0002688 | regulation of leukocyte chemotaxis | 4/276 | 124/29008 | 0.030988873 | 0.243990254 | 0.215640336 | Ninj1/Rarres2/Serpine1/Vegfd | 4 |
| GO:1903844 | BP | GO:1903844 | regulation of cellular response to transforming growth factor beta stimulus | 4/276 | 124/29008 | 0.030988873 | 0.243990254 | 0.215640336 | Eid2/Lox/Lrg1/Wfikkn1 | 4 |
| GO:0008286 | BP | GO:0008286 | insulin receptor signaling pathway | 4/276 | 127/29008 | 0.033404922 | 0.253751943 | 0.224267787 | Akt1/Nr1h4/Rarres2/Slc39a14 | 4 |
| GO:0001952 | BP | GO:0001952 | regulation of cell-matrix adhesion | 4/276 | 128/29008 | 0.034234028 | 0.258231499 | 0.22822685 | Apod/Map4k4/Ninj1/Serpine1 | 4 |
| GO:0031929 | BP | GO:0031929 | TOR signaling | 4/276 | 128/29008 | 0.034234028 | 0.258231499 | 0.22822685 | Ddit4/Kptn/Nprl2/Ros1 | 4 |
| GO:0046822 | BP | GO:0046822 | regulation of nucleocytoplasmic transport | 4/276 | 129/29008 | 0.035075035 | 0.258548553 | 0.228507065 | Apod/Ifi27/Ptpn22/Riok2 | 4 |
| GO:0045727 | BP | GO:0045727 | positive regulation of translation | 4/276 | 131/29008 | 0.036792804 | 0.268046568 | 0.236901478 | Cirbp/Eif4g3/Pld1/Zcchc4 | 4 |
| GO:0003158 | BP | GO:0003158 | endothelium development | 4/276 | 133/29008 | 0.038558302 | 0.273628651 | 0.241834964 | Apold1/Lcn2/Msn/Xdh | 4 |
| GO:0010508 | BP | GO:0010508 | positive regulation of autophagy | 4/276 | 133/29008 | 0.038558302 | 0.273628651 | 0.241834964 | Ager/Hmox1/Nprl2/Ulk1 | 4 |
| GO:1905039 | BP | GO:1905039 | carboxylic acid transmembrane transport | 4/276 | 134/29008 | 0.039458964 | 0.274011178 | 0.242173044 | Acsl6/Akt1/Lrrc8a/Slc25a18 | 4 |
| GO:1903825 | BP | GO:1903825 | organic acid transmembrane transport | 4/276 | 135/29008 | 0.040371574 | 0.279150457 | 0.246715175 | Acsl6/Akt1/Lrrc8a/Slc25a18 | 4 |
| GO:0031341 | BP | GO:0031341 | regulation of cell killing | 4/276 | 139/29008 | 0.044141485 | 0.289740051 | 0.256074334 | Ager/Crtam/Cxcl1/Pvr | 4 |
| GO:0071887 | BP | GO:0071887 | leukocyte apoptotic process | 4/276 | 140/29008 | 0.045113816 | 0.293739052 | 0.25960868 | Akt1/Hsh2d/Lmbr1l/Plekho2 | 4 |
| GO:0050728 | BP | GO:0050728 | negative regulation of inflammatory response | 4/276 | 143/29008 | 0.048102352 | 0.303489528 | 0.268226221 | Cd200r1/Ctla2a/Foxf1/Nr1h4 | 4 |
| GO:0044853 | CC | GO:0044853 | plasma membrane raft | 4/268 | 135/28886 | 0.03736152 | 0.431208931 | 0.410516242 | Hmox1/Ms4a4a/P2ry12/Smpd2 | 4 |
| GO:0042974 | MF | GO:0042974 | retinoic acid receptor binding | 4/273 | 33/28438 | 0.000273028 | 0.095279381 | 0.087028735 | Asxl1/Lrif1/Nr1h2/Nr1h4 | 4 |
| GO:0008009 | MF | GO:0008009 | chemokine activity | 4/273 | 41/28438 | 0.000636229 | 0.095279381 | 0.087028735 | Ccl6/Ccl9/Cxcl1/Pf4 | 4 |
| GO:0005044 | MF | GO:0005044 | scavenger receptor activity | 4/273 | 43/28438 | 0.000763763 | 0.095279381 | 0.087028735 | Cd163/Hhipl1/Msr1/Prg4 | 4 |
| GO:0038024 | MF | GO:0038024 | cargo receptor activity | 4/273 | 75/28438 | 0.005922726 | 0.279512649 | 0.255308464 | Cd163/Hhipl1/Msr1/Prg4 | 4 |
| GO:0042379 | MF | GO:0042379 | chemokine receptor binding | 4/273 | 77/28438 | 0.006497077 | 0.279512649 | 0.255308464 | Ccl6/Ccl9/Cxcl1/Pf4 | 4 |
| GO:0008081 | MF | GO:0008081 | phosphoric diester hydrolase activity | 4/273 | 86/28438 | 0.009537036 | 0.279512649 | 0.255308464 | Hmox1/Pde7a/Pld1/Smpd2 | 4 |
| GO:0004222 | MF | GO:0004222 | metalloendopeptidase activity | 4/273 | 106/28438 | 0.019249738 | 0.282518209 | 0.258053759 | Adam11/Adamts15/Adamts5/Adamts6 | 4 |
| GO:0005496 | MF | GO:0005496 | steroid binding | 4/273 | 122/28438 | 0.030269155 | 0.306311797 | 0.27978696 | Apod/Nr1h4/Soat2/Ugt1a7c | 4 |
| GO:0015293 | MF | GO:0015293 | symporter activity | 4/273 | 143/28438 | 0.04939832 | 0.373481237 | 0.341139913 | Slc10a6/Slc20a1/Slc25a18/Slc39a14 | 4 |
| GO:0045869 | BP | GO:0045869 | negative regulation of single stranded viral RNA replication via double stranded DNA intermediate | 3/276 | 12/29008 | 0.0001759 | 0.043443702 | 0.038395856 | Hmga2/Morc2a/Resf1 | 3 |
| GO:0045091 | BP | GO:0045091 | regulation of single stranded viral RNA replication via double stranded DNA intermediate | 3/276 | 14/29008 | 0.000286958 | 0.046121376 | 0.040762403 | Hmga2/Morc2a/Resf1 | 3 |
| GO:2000402 | BP | GO:2000402 | negative regulation of lymphocyte migration | 3/276 | 14/29008 | 0.000286958 | 0.046121376 | 0.040762403 | Akt1/Apod/Cd200r1 | 3 |
| GO:0039692 | BP | GO:0039692 | single stranded viral RNA replication via double stranded DNA intermediate | 3/276 | 15/29008 | 0.000356178 | 0.046121376 | 0.040762403 | Hmga2/Morc2a/Resf1 | 3 |
| GO:0010763 | BP | GO:0010763 | positive regulation of fibroblast migration | 3/276 | 20/29008 | 0.00086154 | 0.079655537 | 0.070400134 | Ager/Akt1/Fgfr1 | 3 |
| GO:0071676 | BP | GO:0071676 | negative regulation of mononuclear cell migration | 3/276 | 21/29008 | 0.000998089 | 0.084995193 | 0.075119361 | Akt1/Apod/Cd200r1 | 3 |
| GO:0034755 | BP | GO:0034755 | iron ion transmembrane transport | 3/276 | 22/29008 | 0.001147591 | 0.088419149 | 0.078145478 | Lcn2/Slc25a28/Slc39a14 | 3 |
| GO:0048333 | BP | GO:0048333 | mesodermal cell differentiation | 3/276 | 23/29008 | 0.001310494 | 0.098622274 | 0.087163073 | Fgfr1/Foxf1/Hmga2 | 3 |
| GO:0046037 | BP | GO:0046037 | GMP metabolic process | 3/276 | 25/29008 | 0.001678219 | 0.114242269 | 0.100968137 | Gda/Impdh1/Xdh | 3 |
| GO:0006817 | BP | GO:0006817 | phosphate ion transport | 3/276 | 26/29008 | 0.001883859 | 0.117233975 | 0.103612228 | Fgfr1/Ros1/Slc20a1 | 3 |
| GO:0071276 | BP | GO:0071276 | cellular response to cadmium ion | 3/276 | 26/29008 | 0.001883859 | 0.117233975 | 0.103612228 | Akt1/Hmox1/Mt1 | 3 |
| GO:0071605 | BP | GO:0071605 | monocyte chemotactic protein-1 production | 3/276 | 28/29008 | 0.002340625 | 0.132327381 | 0.116951888 | Ager/Apod/Nr1h4 | 3 |
| GO:0071637 | BP | GO:0071637 | regulation of monocyte chemotactic protein-1 production | 3/276 | 28/29008 | 0.002340625 | 0.132327381 | 0.116951888 | Ager/Apod/Nr1h4 | 3 |
| GO:0060384 | BP | GO:0060384 | innervation | 3/276 | 31/29008 | 0.003144834 | 0.14033381 | 0.124028028 | Adarb1/Prkcg/Sema3a | 3 |
| GO:0039694 | BP | GO:0039694 | viral RNA genome replication | 3/276 | 32/29008 | 0.003445979 | 0.14033381 | 0.124028028 | Hmga2/Morc2a/Resf1 | 3 |
| GO:1902042 | BP | GO:1902042 | negative regulation of extrinsic apoptotic signaling pathway via death domain receptors | 3/276 | 32/29008 | 0.003445979 | 0.14033381 | 0.124028028 | Bcl2l1/Hmox1/Serpine1 | 3 |
| GO:0021846 | BP | GO:0021846 | cell proliferation in forebrain | 3/276 | 35/29008 | 0.00445285 | 0.146677122 | 0.129634292 | Fgfr1/Hmga2/Numbl | 3 |
| GO:0002053 | BP | GO:0002053 | positive regulation of mesenchymal cell proliferation | 3/276 | 36/29008 | 0.004823867 | 0.146677122 | 0.129634292 | Chrd/Fgfr1/Foxf1 | 3 |
| GO:0022617 | BP | GO:0022617 | extracellular matrix disassembly | 3/276 | 36/29008 | 0.004823867 | 0.146677122 | 0.129634292 | Adamts15/Adamts5/Tpsab1 | 3 |
| GO:0046329 | BP | GO:0046329 | negative regulation of JNK cascade | 3/276 | 37/29008 | 0.005213007 | 0.146677122 | 0.129634292 | Akt1/Per1/Ptpn22 | 3 |
| GO:0046686 | BP | GO:0046686 | response to cadmium ion | 3/276 | 38/29008 | 0.005620508 | 0.146677122 | 0.129634292 | Akt1/Hmox1/Mt1 | 3 |
| GO:0009167 | BP | GO:0009167 | purine ribonucleoside monophosphate metabolic process | 3/276 | 39/29008 | 0.006046594 | 0.1491879 | 0.131853335 | Gda/Impdh1/Xdh | 3 |
| GO:0019432 | BP | GO:0019432 | triglyceride biosynthetic process | 3/276 | 39/29008 | 0.006046594 | 0.1491879 | 0.131853335 | Acsl6/Nr1h2/Nr1h4 | 3 |
| GO:0043304 | BP | GO:0043304 | regulation of mast cell degranulation | 3/276 | 39/29008 | 0.006046594 | 0.1491879 | 0.131853335 | Foxf1/Gab2/Hmox1 | 3 |
| GO:0010762 | BP | GO:0010762 | regulation of fibroblast migration | 3/276 | 41/29008 | 0.006955365 | 0.154161386 | 0.136248938 | Ager/Akt1/Fgfr1 | 3 |
| GO:0033006 | BP | GO:0033006 | regulation of mast cell activation involved in immune response | 3/276 | 41/29008 | 0.006955365 | 0.154161386 | 0.136248938 | Foxf1/Gab2/Hmox1 | 3 |
| GO:0071827 | BP | GO:0071827 | plasma lipoprotein particle organization | 3/276 | 41/29008 | 0.006955365 | 0.154161386 | 0.136248938 | Nr1h2/Nr1h4/Soat2 | 3 |
| GO:0009126 | BP | GO:0009126 | purine nucleoside monophosphate metabolic process | 3/276 | 42/29008 | 0.007438445 | 0.160271819 | 0.141649383 | Gda/Impdh1/Xdh | 3 |
| GO:0010464 | BP | GO:0010464 | regulation of mesenchymal cell proliferation | 3/276 | 44/29008 | 0.008462894 | 0.162786947 | 0.143872271 | Chrd/Fgfr1/Foxf1 | 3 |
| GO:0031507 | BP | GO:0031507 | heterochromatin assembly | 3/276 | 44/29008 | 0.008462894 | 0.162786947 | 0.143872271 | Hmga2/Morc2a/Resf1 | 3 |
| GO:0032007 | BP | GO:0032007 | negative regulation of TOR signaling | 3/276 | 44/29008 | 0.008462894 | 0.162786947 | 0.143872271 | Ddit4/Kptn/Nprl2 | 3 |
| GO:0002686 | BP | GO:0002686 | negative regulation of leukocyte migration | 3/276 | 45/29008 | 0.009004593 | 0.162786947 | 0.143872271 | Akt1/Apod/Cd200r1 | 3 |
| GO:0034198 | BP | GO:0034198 | cellular response to amino acid starvation | 3/276 | 45/29008 | 0.009004593 | 0.162786947 | 0.143872271 | Kptn/Nprl2/Ulk1 | 3 |
| GO:0071825 | BP | GO:0071825 | protein-lipid complex subunit organization | 3/276 | 45/29008 | 0.009004593 | 0.162786947 | 0.143872271 | Nr1h2/Nr1h4/Soat2 | 3 |
| GO:1905521 | BP | GO:1905521 | regulation of macrophage migration | 3/276 | 45/29008 | 0.009004593 | 0.162786947 | 0.143872271 | Cd200r1/P2ry12/Rarres2 | 3 |
| GO:0032965 | BP | GO:0032965 | regulation of collagen biosynthetic process | 3/276 | 46/29008 | 0.009566144 | 0.16882604 | 0.149209665 | Ager/Nr1h4/Serpine1 | 3 |
| GO:0034142 | BP | GO:0034142 | toll-like receptor 4 signaling pathway | 3/276 | 46/29008 | 0.009566144 | 0.16882604 | 0.149209665 | Ninj1/Nr1h4/Ptpn22 | 3 |
| GO:0048246 | BP | GO:0048246 | macrophage chemotaxis | 3/276 | 48/29008 | 0.010749348 | 0.176063978 | 0.155606606 | Myo9b/Ninj1/Rarres2 | 3 |
| GO:0007157 | BP | GO:0007157 | heterophilic cell-cell adhesion via plasma membrane cell adhesion molecules | 3/276 | 49/29008 | 0.011371252 | 0.176063978 | 0.155606606 | Crtam/Itga5/Pvr | 3 |
| GO:0032873 | BP | GO:0032873 | negative regulation of stress-activated MAPK cascade | 3/276 | 49/29008 | 0.011371252 | 0.176063978 | 0.155606606 | Akt1/Per1/Ptpn22 | 3 |
| GO:0070303 | BP | GO:0070303 | negative regulation of stress-activated protein kinase signaling cascade | 3/276 | 49/29008 | 0.011371252 | 0.176063978 | 0.155606606 | Akt1/Per1/Ptpn22 | 3 |
| GO:1990928 | BP | GO:1990928 | response to amino acid starvation | 3/276 | 49/29008 | 0.011371252 | 0.176063978 | 0.155606606 | Kptn/Nprl2/Ulk1 | 3 |
| GO:0007520 | BP | GO:0007520 | myoblast fusion | 3/276 | 50/29008 | 0.012013507 | 0.181049245 | 0.160012621 | Adamts15/Adamts5/Fer1l5 | 3 |
| GO:0006289 | BP | GO:0006289 | nucleotide-excision repair | 3/276 | 52/29008 | 0.013359471 | 0.182767167 | 0.161530932 | Cetn2/Lig4/Slx4 | 3 |
| GO:0045814 | BP | GO:0045814 | negative regulation of gene expression, epigenetic | 3/276 | 52/29008 | 0.013359471 | 0.182767167 | 0.161530932 | Hmga2/Morc2a/Resf1 | 3 |
| GO:0046460 | BP | GO:0046460 | neutral lipid biosynthetic process | 3/276 | 52/29008 | 0.013359471 | 0.182767167 | 0.161530932 | Acsl6/Nr1h2/Nr1h4 | 3 |
| GO:0046463 | BP | GO:0046463 | acylglycerol biosynthetic process | 3/276 | 52/29008 | 0.013359471 | 0.182767167 | 0.161530932 | Acsl6/Nr1h2/Nr1h4 | 3 |
| GO:0072091 | BP | GO:0072091 | regulation of stem cell proliferation | 3/276 | 52/29008 | 0.013359471 | 0.182767167 | 0.161530932 | Cxcl1/Fgfr1/Hmga2 | 3 |
| GO:1902041 | BP | GO:1902041 | regulation of extrinsic apoptotic signaling pathway via death domain receptors | 3/276 | 52/29008 | 0.013359471 | 0.182767167 | 0.161530932 | Bcl2l1/Hmox1/Serpine1 | 3 |
| GO:0002042 | BP | GO:0002042 | cell migration involved in sprouting angiogenesis | 3/276 | 53/29008 | 0.014063357 | 0.182767167 | 0.161530932 | Akt1/Hmox1/Rhoj | 3 |
| GO:0010712 | BP | GO:0010712 | regulation of collagen metabolic process | 3/276 | 53/29008 | 0.014063357 | 0.182767167 | 0.161530932 | Ager/Nr1h4/Serpine1 | 3 |
| GO:0043392 | BP | GO:0043392 | negative regulation of DNA binding | 3/276 | 53/29008 | 0.014063357 | 0.182767167 | 0.161530932 | Hmga2/Hmox1/Wfikkn1 | 3 |
| GO:0070828 | BP | GO:0070828 | heterochromatin organization | 3/276 | 53/29008 | 0.014063357 | 0.182767167 | 0.161530932 | Hmga2/Morc2a/Resf1 | 3 |
| GO:0072132 | BP | GO:0072132 | mesenchyme morphogenesis | 3/276 | 53/29008 | 0.014063357 | 0.182767167 | 0.161530932 | Fgfr1/Foxf1/Tbx2 | 3 |
| GO:0009161 | BP | GO:0009161 | ribonucleoside monophosphate metabolic process | 3/276 | 54/29008 | 0.01478795 | 0.189896056 | 0.167831496 | Gda/Impdh1/Xdh | 3 |
| GO:0032964 | BP | GO:0032964 | collagen biosynthetic process | 3/276 | 55/29008 | 0.015533317 | 0.19333005 | 0.170866484 | Ager/Nr1h4/Serpine1 | 3 |
| GO:0010463 | BP | GO:0010463 | mesenchymal cell proliferation | 3/276 | 56/29008 | 0.016299517 | 0.199792568 | 0.176578104 | Chrd/Fgfr1/Foxf1 | 3 |
| GO:0010761 | BP | GO:0010761 | fibroblast migration | 3/276 | 58/29008 | 0.017894618 | 0.206528782 | 0.182531618 | Ager/Akt1/Fgfr1 | 3 |
| GO:0006826 | BP | GO:0006826 | iron ion transport | 3/276 | 59/29008 | 0.0187236 | 0.207720809 | 0.18358514 | Lcn2/Slc25a28/Slc39a14 | 3 |
| GO:1900015 | BP | GO:1900015 | regulation of cytokine production involved in inflammatory response | 3/276 | 59/29008 | 0.0187236 | 0.207720809 | 0.18358514 | Apod/Chid1/Per1 | 3 |
| GO:0033003 | BP | GO:0033003 | regulation of mast cell activation | 3/276 | 60/29008 | 0.019573576 | 0.212964464 | 0.18821952 | Foxf1/Gab2/Hmox1 | 3 |
| GO:0035924 | BP | GO:0035924 | cellular response to vascular endothelial growth factor stimulus | 3/276 | 60/29008 | 0.019573576 | 0.212964464 | 0.18821952 | Akt1/Vegfd/Xdh | 3 |
| GO:0031102 | BP | GO:0031102 | neuron projection regeneration | 3/276 | 61/29008 | 0.02044457 | 0.216204665 | 0.191083233 | Adm/Rtn4rl2/Ulk1 | 3 |
| GO:0043300 | BP | GO:0043300 | regulation of leukocyte degranulation | 3/276 | 61/29008 | 0.02044457 | 0.216204665 | 0.191083233 | Foxf1/Gab2/Hmox1 | 3 |
| GO:0002534 | BP | GO:0002534 | cytokine production involved in inflammatory response | 3/276 | 62/29008 | 0.021336596 | 0.220545717 | 0.194919886 | Apod/Chid1/Per1 | 3 |
| GO:0046622 | BP | GO:0046622 | positive regulation of organ growth | 3/276 | 62/29008 | 0.021336596 | 0.220545717 | 0.194919886 | Akt1/Fgfr1/Tbx2 | 3 |
| GO:0072678 | BP | GO:0072678 | T cell migration | 3/276 | 63/29008 | 0.022249662 | 0.223602198 | 0.197621226 | Apod/Cd200r1/Msn | 3 |
| GO:0001836 | BP | GO:0001836 | release of cytochrome c from mitochondria | 3/276 | 64/29008 | 0.023183772 | 0.226242151 | 0.199954435 | Akt1/Bcl2l1/Plaur | 3 |
| GO:0009123 | BP | GO:0009123 | nucleoside monophosphate metabolic process | 3/276 | 67/29008 | 0.026112277 | 0.237235699 | 0.209670612 | Gda/Impdh1/Xdh | 3 |
| GO:0090398 | BP | GO:0090398 | cellular senescence | 3/276 | 67/29008 | 0.026112277 | 0.237235699 | 0.209670612 | Hmga2/Smc5/Tbx2 | 3 |
| GO:0035904 | BP | GO:0035904 | aorta development | 3/276 | 68/29008 | 0.027130447 | 0.237235699 | 0.209670612 | Adamts6/Lox/Tbx2 | 3 |
| GO:0006879 | BP | GO:0006879 | cellular iron ion homeostasis | 3/276 | 69/29008 | 0.028169575 | 0.240973085 | 0.21297374 | Hmox1/Lcn2/Slc39a14 | 3 |
| GO:0043303 | BP | GO:0043303 | mast cell degranulation | 3/276 | 69/29008 | 0.028169575 | 0.240973085 | 0.21297374 | Foxf1/Gab2/Hmox1 | 3 |
| GO:0002448 | BP | GO:0002448 | mast cell mediated immunity | 3/276 | 70/29008 | 0.029229627 | 0.240973085 | 0.21297374 | Foxf1/Gab2/Hmox1 | 3 |
| GO:0048146 | BP | GO:0048146 | positive regulation of fibroblast proliferation | 3/276 | 70/29008 | 0.029229627 | 0.240973085 | 0.21297374 | Ager/Lig4/Serpine1 | 3 |
| GO:0050771 | BP | GO:0050771 | negative regulation of axonogenesis | 3/276 | 70/29008 | 0.029229627 | 0.240973085 | 0.21297374 | Sema3a/Sema6b/Ulk1 | 3 |
| GO:0071479 | BP | GO:0071479 | cellular response to ionizing radiation | 3/276 | 70/29008 | 0.029229627 | 0.240973085 | 0.21297374 | Bcl2l1/Hmga2/Lig4 | 3 |
| GO:0002279 | BP | GO:0002279 | mast cell activation involved in immune response | 3/276 | 71/29008 | 0.030310562 | 0.243990254 | 0.215640336 | Foxf1/Gab2/Hmox1 | 3 |
| GO:0007405 | BP | GO:0007405 | neuroblast proliferation | 3/276 | 72/29008 | 0.031412335 | 0.244940523 | 0.21648019 | Acsl6/Fgfr1/Numbl | 3 |
| GO:0010507 | BP | GO:0010507 | negative regulation of autophagy | 3/276 | 72/29008 | 0.031412335 | 0.244940523 | 0.21648019 | Akt1/Hmox1/Ptpn22 | 3 |
| GO:1905330 | BP | GO:1905330 | regulation of morphogenesis of an epithelium | 3/276 | 72/29008 | 0.031412335 | 0.244940523 | 0.21648019 | Fgfr1/Lcn2/Tbx2 | 3 |
| GO:0016575 | BP | GO:0016575 | histone deacetylation | 3/276 | 78/29008 | 0.038457393 | 0.273628651 | 0.241834964 | Jdp2/Per1/Prdm5 | 3 |
| GO:0038034 | BP | GO:0038034 | signal transduction in absence of ligand | 3/276 | 80/29008 | 0.040969589 | 0.279338487 | 0.246881357 | Bcl2l1/Fgfr1/Lcn2 | 3 |
| GO:0051145 | BP | GO:0051145 | smooth muscle cell differentiation | 3/276 | 80/29008 | 0.040969589 | 0.279338487 | 0.246881357 | Adm/Foxf1/Tbx2 | 3 |
| GO:0071347 | BP | GO:0071347 | cellular response to interleukin-1 | 3/276 | 80/29008 | 0.040969589 | 0.279338487 | 0.246881357 | Ccl6/Ccl9/Serpine1 | 3 |
| GO:0097192 | BP | GO:0097192 | extrinsic apoptotic signaling pathway in absence of ligand | 3/276 | 80/29008 | 0.040969589 | 0.279338487 | 0.246881357 | Bcl2l1/Fgfr1/Lcn2 | 3 |
| GO:0008625 | BP | GO:0008625 | extrinsic apoptotic signaling pathway via death domain receptors | 3/276 | 82/29008 | 0.043562628 | 0.287323622 | 0.253938677 | Bcl2l1/Hmox1/Serpine1 | 3 |
| GO:0034308 | BP | GO:0034308 | primary alcohol metabolic process | 3/276 | 82/29008 | 0.043562628 | 0.287323622 | 0.253938677 | Chka/Dkk3/Hao1 | 3 |
| GO:0043279 | BP | GO:0043279 | response to alkaloid | 3/276 | 82/29008 | 0.043562628 | 0.287323622 | 0.253938677 | Bcl2l1/Gnal/Prkcg | 3 |
| GO:0031397 | BP | GO:0031397 | negative regulation of protein ubiquitination | 3/276 | 83/29008 | 0.044889232 | 0.292866037 | 0.258837103 | Akt1/N4bp1/Prkcg | 3 |
| GO:0046785 | BP | GO:0046785 | microtubule polymerization | 3/276 | 83/29008 | 0.044889232 | 0.292866037 | 0.258837103 | Ccdc57/Clip3/Gda | 3 |
| GO:0050766 | BP | GO:0050766 | positive regulation of phagocytosis | 3/276 | 84/29008 | 0.046235762 | 0.296862948 | 0.262369602 | Ager/Fcgr3/Rab31 | 3 |
| GO:0061178 | BP | GO:0061178 | regulation of insulin secretion involved in cellular response to glucose stimulus | 3/276 | 84/29008 | 0.046235762 | 0.296862948 | 0.262369602 | Nr1h4/Pim3/Sidt2 | 3 |
| GO:0051881 | BP | GO:0051881 | regulation of mitochondrial membrane potential | 3/276 | 85/29008 | 0.047602115 | 0.303229221 | 0.267996159 | Akt1/Bcl2l1/Hsh2d | 3 |
| GO:0006354 | BP | GO:0006354 | DNA-templated transcription, elongation | 3/276 | 86/29008 | 0.048988185 | 0.306034296 | 0.270475305 | Ell/Pabpc1l/Zfp326 | 3 |
| GO:1990391 | CC | GO:1990391 | DNA repair complex | 3/268 | 35/28886 | 0.004150671 | 0.242964068 | 0.231304802 | Cetn2/Lig4/Slx4 | 3 |
| GO:0000791 | CC | GO:0000791 | euchromatin | 3/268 | 46/28886 | 0.008933932 | 0.254484318 | 0.242272222 | Nr1h4/Tbp/Trim24 | 3 |
| GO:0034358 | CC | GO:0034358 | plasma lipoprotein particle | 3/268 | 47/28886 | 0.009478668 | 0.254484318 | 0.242272222 | Hp/Msr1/Saa1 | 3 |
| GO:1990777 | CC | GO:1990777 | lipoprotein particle | 3/268 | 47/28886 | 0.009478668 | 0.254484318 | 0.242272222 | Hp/Msr1/Saa1 | 3 |
| GO:0032994 | CC | GO:0032994 | protein-lipid complex | 3/268 | 50/28886 | 0.011227249 | 0.254484318 | 0.242272222 | Hp/Msr1/Saa1 | 3 |
| GO:0031902 | CC | GO:0031902 | late endosome membrane | 3/268 | 70/28886 | 0.027409209 | 0.431208931 | 0.410516242 | Cd68/Ifitm2/Slc39a14 | 3 |
| GO:0005581 | CC | GO:0005581 | collagen trimer | 3/268 | 81/28886 | 0.039696874 | 0.431208931 | 0.410516242 | Fcna/Lox/Msr1 | 3 |
| GO:0035497 | MF | GO:0035497 | cAMP response element binding | 3/273 | 18/28438 | 0.000641696 | 0.095279381 | 0.087028735 | E4f1/Hmga2/Jdp2 | 3 |
| GO:0030547 | MF | GO:0030547 | signaling receptor inhibitor activity | 3/273 | 37/28438 | 0.005342445 | 0.279512649 | 0.255308464 | Dkk3/Ly6i/Wfikkn1 | 3 |
| GO:0042056 | MF | GO:0042056 | chemoattractant activity | 3/273 | 41/28438 | 0.007126332 | 0.279512649 | 0.255308464 | Ccl6/Ccl9/Vegfd | 3 |
| GO:0043394 | MF | GO:0043394 | proteoglycan binding | 3/273 | 55/28438 | 0.015901717 | 0.282518209 | 0.258053759 | Chrd/Ctsk/Fcna | 3 |
| GO:0001098 | MF | GO:0001098 | basal transcription machinery binding | 3/273 | 58/28438 | 0.01831574 | 0.282518209 | 0.258053759 | Scaf1/Tbp/Zfp326 | 3 |
| GO:0001099 | MF | GO:0001099 | basal RNA polymerase II transcription machinery binding | 3/273 | 58/28438 | 0.01831574 | 0.282518209 | 0.258053759 | Scaf1/Tbp/Zfp326 | 3 |
| GO:0050840 | MF | GO:0050840 | extracellular matrix binding | 3/273 | 58/28438 | 0.01831574 | 0.282518209 | 0.258053759 | Adamts15/Adamts5/Spock2 | 3 |
| GO:0003725 | MF | GO:0003725 | double-stranded RNA binding | 3/273 | 84/28438 | 0.047251583 | 0.373481237 | 0.341139913 | Adarb1/Msn/Sidt2 | 3 |
| GO:0002420 | BP | GO:0002420 | natural killer cell mediated cytotoxicity directed against tumor cell target | 2/276 | 10/29008 | 0.003859613 | 0.14033381 | 0.124028028 | Crtam/Pvr | 2 |
| GO:0002858 | BP | GO:0002858 | regulation of natural killer cell mediated cytotoxicity directed against tumor cell target | 2/276 | 10/29008 | 0.003859613 | 0.14033381 | 0.124028028 | Crtam/Pvr | 2 |
| GO:0046055 | BP | GO:0046055 | dGMP catabolic process | 2/276 | 10/29008 | 0.003859613 | 0.14033381 | 0.124028028 | Gda/Xdh | 2 |
| GO:2000234 | BP | GO:2000234 | positive regulation of rRNA processing | 2/276 | 10/29008 | 0.003859613 | 0.14033381 | 0.124028028 | Riok1/Riok2 | 2 |
| GO:0002423 | BP | GO:0002423 | natural killer cell mediated immune response to tumor cell | 2/276 | 11/29008 | 0.004687781 | 0.146677122 | 0.129634292 | Crtam/Pvr | 2 |
| GO:0002855 | BP | GO:0002855 | regulation of natural killer cell mediated immune response to tumor cell | 2/276 | 11/29008 | 0.004687781 | 0.146677122 | 0.129634292 | Crtam/Pvr | 2 |
| GO:0002887 | BP | GO:0002887 | negative regulation of myeloid leukocyte mediated immunity | 2/276 | 11/29008 | 0.004687781 | 0.146677122 | 0.129634292 | Foxf1/Hmox1 | 2 |
| GO:0009172 | BP | GO:0009172 | purine deoxyribonucleoside monophosphate catabolic process | 2/276 | 11/29008 | 0.004687781 | 0.146677122 | 0.129634292 | Gda/Xdh | 2 |
| GO:0015810 | BP | GO:0015810 | aspartate transmembrane transport | 2/276 | 11/29008 | 0.004687781 | 0.146677122 | 0.129634292 | Lrrc8a/Slc25a18 | 2 |
| GO:0034145 | BP | GO:0034145 | positive regulation of toll-like receptor 4 signaling pathway | 2/276 | 11/29008 | 0.004687781 | 0.146677122 | 0.129634292 | Ninj1/Ptpn22 | 2 |
| GO:0090309 | BP | GO:0090309 | positive regulation of DNA methylation-dependent heterochromatin assembly | 2/276 | 11/29008 | 0.004687781 | 0.146677122 | 0.129634292 | Morc2a/Resf1 | 2 |
| GO:0140052 | BP | GO:0140052 | cellular response to oxidised low-density lipoprotein particle stimulus | 2/276 | 11/29008 | 0.004687781 | 0.146677122 | 0.129634292 | Akt1/Cd68 | 2 |
| GO:0002246 | BP | GO:0002246 | wound healing involved in inflammatory response | 2/276 | 12/29008 | 0.005590157 | 0.146677122 | 0.129634292 | Ager/Hmox1 | 2 |
| GO:0006152 | BP | GO:0006152 | purine nucleoside catabolic process | 2/276 | 12/29008 | 0.005590157 | 0.146677122 | 0.129634292 | Gda/Xdh | 2 |
| GO:0015911 | BP | GO:0015911 | long-chain fatty acid import across plasma membrane | 2/276 | 12/29008 | 0.005590157 | 0.146677122 | 0.129634292 | Acsl6/Akt1 | 2 |
| GO:0021561 | BP | GO:0021561 | facial nerve development | 2/276 | 12/29008 | 0.005590157 | 0.146677122 | 0.129634292 | Adarb1/Sema3a | 2 |
| GO:0021610 | BP | GO:0021610 | facial nerve morphogenesis | 2/276 | 12/29008 | 0.005590157 | 0.146677122 | 0.129634292 | Adarb1/Sema3a | 2 |
| GO:0031453 | BP | GO:0031453 | positive regulation of heterochromatin assembly | 2/276 | 12/29008 | 0.005590157 | 0.146677122 | 0.129634292 | Morc2a/Resf1 | 2 |
| GO:0033212 | BP | GO:0033212 | iron import into cell | 2/276 | 12/29008 | 0.005590157 | 0.146677122 | 0.129634292 | Lcn2/Slc39a14 | 2 |
| GO:0033275 | BP | GO:0033275 | actin-myosin filament sliding | 2/276 | 12/29008 | 0.005590157 | 0.146677122 | 0.129634292 | Myo9b/Tnnt2 | 2 |
| GO:0043301 | BP | GO:0043301 | negative regulation of leukocyte degranulation | 2/276 | 12/29008 | 0.005590157 | 0.146677122 | 0.129634292 | Foxf1/Hmox1 | 2 |
| GO:0120263 | BP | GO:0120263 | positive regulation of heterochromatin organization | 2/276 | 12/29008 | 0.005590157 | 0.146677122 | 0.129634292 | Morc2a/Resf1 | 2 |
| GO:0000255 | BP | GO:0000255 | allantoin metabolic process | 2/276 | 13/29008 | 0.006565265 | 0.1491879 | 0.131853335 | Gda/Xdh | 2 |
| GO:0009120 | BP | GO:0009120 | deoxyribonucleoside metabolic process | 2/276 | 13/29008 | 0.006565265 | 0.1491879 | 0.131853335 | Gda/Xdh | 2 |
| GO:0033004 | BP | GO:0033004 | negative regulation of mast cell activation | 2/276 | 13/29008 | 0.006565265 | 0.1491879 | 0.131853335 | Foxf1/Hmox1 | 2 |
| GO:0042590 | BP | GO:0042590 | antigen processing and presentation of exogenous peptide antigen via MHC class I | 2/276 | 13/29008 | 0.006565265 | 0.1491879 | 0.131853335 | Fcgr3/Ifi30 | 2 |
| GO:0046054 | BP | GO:0046054 | dGMP metabolic process | 2/276 | 13/29008 | 0.006565265 | 0.1491879 | 0.131853335 | Gda/Xdh | 2 |
| GO:0051549 | BP | GO:0051549 | positive regulation of keratinocyte migration | 2/276 | 13/29008 | 0.006565265 | 0.1491879 | 0.131853335 | Map4k4/Serpine1 | 2 |
| GO:1905269 | BP | GO:1905269 | positive regulation of chromatin organization | 2/276 | 13/29008 | 0.006565265 | 0.1491879 | 0.131853335 | Morc2a/Resf1 | 2 |
| GO:0090308 | BP | GO:0090308 | regulation of DNA methylation-dependent heterochromatin assembly | 2/276 | 14/29008 | 0.00761165 | 0.160271819 | 0.141649383 | Morc2a/Resf1 | 2 |
| GO:0098840 | BP | GO:0098840 | protein transport along microtubule | 2/276 | 14/29008 | 0.00761165 | 0.160271819 | 0.141649383 | Clip3/Kif5a | 2 |
| GO:0099118 | BP | GO:0099118 | microtubule-based protein transport | 2/276 | 14/29008 | 0.00761165 | 0.160271819 | 0.141649383 | Clip3/Kif5a | 2 |
| GO:0006570 | BP | GO:0006570 | tyrosine metabolic process | 2/276 | 15/29008 | 0.008727876 | 0.162786947 | 0.143872271 | Hgd/Iyd | 2 |
| GO:0009155 | BP | GO:0009155 | purine deoxyribonucleotide catabolic process | 2/276 | 15/29008 | 0.008727876 | 0.162786947 | 0.143872271 | Gda/Xdh | 2 |
| GO:0009169 | BP | GO:0009169 | purine ribonucleoside monophosphate catabolic process | 2/276 | 15/29008 | 0.008727876 | 0.162786947 | 0.143872271 | Gda/Xdh | 2 |
| GO:0009170 | BP | GO:0009170 | purine deoxyribonucleoside monophosphate metabolic process | 2/276 | 15/29008 | 0.008727876 | 0.162786947 | 0.143872271 | Gda/Xdh | 2 |
| GO:0050930 | BP | GO:0050930 | induction of positive chemotaxis | 2/276 | 15/29008 | 0.008727876 | 0.162786947 | 0.143872271 | Ager/Vegfd | 2 |
| GO:0051547 | BP | GO:0051547 | regulation of keratinocyte migration | 2/276 | 15/29008 | 0.008727876 | 0.162786947 | 0.143872271 | Map4k4/Serpine1 | 2 |
| GO:2000811 | BP | GO:2000811 | negative regulation of anoikis | 2/276 | 15/29008 | 0.008727876 | 0.162786947 | 0.143872271 | Bcl2l1/Itga5 | 2 |
| GO:0009128 | BP | GO:0009128 | purine nucleoside monophosphate catabolic process | 2/276 | 16/29008 | 0.00991253 | 0.16882604 | 0.149209665 | Gda/Xdh | 2 |
| GO:0034162 | BP | GO:0034162 | toll-like receptor 9 signaling pathway | 2/276 | 16/29008 | 0.00991253 | 0.16882604 | 0.149209665 | Nr1h4/Ptpn22 | 2 |
| GO:0055057 | BP | GO:0055057 | neuroblast division | 2/276 | 16/29008 | 0.00991253 | 0.16882604 | 0.149209665 | Fgfr1/Numbl | 2 |
| GO:1902001 | BP | GO:1902001 | fatty acid transmembrane transport | 2/276 | 16/29008 | 0.00991253 | 0.16882604 | 0.149209665 | Acsl6/Akt1 | 2 |
| GO:2000232 | BP | GO:2000232 | regulation of rRNA processing | 2/276 | 16/29008 | 0.00991253 | 0.16882604 | 0.149209665 | Riok1/Riok2 | 2 |
| GO:2001212 | BP | GO:2001212 | regulation of vasculogenesis | 2/276 | 16/29008 | 0.00991253 | 0.16882604 | 0.149209665 | Adm/Xdh | 2 |
| GO:0010713 | BP | GO:0010713 | negative regulation of collagen metabolic process | 2/276 | 17/29008 | 0.011164219 | 0.176063978 | 0.155606606 | Ager/Nr1h4 | 2 |
| GO:0031445 | BP | GO:0031445 | regulation of heterochromatin assembly | 2/276 | 17/29008 | 0.011164219 | 0.176063978 | 0.155606606 | Morc2a/Resf1 | 2 |
| GO:0032966 | BP | GO:0032966 | negative regulation of collagen biosynthetic process | 2/276 | 17/29008 | 0.011164219 | 0.176063978 | 0.155606606 | Ager/Nr1h4 | 2 |
| GO:0048368 | BP | GO:0048368 | lateral mesoderm development | 2/276 | 17/29008 | 0.011164219 | 0.176063978 | 0.155606606 | Fgfr1/Foxf1 | 2 |
| GO:0060100 | BP | GO:0060100 | positive regulation of phagocytosis, engulfment | 2/276 | 17/29008 | 0.011164219 | 0.176063978 | 0.155606606 | Ager/Rab31 | 2 |
| GO:0071243 | BP | GO:0071243 | cellular response to arsenic-containing substance | 2/276 | 17/29008 | 0.011164219 | 0.176063978 | 0.155606606 | Hmox1/Zfand2a | 2 |
| GO:0120261 | BP | GO:0120261 | regulation of heterochromatin organization | 2/276 | 17/29008 | 0.011164219 | 0.176063978 | 0.155606606 | Morc2a/Resf1 | 2 |
| GO:1900452 | BP | GO:1900452 | regulation of long-term synaptic depression | 2/276 | 17/29008 | 0.011164219 | 0.176063978 | 0.155606606 | Ager/Bcl2l1 | 2 |
| GO:1905155 | BP | GO:1905155 | positive regulation of membrane invagination | 2/276 | 17/29008 | 0.011164219 | 0.176063978 | 0.155606606 | Ager/Rab31 | 2 |
| GO:0021783 | BP | GO:0021783 | preganglionic parasympathetic fiber development | 2/276 | 18/29008 | 0.012481569 | 0.181049245 | 0.160012621 | Adarb1/Sema3a | 2 |
| GO:2001185 | BP | GO:2001185 | regulation of CD8-positive, alpha-beta T cell activation | 2/276 | 18/29008 | 0.012481569 | 0.181049245 | 0.160012621 | Crtam/Ptpn22 | 2 |
| GO:0044539 | BP | GO:0044539 | long-chain fatty acid import into cell | 2/276 | 19/29008 | 0.013863227 | 0.182767167 | 0.161530932 | Acsl6/Akt1 | 2 |
| GO:0045198 | BP | GO:0045198 | establishment of epithelial cell apical/basal polarity | 2/276 | 19/29008 | 0.013863227 | 0.182767167 | 0.161530932 | Foxf1/Msn | 2 |
| GO:0060712 | BP | GO:0060712 | spongiotrophoblast layer development | 2/276 | 19/29008 | 0.013863227 | 0.182767167 | 0.161530932 | Adm/Akt1 | 2 |
| GO:0140354 | BP | GO:0140354 | lipid import into cell | 2/276 | 19/29008 | 0.013863227 | 0.182767167 | 0.161530932 | Acsl6/Akt1 | 2 |
| GO:0006144 | BP | GO:0006144 | purine nucleobase metabolic process | 2/276 | 20/29008 | 0.015307859 | 0.191958105 | 0.169653949 | Gda/Xdh | 2 |
| GO:0009159 | BP | GO:0009159 | deoxyribonucleoside monophosphate catabolic process | 2/276 | 20/29008 | 0.015307859 | 0.191958105 | 0.169653949 | Gda/Xdh | 2 |
| GO:0051546 | BP | GO:0051546 | keratinocyte migration | 2/276 | 20/29008 | 0.015307859 | 0.191958105 | 0.169653949 | Map4k4/Serpine1 | 2 |
| GO:0060099 | BP | GO:0060099 | regulation of phagocytosis, engulfment | 2/276 | 20/29008 | 0.015307859 | 0.191958105 | 0.169653949 | Ager/Rab31 | 2 |
| GO:0002281 | BP | GO:0002281 | macrophage activation involved in immune response | 2/276 | 21/29008 | 0.01681415 | 0.200038936 | 0.176795846 | Fer1l5/Sbno2 | 2 |
| GO:0002836 | BP | GO:0002836 | positive regulation of response to tumor cell | 2/276 | 21/29008 | 0.01681415 | 0.200038936 | 0.176795846 | Crtam/Pvr | 2 |
| GO:0002839 | BP | GO:0002839 | positive regulation of immune response to tumor cell | 2/276 | 21/29008 | 0.01681415 | 0.200038936 | 0.176795846 | Crtam/Pvr | 2 |
| GO:0006346 | BP | GO:0006346 | DNA methylation-dependent heterochromatin assembly | 2/276 | 21/29008 | 0.01681415 | 0.200038936 | 0.176795846 | Morc2a/Resf1 | 2 |
| GO:0048486 | BP | GO:0048486 | parasympathetic nervous system development | 2/276 | 21/29008 | 0.01681415 | 0.200038936 | 0.176795846 | Adarb1/Sema3a | 2 |
| GO:0090050 | BP | GO:0090050 | positive regulation of cell migration involved in sprouting angiogenesis | 2/276 | 21/29008 | 0.01681415 | 0.200038936 | 0.176795846 | Hmox1/Rhoj | 2 |
| GO:0090594 | BP | GO:0090594 | inflammatory response to wounding | 2/276 | 21/29008 | 0.01681415 | 0.200038936 | 0.176795846 | Ager/Hmox1 | 2 |
| GO:1905153 | BP | GO:1905153 | regulation of membrane invagination | 2/276 | 21/29008 | 0.01681415 | 0.200038936 | 0.176795846 | Ager/Rab31 | 2 |
| GO:0009158 | BP | GO:0009158 | ribonucleoside monophosphate catabolic process | 2/276 | 22/29008 | 0.018380806 | 0.206528782 | 0.182531618 | Gda/Xdh | 2 |
| GO:0009164 | BP | GO:0009164 | nucleoside catabolic process | 2/276 | 22/29008 | 0.018380806 | 0.206528782 | 0.182531618 | Gda/Xdh | 2 |
| GO:0010885 | BP | GO:0010885 | regulation of cholesterol storage | 2/276 | 22/29008 | 0.018380806 | 0.206528782 | 0.182531618 | Msr1/Nr1h2 | 2 |
| GO:0034143 | BP | GO:0034143 | regulation of toll-like receptor 4 signaling pathway | 2/276 | 22/29008 | 0.018380806 | 0.206528782 | 0.182531618 | Ninj1/Ptpn22 | 2 |
| GO:0034377 | BP | GO:0034377 | plasma lipoprotein particle assembly | 2/276 | 22/29008 | 0.018380806 | 0.206528782 | 0.182531618 | Nr1h2/Soat2 | 2 |
| GO:2000209 | BP | GO:2000209 | regulation of anoikis | 2/276 | 22/29008 | 0.018380806 | 0.206528782 | 0.182531618 | Bcl2l1/Itga5 | 2 |
| GO:0006654 | BP | GO:0006654 | phosphatidic acid biosynthetic process | 2/276 | 23/29008 | 0.02000655 | 0.212964464 | 0.18821952 | Nr1h4/Pld1 | 2 |
| GO:0007190 | BP | GO:0007190 | activation of adenylate cyclase activity | 2/276 | 23/29008 | 0.02000655 | 0.212964464 | 0.18821952 | Acr/Gnal | 2 |
| GO:0009151 | BP | GO:0009151 | purine deoxyribonucleotide metabolic process | 2/276 | 23/29008 | 0.02000655 | 0.212964464 | 0.18821952 | Gda/Xdh | 2 |
| GO:0035089 | BP | GO:0035089 | establishment of apical/basal cell polarity | 2/276 | 23/29008 | 0.02000655 | 0.212964464 | 0.18821952 | Foxf1/Msn | 2 |
| GO:0046426 | BP | GO:0046426 | negative regulation of receptor signaling pathway via JAK-STAT | 2/276 | 23/29008 | 0.02000655 | 0.212964464 | 0.18821952 | Ggnbp2/Hmga2 | 2 |
| GO:1900016 | BP | GO:1900016 | negative regulation of cytokine production involved in inflammatory response | 2/276 | 23/29008 | 0.02000655 | 0.212964464 | 0.18821952 | Apod/Chid1 | 2 |
| GO:0010866 | BP | GO:0010866 | regulation of triglyceride biosynthetic process | 2/276 | 24/29008 | 0.021690125 | 0.220545717 | 0.194919886 | Nr1h2/Nr1h4 | 2 |
| GO:0010878 | BP | GO:0010878 | cholesterol storage | 2/276 | 24/29008 | 0.021690125 | 0.220545717 | 0.194919886 | Msr1/Nr1h2 | 2 |
| GO:0018230 | BP | GO:0018230 | peptidyl-L-cysteine S-palmitoylation | 2/276 | 24/29008 | 0.021690125 | 0.220545717 | 0.194919886 | Clip3/Zdhhc14 | 2 |
| GO:0018231 | BP | GO:0018231 | peptidyl-S-diacylglycerol-L-cysteine biosynthetic process from peptidyl-cysteine | 2/276 | 24/29008 | 0.021690125 | 0.220545717 | 0.194919886 | Clip3/Zdhhc14 | 2 |
| GO:0051123 | BP | GO:0051123 | RNA polymerase II preinitiation complex assembly | 2/276 | 24/29008 | 0.021690125 | 0.220545717 | 0.194919886 | Taf7l/Tbp | 2 |
| GO:0009125 | BP | GO:0009125 | nucleoside monophosphate catabolic process | 2/276 | 25/29008 | 0.023430291 | 0.226242151 | 0.199954435 | Gda/Xdh | 2 |
| GO:0010640 | BP | GO:0010640 | regulation of platelet-derived growth factor receptor signaling pathway | 2/276 | 25/29008 | 0.023430291 | 0.226242151 | 0.199954435 | Apod/Lox | 2 |
| GO:0010884 | BP | GO:0010884 | positive regulation of lipid storage | 2/276 | 25/29008 | 0.023430291 | 0.226242151 | 0.199954435 | Msr1/Nr1h2 | 2 |
| GO:0061162 | BP | GO:0061162 | establishment of monopolar cell polarity | 2/276 | 25/29008 | 0.023430291 | 0.226242151 | 0.199954435 | Foxf1/Msn | 2 |
| GO:0097164 | BP | GO:0097164 | ammonium ion metabolic process | 2/276 | 25/29008 | 0.023430291 | 0.226242151 | 0.199954435 | Chka/Hdc | 2 |
| GO:1903306 | BP | GO:1903306 | negative regulation of regulated secretory pathway | 2/276 | 25/29008 | 0.023430291 | 0.226242151 | 0.199954435 | Foxf1/Hmox1 | 2 |
| GO:1904893 | BP | GO:1904893 | negative regulation of receptor signaling pathway via STAT | 2/276 | 25/29008 | 0.023430291 | 0.226242151 | 0.199954435 | Ggnbp2/Hmga2 | 2 |
| GO:0030859 | BP | GO:0030859 | polarized epithelial cell differentiation | 2/276 | 26/29008 | 0.025225827 | 0.231905611 | 0.204959841 | Foxf1/Msn | 2 |
| GO:0034656 | BP | GO:0034656 | nucleobase-containing small molecule catabolic process | 2/276 | 26/29008 | 0.025225827 | 0.231905611 | 0.204959841 | Gda/Xdh | 2 |
| GO:0046386 | BP | GO:0046386 | deoxyribose phosphate catabolic process | 2/276 | 26/29008 | 0.025225827 | 0.231905611 | 0.204959841 | Gda/Xdh | 2 |
| GO:0046685 | BP | GO:0046685 | response to arsenic-containing substance | 2/276 | 26/29008 | 0.025225827 | 0.231905611 | 0.204959841 | Hmox1/Zfand2a | 2 |
| GO:0050927 | BP | GO:0050927 | positive regulation of positive chemotaxis | 2/276 | 26/29008 | 0.025225827 | 0.231905611 | 0.204959841 | Ager/Vegfd | 2 |
| GO:0061339 | BP | GO:0061339 | establishment or maintenance of monopolar cell polarity | 2/276 | 26/29008 | 0.025225827 | 0.231905611 | 0.204959841 | Foxf1/Msn | 2 |
| GO:0065005 | BP | GO:0065005 | protein-lipid complex assembly | 2/276 | 26/29008 | 0.025225827 | 0.231905611 | 0.204959841 | Nr1h2/Soat2 | 2 |
| GO:0006376 | BP | GO:0006376 | mRNA splice site selection | 2/276 | 27/29008 | 0.02707553 | 0.237235699 | 0.209670612 | Ptbp2/Sfswap | 2 |
| GO:0009264 | BP | GO:0009264 | deoxyribonucleotide catabolic process | 2/276 | 27/29008 | 0.02707553 | 0.237235699 | 0.209670612 | Gda/Xdh | 2 |
| GO:0010165 | BP | GO:0010165 | response to X-ray | 2/276 | 27/29008 | 0.02707553 | 0.237235699 | 0.209670612 | Hmga2/Lig4 | 2 |
| GO:0015740 | BP | GO:0015740 | C4-dicarboxylate transport | 2/276 | 27/29008 | 0.02707553 | 0.237235699 | 0.209670612 | Lrrc8a/Slc25a18 | 2 |
| GO:0031670 | BP | GO:0031670 | cellular response to nutrient | 2/276 | 27/29008 | 0.02707553 | 0.237235699 | 0.209670612 | Nr1h4/Trim24 | 2 |
| GO:0046473 | BP | GO:0046473 | phosphatidic acid metabolic process | 2/276 | 27/29008 | 0.02707553 | 0.237235699 | 0.209670612 | Nr1h4/Pld1 | 2 |
| GO:0048843 | BP | GO:0048843 | negative regulation of axon extension involved in axon guidance | 2/276 | 27/29008 | 0.02707553 | 0.237235699 | 0.209670612 | Sema3a/Sema6b | 2 |
| GO:0050926 | BP | GO:0050926 | regulation of positive chemotaxis | 2/276 | 27/29008 | 0.02707553 | 0.237235699 | 0.209670612 | Ager/Vegfd | 2 |
| GO:0060716 | BP | GO:0060716 | labyrinthine layer blood vessel development | 2/276 | 27/29008 | 0.02707553 | 0.237235699 | 0.209670612 | Akt1/Ggnbp2 | 2 |
| GO:0006882 | BP | GO:0006882 | cellular zinc ion homeostasis | 2/276 | 28/29008 | 0.028978215 | 0.240973085 | 0.21297374 | Mt1/Slc39a14 | 2 |
| GO:0010955 | BP | GO:0010955 | negative regulation of protein processing | 2/276 | 28/29008 | 0.028978215 | 0.240973085 | 0.21297374 | Ctla2a/Serpine1 | 2 |
| GO:0032104 | BP | GO:0032104 | regulation of response to extracellular stimulus | 2/276 | 28/29008 | 0.028978215 | 0.240973085 | 0.21297374 | Prkcg/Trim24 | 2 |
| GO:0032107 | BP | GO:0032107 | regulation of response to nutrient levels | 2/276 | 28/29008 | 0.028978215 | 0.240973085 | 0.21297374 | Prkcg/Trim24 | 2 |
| GO:0032928 | BP | GO:0032928 | regulation of superoxide anion generation | 2/276 | 28/29008 | 0.028978215 | 0.240973085 | 0.21297374 | Akt1/Cxcl1 | 2 |
| GO:0034104 | BP | GO:0034104 | negative regulation of tissue remodeling | 2/276 | 28/29008 | 0.028978215 | 0.240973085 | 0.21297374 | Ager/Inpp5d | 2 |
| GO:0060445 | BP | GO:0060445 | branching involved in salivary gland morphogenesis | 2/276 | 28/29008 | 0.028978215 | 0.240973085 | 0.21297374 | Fgfr1/Sema3a | 2 |
| GO:1900745 | BP | GO:1900745 | positive regulation of p38MAPK cascade | 2/276 | 28/29008 | 0.028978215 | 0.240973085 | 0.21297374 | Ager/Xdh | 2 |
| GO:1903318 | BP | GO:1903318 | negative regulation of protein maturation | 2/276 | 28/29008 | 0.028978215 | 0.240973085 | 0.21297374 | Ctla2a/Serpine1 | 2 |
| GO:0002834 | BP | GO:0002834 | regulation of response to tumor cell | 2/276 | 29/29008 | 0.030932714 | 0.243990254 | 0.215640336 | Crtam/Pvr | 2 |
| GO:0002837 | BP | GO:0002837 | regulation of immune response to tumor cell | 2/276 | 29/29008 | 0.030932714 | 0.243990254 | 0.215640336 | Crtam/Pvr | 2 |
| GO:0002888 | BP | GO:0002888 | positive regulation of myeloid leukocyte mediated immunity | 2/276 | 29/29008 | 0.030932714 | 0.243990254 | 0.215640336 | Cxcl1/Fcgr3 | 2 |
| GO:0016556 | BP | GO:0016556 | mRNA modification | 2/276 | 29/29008 | 0.030932714 | 0.243990254 | 0.215640336 | Adarb1/Pus7 | 2 |
| GO:0033119 | BP | GO:0033119 | negative regulation of RNA splicing | 2/276 | 29/29008 | 0.030932714 | 0.243990254 | 0.215640336 | Ptbp2/Sfswap | 2 |
| GO:0035987 | BP | GO:0035987 | endodermal cell differentiation | 2/276 | 29/29008 | 0.030932714 | 0.243990254 | 0.215640336 | Hmga2/Itga5 | 2 |
| GO:0048384 | BP | GO:0048384 | retinoic acid receptor signaling pathway | 2/276 | 29/29008 | 0.030932714 | 0.243990254 | 0.215640336 | Asxl1/Nr1h2 | 2 |
| GO:0055069 | BP | GO:0055069 | zinc ion homeostasis | 2/276 | 29/29008 | 0.030932714 | 0.243990254 | 0.215640336 | Mt1/Slc39a14 | 2 |
| GO:0060045 | BP | GO:0060045 | positive regulation of cardiac muscle cell proliferation | 2/276 | 29/29008 | 0.030932714 | 0.243990254 | 0.215640336 | Fgfr1/Tbx2 | 2 |
| GO:0070897 | BP | GO:0070897 | transcription preinitiation complex assembly | 2/276 | 29/29008 | 0.030932714 | 0.243990254 | 0.215640336 | Taf7l/Tbp | 2 |
| GO:0006929 | BP | GO:0006929 | substrate-dependent cell migration | 2/276 | 30/29008 | 0.032937876 | 0.251441119 | 0.222225463 | Fgfr1/P2ry12 | 2 |
| GO:0021602 | BP | GO:0021602 | cranial nerve morphogenesis | 2/276 | 30/29008 | 0.032937876 | 0.251441119 | 0.222225463 | Adarb1/Sema3a | 2 |
| GO:0032682 | BP | GO:0032682 | negative regulation of chemokine production | 2/276 | 30/29008 | 0.032937876 | 0.251441119 | 0.222225463 | Apod/Nr1h4 | 2 |
| GO:0034123 | BP | GO:0034123 | positive regulation of toll-like receptor signaling pathway | 2/276 | 30/29008 | 0.032937876 | 0.251441119 | 0.222225463 | Ninj1/Ptpn22 | 2 |
| GO:0090025 | BP | GO:0090025 | regulation of monocyte chemotaxis | 2/276 | 30/29008 | 0.032937876 | 0.251441119 | 0.222225463 | Ninj1/Serpine1 | 2 |
| GO:1901658 | BP | GO:1901658 | glycosyl compound catabolic process | 2/276 | 30/29008 | 0.032937876 | 0.251441119 | 0.222225463 | Gda/Xdh | 2 |
| GO:0002418 | BP | GO:0002418 | immune response to tumor cell | 2/276 | 31/29008 | 0.034992569 | 0.258548553 | 0.228507065 | Crtam/Pvr | 2 |
| GO:0009162 | BP | GO:0009162 | deoxyribonucleoside monophosphate metabolic process | 2/276 | 31/29008 | 0.034992569 | 0.258548553 | 0.228507065 | Gda/Xdh | 2 |
| GO:0015721 | BP | GO:0015721 | bile acid and bile salt transport | 2/276 | 31/29008 | 0.034992569 | 0.258548553 | 0.228507065 | Nr1h4/Slc10a6 | 2 |
| GO:0033137 | BP | GO:0033137 | negative regulation of peptidyl-serine phosphorylation | 2/276 | 31/29008 | 0.034992569 | 0.258548553 | 0.228507065 | Ddit4/Ggnbp2 | 2 |
| GO:0043276 | BP | GO:0043276 | anoikis | 2/276 | 31/29008 | 0.034992569 | 0.258548553 | 0.228507065 | Bcl2l1/Itga5 | 2 |
| GO:1905523 | BP | GO:1905523 | positive regulation of macrophage migration | 2/276 | 31/29008 | 0.034992569 | 0.258548553 | 0.228507065 | P2ry12/Rarres2 | 2 |
| GO:2000648 | BP | GO:2000648 | positive regulation of stem cell proliferation | 2/276 | 31/29008 | 0.034992569 | 0.258548553 | 0.228507065 | Cxcl1/Hmga2 | 2 |
| GO:0009072 | BP | GO:0009072 | aromatic amino acid family metabolic process | 2/276 | 32/29008 | 0.037095675 | 0.268046568 | 0.236901478 | Hgd/Iyd | 2 |
| GO:0048011 | BP | GO:0048011 | neurotrophin TRK receptor signaling pathway | 2/276 | 32/29008 | 0.037095675 | 0.268046568 | 0.236901478 | Ddit4/Ulk1 | 2 |
| GO:0071425 | BP | GO:0071425 | hematopoietic stem cell proliferation | 2/276 | 32/29008 | 0.037095675 | 0.268046568 | 0.236901478 | Cxcl1/Prg4 | 2 |
| GO:0002478 | BP | GO:0002478 | antigen processing and presentation of exogenous peptide antigen | 2/276 | 33/29008 | 0.039246095 | 0.274011178 | 0.242173044 | Fcgr3/Ifi30 | 2 |
| GO:0018345 | BP | GO:0018345 | protein palmitoylation | 2/276 | 33/29008 | 0.039246095 | 0.274011178 | 0.242173044 | Clip3/Zdhhc14 | 2 |
| GO:0048566 | BP | GO:0048566 | embryonic digestive tract development | 2/276 | 33/29008 | 0.039246095 | 0.274011178 | 0.242173044 | Foxf1/Rarres2 | 2 |
| GO:0048841 | BP | GO:0048841 | regulation of axon extension involved in axon guidance | 2/276 | 33/29008 | 0.039246095 | 0.274011178 | 0.242173044 | Sema3a/Sema6b | 2 |
| GO:0055094 | BP | GO:0055094 | response to lipoprotein particle | 2/276 | 33/29008 | 0.039246095 | 0.274011178 | 0.242173044 | Akt1/Cd68 | 2 |
| GO:1902275 | BP | GO:1902275 | regulation of chromatin organization | 2/276 | 33/29008 | 0.039246095 | 0.274011178 | 0.242173044 | Morc2a/Resf1 | 2 |
| GO:0009112 | BP | GO:0009112 | nucleobase metabolic process | 2/276 | 34/29008 | 0.041442744 | 0.279338487 | 0.246881357 | Gda/Xdh | 2 |
| GO:0036037 | BP | GO:0036037 | CD8-positive, alpha-beta T cell activation | 2/276 | 34/29008 | 0.041442744 | 0.279338487 | 0.246881357 | Crtam/Ptpn22 | 2 |
| GO:0042278 | BP | GO:0042278 | purine nucleoside metabolic process | 2/276 | 34/29008 | 0.041442744 | 0.279338487 | 0.246881357 | Gda/Xdh | 2 |
| GO:0045920 | BP | GO:0045920 | negative regulation of exocytosis | 2/276 | 34/29008 | 0.041442744 | 0.279338487 | 0.246881357 | Foxf1/Hmox1 | 2 |
| GO:0045954 | BP | GO:0045954 | positive regulation of natural killer cell mediated cytotoxicity | 2/276 | 34/29008 | 0.041442744 | 0.279338487 | 0.246881357 | Crtam/Pvr | 2 |
| GO:0090322 | BP | GO:0090322 | regulation of superoxide metabolic process | 2/276 | 34/29008 | 0.041442744 | 0.279338487 | 0.246881357 | Akt1/Cxcl1 | 2 |
| GO:0032369 | BP | GO:0032369 | negative regulation of lipid transport | 2/276 | 35/29008 | 0.043684556 | 0.287323622 | 0.253938677 | Akt1/Nr1h2 | 2 |
| GO:0042403 | BP | GO:0042403 | thyroid hormone metabolic process | 2/276 | 35/29008 | 0.043684556 | 0.287323622 | 0.253938677 | Ctsk/Iyd | 2 |
| GO:0071402 | BP | GO:0071402 | cellular response to lipoprotein particle stimulus | 2/276 | 35/29008 | 0.043684556 | 0.287323622 | 0.253938677 | Akt1/Cd68 | 2 |
| GO:0090162 | BP | GO:0090162 | establishment of epithelial cell polarity | 2/276 | 35/29008 | 0.043684556 | 0.287323622 | 0.253938677 | Foxf1/Msn | 2 |
| GO:2000785 | BP | GO:2000785 | regulation of autophagosome assembly | 2/276 | 35/29008 | 0.043684556 | 0.287323622 | 0.253938677 | Nprl2/Ulk1 | 2 |
| GO:0002717 | BP | GO:0002717 | positive regulation of natural killer cell mediated immunity | 2/276 | 36/29008 | 0.045970478 | 0.296765932 | 0.262283858 | Crtam/Pvr | 2 |
| GO:0009154 | BP | GO:0009154 | purine ribonucleotide catabolic process | 2/276 | 36/29008 | 0.045970478 | 0.296765932 | 0.262283858 | Gda/Xdh | 2 |
| GO:0060292 | BP | GO:0060292 | long-term synaptic depression | 2/276 | 36/29008 | 0.045970478 | 0.296765932 | 0.262283858 | Ager/Bcl2l1 | 2 |
| GO:0060441 | BP | GO:0060441 | epithelial tube branching involved in lung morphogenesis | 2/276 | 36/29008 | 0.045970478 | 0.296765932 | 0.262283858 | Foxf1/Hmga2 | 2 |
| GO:0007435 | BP | GO:0007435 | salivary gland morphogenesis | 2/276 | 37/29008 | 0.048299477 | 0.303489528 | 0.268226221 | Fgfr1/Sema3a | 2 |
| GO:0016572 | BP | GO:0016572 | histone phosphorylation | 2/276 | 37/29008 | 0.048299477 | 0.303489528 | 0.268226221 | Hmga2/Pim3 | 2 |
| GO:0048846 | BP | GO:0048846 | axon extension involved in axon guidance | 2/276 | 37/29008 | 0.048299477 | 0.303489528 | 0.268226221 | Sema3a/Sema6b | 2 |
| GO:0060218 | BP | GO:0060218 | hematopoietic stem cell differentiation | 2/276 | 37/29008 | 0.048299477 | 0.303489528 | 0.268226221 | Lmbr1l/Pus7 | 2 |
| GO:1902284 | BP | GO:1902284 | neuron projection extension involved in neuron projection guidance | 2/276 | 37/29008 | 0.048299477 | 0.303489528 | 0.268226221 | Sema3a/Sema6b | 2 |
| GO:2000191 | BP | GO:2000191 | regulation of fatty acid transport | 2/276 | 37/29008 | 0.048299477 | 0.303489528 | 0.268226221 | Acsl6/Akt1 | 2 |
| GO:0031414 | CC | GO:0031414 | N-terminal protein acetyltransferase complex | 2/268 | 11/28886 | 0.004463239 | 0.242964068 | 0.231304802 | Naa25/Naa38 | 2 |
| GO:0000109 | CC | GO:0000109 | nucleotide-excision repair complex | 2/268 | 13/28886 | 0.006252752 | 0.242964068 | 0.231304802 | Cetn2/Slx4 | 2 |
| GO:0030688 | CC | GO:0030688 | preribosome, small subunit precursor | 2/268 | 13/28886 | 0.006252752 | 0.242964068 | 0.231304802 | Riok1/Riok2 | 2 |
| GO:0031527 | CC | GO:0031527 | filopodium membrane | 2/268 | 17/28886 | 0.010639424 | 0.254484318 | 0.242272222 | Msn/Ninj1 | 2 |
| GO:0005669 | CC | GO:0005669 | transcription factor TFIID complex | 2/268 | 31/28886 | 0.033419475 | 0.431208931 | 0.410516242 | Taf7l/Tbp | 2 |
| GO:0008305 | CC | GO:0008305 | integrin complex | 2/268 | 33/28886 | 0.037493145 | 0.431208931 | 0.410516242 | Itga5/Itgbl1 | 2 |
| GO:0032588 | CC | GO:0032588 | trans-Golgi network membrane | 2/268 | 38/28886 | 0.048443775 | 0.431208931 | 0.410516242 | Clip3/Rab31 | 2 |
| GO:0034364 | CC | GO:0034364 | high-density lipoprotein particle | 2/268 | 38/28886 | 0.048443775 | 0.431208931 | 0.410516242 | Hp/Saa1 | 2 |
| GO:0098636 | CC | GO:0098636 | protein complex involved in cell adhesion | 2/268 | 38/28886 | 0.048443775 | 0.431208931 | 0.410516242 | Itga5/Itgbl1 | 2 |
| GO:0010181 | MF | GO:0010181 | FMN binding | 2/273 | 15/28438 | 0.008878128 | 0.279512649 | 0.255308464 | Hao1/Iyd | 2 |
| GO:0045236 | MF | GO:0045236 | CXCR chemokine receptor binding | 2/273 | 15/28438 | 0.008878128 | 0.279512649 | 0.255308464 | Cxcl1/Pf4 | 2 |
| GO:0004698 | MF | GO:0004698 | calcium-dependent protein kinase C activity | 2/273 | 16/28438 | 0.010082621 | 0.279512649 | 0.255308464 | Prkcg/Prkd3 | 2 |
| GO:0048019 | MF | GO:0048019 | receptor antagonist activity | 2/273 | 16/28438 | 0.010082621 | 0.279512649 | 0.255308464 | Dkk3/Wfikkn1 | 2 |
| GO:0004697 | MF | GO:0004697 | protein kinase C activity | 2/273 | 17/28438 | 0.011355162 | 0.282518209 | 0.258053759 | Prkcg/Prkd3 | 2 |
| GO:0052745 | MF | GO:0052745 | inositol phosphate phosphatase activity | 2/273 | 18/28438 | 0.012694346 | 0.282518209 | 0.258053759 | Bpnt1/Inpp5d | 2 |
| GO:0022884 | MF | GO:0022884 | macromolecule transmembrane transporter activity | 2/273 | 21/28438 | 0.017097975 | 0.282518209 | 0.258053759 | Sidt2/Timm22 | 2 |
| GO:0033130 | MF | GO:0033130 | acetylcholine receptor binding | 2/273 | 21/28438 | 0.017097975 | 0.282518209 | 0.258053759 | Ly6i/Psca | 2 |
| GO:0030215 | MF | GO:0030215 | semaphorin receptor binding | 2/273 | 22/28438 | 0.018690056 | 0.282518209 | 0.258053759 | Sema3a/Sema6b | 2 |
| GO:0046965 | MF | GO:0046965 | retinoid X receptor binding | 2/273 | 22/28438 | 0.018690056 | 0.282518209 | 0.258053759 | Nr1h2/Nr1h4 | 2 |
| GO:0009931 | MF | GO:0009931 | calcium-dependent protein serine/threonine kinase activity | 2/273 | 23/28438 | 0.020342044 | 0.289586114 | 0.264509625 | Prkcg/Prkd3 | 2 |
| GO:0010857 | MF | GO:0010857 | calcium-dependent protein kinase activity | 2/273 | 24/28438 | 0.02205265 | 0.289586114 | 0.264509625 | Prkcg/Prkd3 | 2 |
| GO:0031683 | MF | GO:0031683 | G-protein beta/gamma-subunit complex binding | 2/273 | 24/28438 | 0.02205265 | 0.289586114 | 0.264509625 | Cetn2/Gnal | 2 |
| GO:0019239 | MF | GO:0019239 | deaminase activity | 2/273 | 26/28438 | 0.025644662 | 0.306311797 | 0.27978696 | Adarb1/Gda | 2 |
| GO:0008266 | MF | GO:0008266 | poly(U) RNA binding | 2/273 | 27/28438 | 0.027523586 | 0.306311797 | 0.27978696 | Cirbp/Pabpc1l | 2 |
| GO:0000146 | MF | GO:0000146 | microfilament motor activity | 2/273 | 28/28438 | 0.029456165 | 0.306311797 | 0.27978696 | Myo9b/Tnnt2 | 2 |
| GO:0030247 | MF | GO:0030247 | polysaccharide binding | 2/273 | 28/28438 | 0.029456165 | 0.306311797 | 0.27978696 | Fcna/Prg4 | 2 |
| GO:0005310 | MF | GO:0005310 | dicarboxylic acid transmembrane transporter activity | 2/273 | 29/28438 | 0.031441203 | 0.306311797 | 0.27978696 | Slc25a18/Slc26a8 | 2 |
| GO:0016814 | MF | GO:0016814 | hydrolase activity, acting on carbon-nitrogen (but not peptide) bonds, in cyclic amidines | 2/273 | 29/28438 | 0.031441203 | 0.306311797 | 0.27978696 | Adarb1/Gda | 2 |
| GO:0043325 | MF | GO:0043325 | phosphatidylinositol-3,4-bisphosphate binding | 2/273 | 29/28438 | 0.031441203 | 0.306311797 | 0.27978696 | Akt1/Gab2 | 2 |
| GO:0045499 | MF | GO:0045499 | chemorepellent activity | 2/273 | 29/28438 | 0.031441203 | 0.306311797 | 0.27978696 | Sema3a/Sema6b | 2 |
| GO:0008187 | MF | GO:0008187 | poly-pyrimidine tract binding | 2/273 | 31/28438 | 0.035563967 | 0.316900347 | 0.289458602 | Cirbp/Pabpc1l | 2 |
| GO:0016251 | MF | GO:0016251 | RNA polymerase II general transcription initiation factor activity | 2/273 | 32/28438 | 0.03769939 | 0.324344751 | 0.296258364 | Taf7l/Tbp | 2 |
| GO:0098632 | MF | GO:0098632 | cell-cell adhesion mediator activity | 2/273 | 32/28438 | 0.03769939 | 0.324344751 | 0.296258364 | Cd200r1/Ninj1 | 2 |
| GO:0000993 | MF | GO:0000993 | RNA polymerase II complex binding | 2/273 | 37/28438 | 0.049072416 | 0.373481237 | 0.341139913 | Scaf1/Zfp326 | 2 |
| GO:0046915 | MF | GO:0046915 | transition metal ion transmembrane transporter activity | 2/273 | 37/28438 | 0.049072416 | 0.373481237 | 0.341139913 | Slc25a28/Slc39a14 | 2 |
| GO:0140223 | MF | GO:0140223 | general transcription initiation factor activity | 2/273 | 37/28438 | 0.049072416 | 0.373481237 | 0.341139913 | Taf7l/Tbp | 2 |
