# Supplementary material for: Authentication and validation of key genes in the treatment of atopic dermatitis with Runfuzhiyang powder: combined RNA-seq, bioinformatics analysis, and experimental research
Source: Front Genet. 2024 Aug 1;15:1335093. doi: 10.3389/fgene.2024.1335093 (PMC11324508; doi:10.3389/fgene.2024.1335093)
Supplement: Supplementary file 3 [file Table3.DOCX]

Link of Supplementary Figure1

<https://www.jianguoyun.com/p/DUsngWsQ--LFDBin274FIAA>

Link of Supplementary Figure2

https://www.jianguoyun.com/p/Dfxrln8Q--LFDBiq274FIAA

Link of Supplementary Figure3

https://www.jianguoyun.com/p/DRVs-kcQ--LFDBit274FIAA

Link of Supplementary Figure4

https://www.jianguoyun.com/p/DdvkysAQ--LFDBiu274FIAA

Link of Supplementary Figure5

<https://www.jianguoyun.com/p/DZCFNqwQ--LFDBiv274FIAA>

Link of Supplementary Figure6

https://www.jianguoyun.com/p/DTNBct4Q--LFDBiw274FIAA

Link of Supplementary Figure7

https://www.jianguoyun.com/p/DUubFUIQ--LFDBix274FIAA

Link of Supplementary Figure8

https://www.jianguoyun.com/p/DQUg_Y0Q--LFDBiy274FIAA

Link of Supplementary Figure9

https://www.jianguoyun.com/p/DS9_ipoQ--LFDBiz274FIAA

Link of Supplementary Figure10

https://www.jianguoyun.com/p/DS2Opq0Q--LFDBi0274FIAA
